# Hydrogen atom abstraction as a synthetic route to a square planar Co^II^ complex with a redox-active tetradentate PNNP ligand†

**DOI:** 10.1039/d4sc03364g

**Published:** 2024-08-30

**Authors:** Justin D. Miller, Mitchell M. Walsh, Kyounghoon Lee, Curtis E. Moore, Christine M. Thomas

**Affiliations:** a Department of Chemistry and Biochemistry, The Ohio State University 100 W. 18th Ave Columbus OH 43210 USA thomasc@chemistry.ohio-state.edu; b Department of Chemical Education and Research Institute of Natural Sciences, Gyeongsang National University Gyeongnam 52828 Republic of Korea

## Abstract

Redox-active ligands improve the reactivity of transition metal complexes by facilitating redox processes independent of the transition metal center. A tetradentate square planar (PNCH_2_CH_2_NP)Co^II^ (1) complex was synthesized and the ethylene backbone was dehydrogenated through hydrogen atom abstraction to afford (PNCHCHNP)Co^II^ (2), which now contains a redox-active ligand. The ligand backbone of 2 can be readily hydrogenated with H_2_ to regenerate 1. Reduction of 1 and 2 with KC_8_ in the presence of 18-crown-6 results in cobalt-based reductions to afford [(PNCH_2_CH_2_NP)Co^I^][K(18-crown-6)] (3) and [(PNCHCHNP)Co^I^][K(18-crown-6)] (4), respectively. Cyclic voltammetry revealed two reversible oxidation processes for 2, presumed to be ligand-based. Following treatment of 2 with one equivalent of FcPF_6_, the one-electron oxidation product {[(PNCHCHNP)Co^II^(THF)][PF_6_]}·THF (5) was obtained. Treating 5 with an additional equivalent of FcPF_6_ affords the two-electron oxidation product [(PNCHCHNP)Co^II^][PF_6_]_2_ (6). Addition of PMe_3_ to 5 produced [(PNCHCHNP)Co^II^(PMe_3_)][PF_6_] (7). A host of characterization methods including nuclear magnetic resonance (NMR) spectroscopy, electron paramagnetic resonance (EPR) spectroscopy, cyclic voltammetry, magnetic susceptibility measurements using SQUID magnetometry, single-crystal X-ray diffraction, and density functional theory calculations were used to assign 5 and 6 as ligand-based oxidation products of 2.

## Abstract 2

Dehydrogenation of the ligand backbone of a bis(amido)bis(phosphine) Co complex is achieved through hydrogen atom abstraction. The new unsaturated backbone of the tetradentate ligand renders the ligand in the resulting Co complex redox-active.

## Graphical abstract

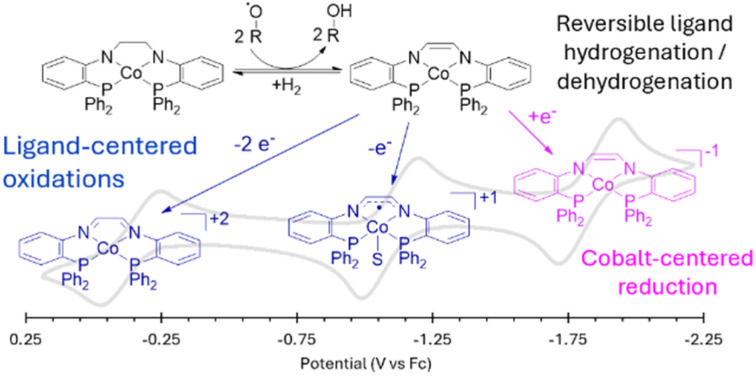

## Introduction

**Scheme 1:**
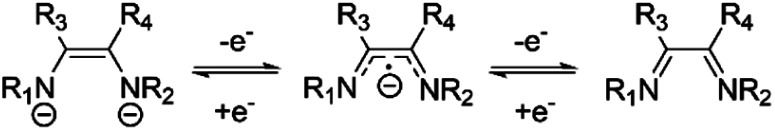
Three possible redox states of diimine/enediamide ligands.

**Fig. 1:**
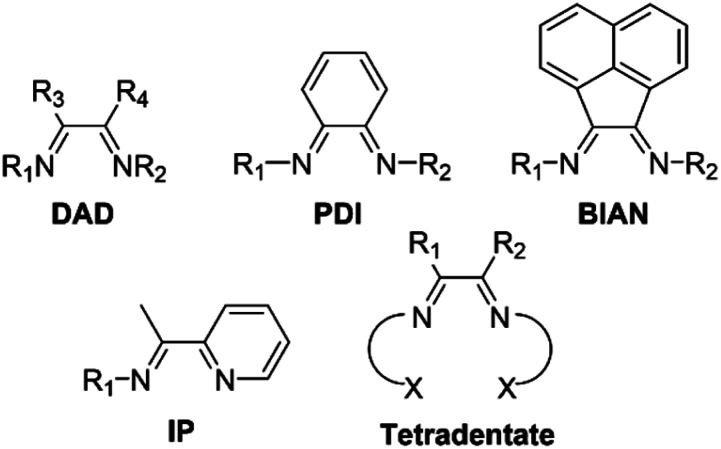
Common examples of redox-active diimine ligands in the literature.

Redox-active ligands have emerged as a strategy to facilitate oxidative and reductive reactivity in transition metal complexes by acting as electron reservoirs.^1–6^ A diverse set of reactivity has been exploited using redox-active ligands, such as cross-coupling, hydroelementation, small molecule activation, and hydrogen evolution reactions.^2,4,7–10^ While a variety of structural motifs provide access to redox-active ligands, one class of redox-active ligands incorporates a diimine/enediamide moiety. The reversible storage of electrons within this framework stems from the interconversion between the diimine and enediamide structures, permitting access to three potential redox states (Scheme 1). A variety of bidentate diimine ligand systems have been realized and coordinated to transition metals, including diazadienes (DADs),^11–17^ phenylenediamines (PDIs),^18–26^ bis(imino)acenaphthenes (BIANs),^27–33^ and iminopyridines (IPs)^34–38^ (Fig. 1). Further derivatization of the bidentate analogues affords tetradentate redox-active ligands with the general formula XNNX (X = O, SMe_2_, PPh_2_, NMe_2_) displayed in Fig. 1.^39–50^

Modifications of the side-arm heteroatoms in tetradentate ligands incorporating the previously discussed diimine/enediamide backbone provides an additional site for tuning the electronic environment of the metal and/or ligand.

This is exemplified in a report by Daly and coworkers where an anodic shift in the ligand-centered oxidation potentials and a change in reversibility of the nickel-centered reduction were observed when the side-arm substituents were switched from NMe_2_ to SMe.^43^ Additionally, Daly *et al.* found that placement of redox-innocent NMe_2_ groups in the sidearm positions shifted the location of the ligand-based redox activity to the central diamide position of the ligand,^43^ which contrasts to the sidearm-based redox activity reported by Thomas in 2016 using amide (NH) sidearm substituents.^51^ With ligand modifications resulting in significant changes in electronic properties, synthesizing and investigating the electronic structures of new tetradentate redox-active ligand frameworks will provide more tools for tuning reactivity. Changing the identity of the metal also influences the electronic properties of organometallic complexes with redox-active ligands. van Slageren, Sarkar, and coworkers investigated bis(sulfonamido)benzene complexes of Co, Ni, and Fe where only the Fe complex was shown to exhibit a metal-centered oxidation (Fe^II/III^) while oxidations of the Co^II^ and Ni^II^ complexes were ligand-centered.^18^ While there are a variety of redox-active diimine/enediamide complexes, examples with Co are limited^49,52^ and require further investigation.

**Scheme 2:**
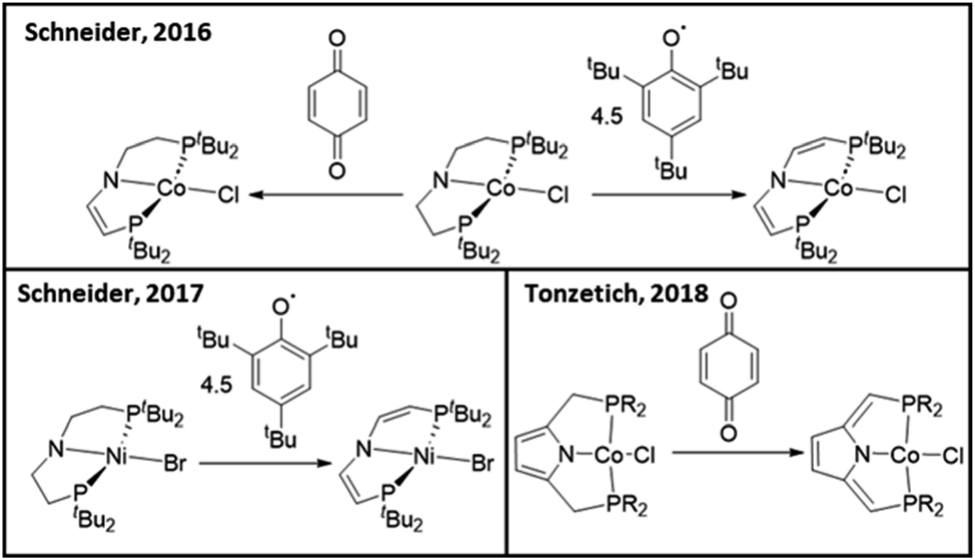
Previous examples of post-metalation hydrogen atom abstraction methods for ligand dehydrogenation.

Although much attention has been focussed on the electronic properties of redox-active ligands, limited research has explored unique synthetic routes towards redox-active ligands. Typically, redox-active diimine/enediamide complexes are synthesized through (1) metalation/deprotonation of enediamine precursors,^18–21,23–25,43,50^ (2) direct metalation of diimine ligands,^15,31,32,44,45^ or (3) reduction of a diimine ligand in the presence of a metal source.^13,14,16,36,53^ In recent years, a post-metalation hydrogen atom abstraction strategy has been used to dehydrogenate the ligand backbones of Co and Ni complexes (Scheme 2).^54–56^ Backbone dehydrogenation was shown to provide access to ligand-based oxidation processes.^54,56^ Although there are a few examples of post-metalation hydrogen atom abstraction methods to incorporate ligand unsaturation, further exploration into the ligand motifs and metals amenable to this transformation could open new synthetic avenues towards redox-active ligands.

This work will discuss the synthesis and characterization of seven cobalt complexes incorporating a tetradentate [PNNP]^2−^ ligand, including those with an unsaturated enediamide ligand backbone that render the ligand redox-active. The H_2_[PNNP] ligand precursor was reported in 2011,^57^ and it has since been bound in its deprotonated dianionic form to Pt,^58^ Ni,^59^ Fe,^60^ Cu, Ge,^58^ Sn,^58^ Mg,^61^ Ca,^61^ Sr,^61^ Al,^61^ and Zn.^61^ Herein, we explore the coordination of this ligand to Co and the redox properties and reactivity of the resulting compound, (PNCH_2_CH_2_NP)Co (1), including post-metallation hydrogen atom abstraction from the ligand backbone. Following dehydrogenation of the backbone of 1, two reversible ligand-based oxidation processes are accessible. Single crystal X-ray diffraction, magnetic measurements, electron paramagnetic resonance (EPR), and density functional theory (DFT) analysis support the hypothesis that the oxidative processes are localized on the ligand rather than the Co center. Herein, we report a unique hydrogen atom abstraction route to afford the first Co complex incorporating a redox-active tetradentate diimine/enediamide complex with phosphine sidearms.

## Results and discussion

### Synthesis and characterization of (PNCH_2_CH_2_NP)Co and (PNCHCHNP)Co

**Scheme 3:**
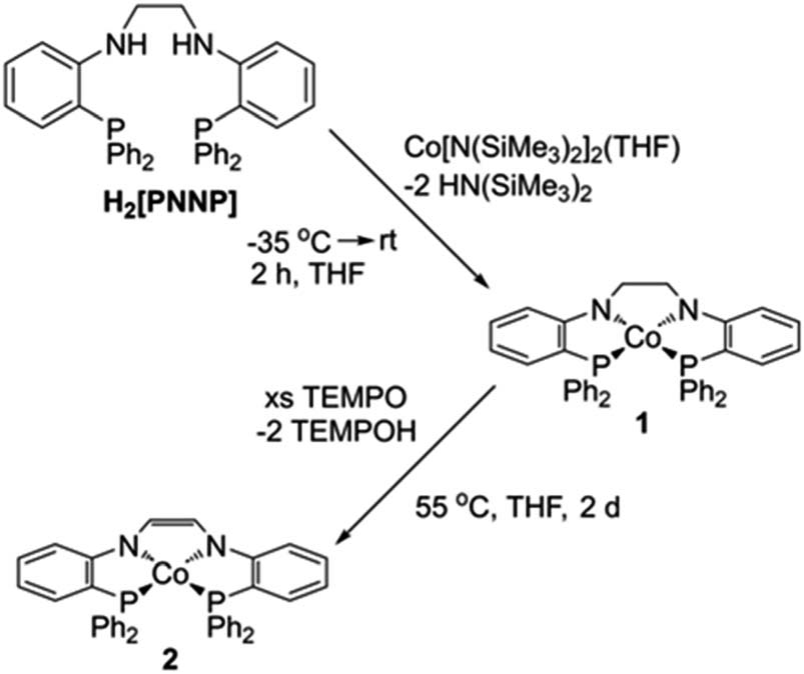
Synthesis of 1 and 2.

To begin our investigations into the non-innocent nature of the [PNNP]^2−^ ligand platform, (PNCH_2_CH_2_NP)Co (1) was synthesized through simultaneous deprotonation and coordination by treating H_2_[PNNP]^57^ with one equivalent of Co[N(SiMe_3_)_2_]_2_(THF)^62^ (Scheme 3). Following workup, 1 was isolated in 88% yield as an olive-green solid. The ^1^H NMR spectrum of 1 (Fig. S1†) displays eight distinct paramagnetically shifted resonances between 145 ppm and −16 ppm, consistent with the expected *C*_2v_ symmetry and square planar geometry of 1.

Motivated by previous literature investigations that demonstrated successful hydrogen atom abstraction from ligand frameworks using a variety of hydrogen atom abstracting reagents,^55,56^ we sought to formally dehydrogenate the ligand backbone of 1 using a hydrogen atom acceptor. To our delight, addition of excess 2,4,6-tri-*tert*-butylphenoxy radical to 1 at room temperature resulted in formation of a new paramagnetic product, (PNCHCHNP)Co (2) (Fig. S3†). The modest yield (58%), low purity, and the lengthy synthesis of the 2,4,6-tri-*tert*-butylphenoxy radical prompted investigation into a new synthetic route. Addition of excess (2,2,6,6-tetramethylpiperidine-1-yl)oxyl (TEMPO) to 1 at 55 °C afforded 2 as a red solid in 73% yield (Scheme 3). The route using TEMPO was used for large-scale preparations of 2 due to higher yields and the commercial availability of TEMPO. The ^1^H NMR spectrum of 2 displays eight distinct paramagnetically shifted resonances, consistent with the expected *C*_2v_ symmetry for a square planar complex (Fig. S2†).

**Fig. 2:**
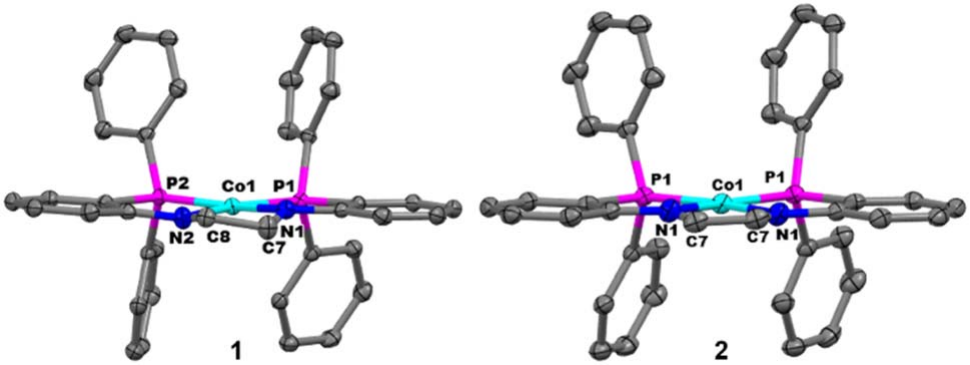
Displacement ellipsoid (50%) representations of 1 (left) and 2 (right). All H atoms and solvate molecules are omitted for clarity.

Crystals of 1 suitable for single crystal X-ray diffraction were obtained by vapor diffusion of pentane into a saturated benzene solution of 1. The solid-state structure of 1 reveals a square planar geometry (*τ*_4_ = 0.15)^63^ about the Co^II^ center, which is ligated to [PNNP]^2−^ through two amides and two phosphines in a *κ*^4^ coordination mode (Fig. 2). Relevant bond distances and dihedral angles are shown in Table 1. The average Co–N and Co–P bond distances, 1.871(2) Å and 2.1931(7) Å, respectively, are consistent with previously reported square planar bis(phosphine) bis(amido) Co^II^ complexes.^64–66^ The C7–C8 bond distance in the ethylene backbone (1.522(2) Å) and a N2–C8–C7–N1 dihedral angle of 26.1(2)° supports the assignment of sp^3^ hybridized carbon atoms in the backbone.

**Table 1:** Relevant bond lengths and angles for complexes 1–7. The C–C bond distance was measured between the carbon atoms bridging the nitrogen atoms (*e.g.* C7–C8 in 1 in Fig. 2). The C–N distance is in reference to the distance between the nitrogen atoms and the carbon atoms bridging the nitrogen atoms (*e.g.* N2–C8 and N1–C7 in 1 in Fig. 2)

	Co–N_avg_ (Å)	Co–P_avg_ (Å)	C–C (Å)	C–N_avg_ (Å)	N–C–C–N (°)
1	1.871(2)	2.1931(7)	1.522(2)	1.451(3)	26.1(2)
2	1.878(2)	2.1866(7)	1.396(5)	1.403(3)	−9.10(1)
3^a^	1.896(7)	2.113(2)	1.503(10)	1.459(11)	−22.9(7)
4	1.897(2)	2.1150(9)	1.349(3)	1.399(4)	2.2(3)
5	1.908(4)	2.1916(15)	1.391(6)	1.337(7)	−1.2(5)
6	1.930(4)	2.1532(14)	1.450(5)	1.288(7)	−1.1(6)
7	1.8864(18)	2.1897(6)	1.378(2)	1.350(3)	−0.2(2)

a Two molecules were located in the asymmetric unit of 3, therefore, the C–C bond distance and N–C–C–N dihedral angles were obtained from an average of the two molecules.

Crystals obtained from the reaction solution used to generate 2 were suitable for single crystal X-ray diffraction. The solid-state structure of 2 is shown in Fig. 2 with bond distances and dihedral angles displayed in Table 1. The cobalt center in 2 adopts a square planar geometry (*τ*_4_ = 0.15) and a similar coordination environment to 1. The Co–N and Co–P bond distances in 2 (1.878(2) Å and 2.1866(7) Å, respectively) do not differ significantly from 1, demonstrating a negligible difference in the electronic environments of the two Co centers. Contrary to the structure of 1, the backbone of 2 displays a shorter C–C bond distance (1.396(5) Å), consistent with a double bond, and a more planar N–C–C–N dihedral angle (−9.10(1)°) confirming dehydrogenation of the ligand backbone.

**Scheme 4:**
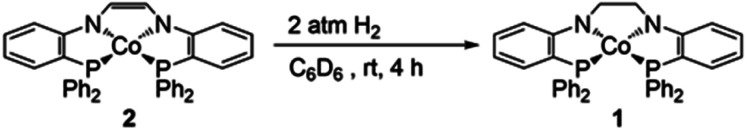
Hydrogenation of the backbone of 2.

Since the abstraction of two hydrogen atoms from the backbone of 1 to generate 2 represents a formal dehydrogenation process, we investigated whether this process was reversible *via* hydrogenation of the ligand backbone of 2. Addition of H_2_ (∼2 atm) to a C_6_D_6_ solution of 2 in a J. Young tube resulted in a color change from red-orange to brown within 4 hours (Scheme 4). ^1^H NMR spectroscopy revealed complete conversion to 1*via* hydrogenation of the ligand backbone (Fig. S19†).

Since previous examples of post-metalation ligand backbone dehydrogenation *via* treatment with hydrogen atom abstraction reagents were successful with both Co and Ni,^54–56^ we sought to explore whether the dehydrogenation of the ethylene backbone of 1 was specific to Co. Treatment of the previously reported Ni analogue of 1, (PNCH_2_CH_2_NP)Ni (1-Ni),^59^ with TEMPO (BDFE_O–H_ = 65.2 kcal mol^−1^)^67^ resulted in no reaction whereas treatment with 2.5 equiv. 2,4,6-tri-*tert*-butylphenoxy radical (BDFE_O–H_ = 76.7 kcal mol^−1^)^67^ led to incomplete conversion to the ligand dehydrogenation product, (PNCHCHNP)Ni (2-Ni) (Fig. S23†). Addition of excess 2,4,6-tri-*tert*-butylphenoxy radical to 1-Ni led to complete conversion to 2-Ni, but hydrogen abstraction was found to be reversible, leading to regeneration of 1-Ni upon attempts to purify the dehydrogenated product. From these data, it can be concluded that the BDFE of the backbone C–H bonds of 1-Ni is higher than that of the Co analogue 1. Since little variability is expected in the p*K*_a_ of the backbone C–H bonds or the driving force for forming a new C=C bond as a function of metal identity, the difference in reactivity between 1 and 1-Ni is attributed to the differences in redox potentials of the Co and Ni species; the first oxidation potential of 1 is 270 mV lower than that of 1-Ni (Fig. S26† and 3, *vide infra*).

**Fig. 3:**
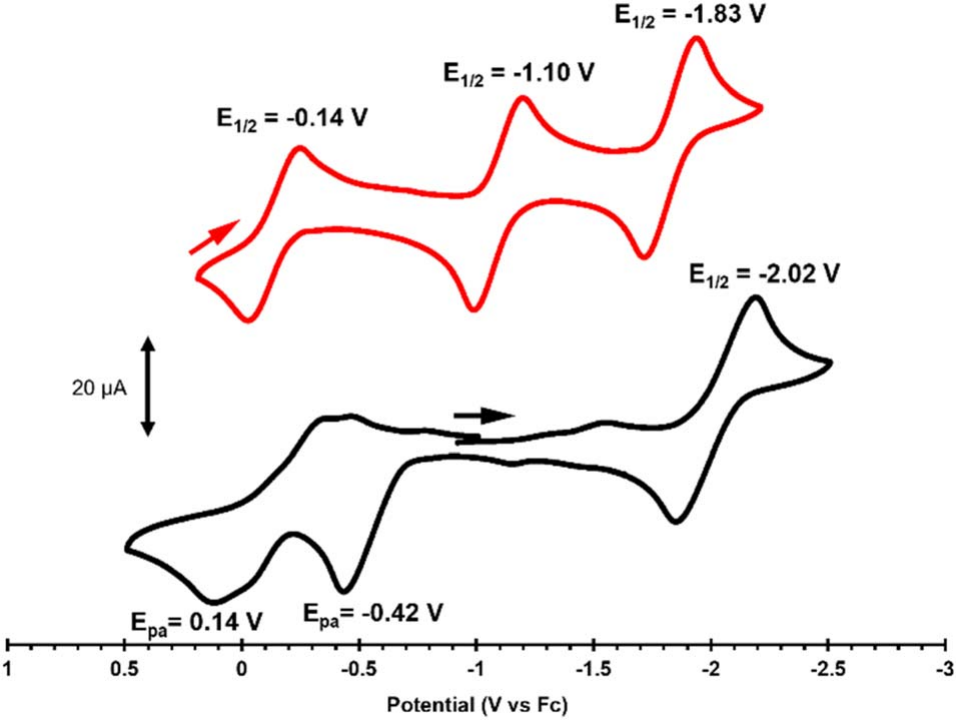
Cyclic voltammograms of 1 (bottom, black) and 2 (top, red) in 0.1 M [^*n*^Bu_4_N][PF_6_] solution (scan rate = 100 mV s^−1^). All potentials are referenced to Fc/Fc^+^.

### Electrochemical analysis of 1 and 2

Cyclic voltammetry measurements were performed to analyze the ligand- and metal-based redox processes of 1 and 2. The cyclic voltammograms (CVs) of 1 and 2 (Fig. 3) display reversible reductions assigned to Co^II/I^ redox couples at *E*_1/2_ = −2.02 V and −1.83 V (*vs.* Fc/Fc^+^, where Fc = Cp_2_Fe), respectively.

The more facile reduction of 2 is the result of the less electron-rich unsaturated ligand backbone. The CVs reveal much more significant differences in the oxidative processes of 1 and 2. The CV of 1 features two irreversible oxidations at *E*_pa_ = −0.42 V and *E*_pa_ = 0.14 V (*vs.* Fc/Fc^+^), while the CV of 2 displays two cathodically shifted reversible oxidations at *E*_1/2_ = −1.10 V and −0.14 V (*vs.* Fc/Fc^+^). The large differences in potential and reversibility of the two oxidative features in the CV suggest that these may be assigned to ligand-based L^2−^/L˙^−^ and L˙^−^/L processes, respectively. The presence of two reversible ligand-based redox processes is observed in Co complexes with two redox-active *o*-diiminoquinone ligands.^18,23^ When a single diimine/diamide subunit is bound to a transition metal center, a single reversible ligand-based redox process^13,15,19,39^ or one two-electron ligand-based redox process^21,25^ is generally observed. Few examples exist of Co complexes coordinated to a single redox-active ligand, with a similar motif to 2, that display two reversible ligand-based redox processes.^68,69^

### Chemical reduction of 1 and 2

**Scheme 5:**
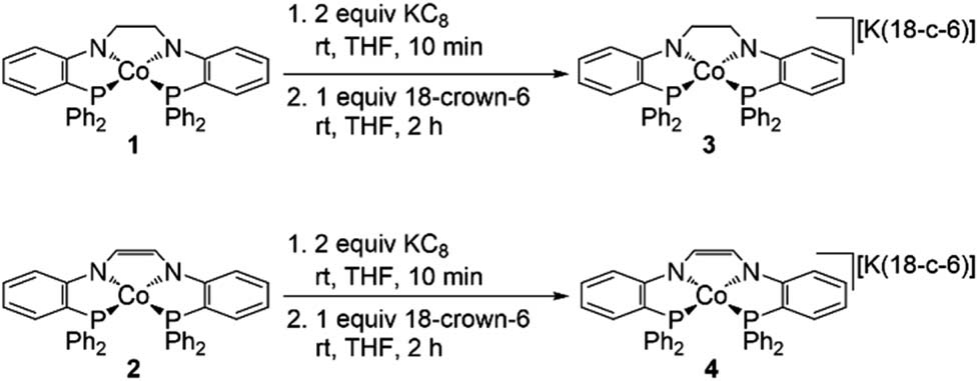
Synthesis of 3 and 4.

To confirm the assignment of a Co^II/I^ redox couple for the reductions observed in the CVs of 1 and 2, each complex was treated with a chemical reductant. Reduction of 1 with two equiv. KC_8_ results in a color change from brown to green. Addition of one equiv. 18-crown-6 to the solution affords [(PNCH_2_CH_2_NP)Co][K(18-crown-6)] (3) in 96% yield as a black solid (Scheme 5). Similarly, reduction of 2 with two equiv. KC_8_ results in a color change from red-orange to brown and addition of one equiv. 18-crown-6 to the solution affords [(PNCHCHNP)Co][K(18-crown-6)] (4) in 89% yield as a black solid (Scheme 5). NMR spectroscopy revealed ^31^P{^1^H} signals at 59 ppm and diamagnetic spectra for 3 and 4 consistent with *C*_2v_-symmetric square planar Co^I^ complexes (Fig. S4–S13†).

**Fig. 4:**
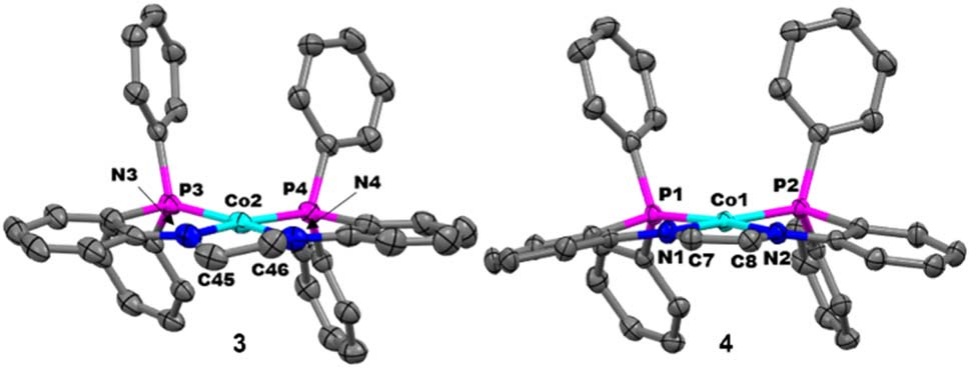
Displacement ellipsoid (50%) representations of 3 (left) and 4 (right). All H atoms, solvate molecules, and K^+^ counterions encapsulated by 18-crown-6 were removed for clarity. There are two independent molecules in the asymmetric unit of 3, but only one is shown for clarity.

Crystals of 3 and 4 suitable for X-ray diffraction were obtained through the vapor diffusion of pentane into a saturated benzene solution of 3 or 4 at room temperature and the resulting structures are displayed in Fig. 4, with relevant bond distances and dihedral angles displayed in Table 1. The solid-state structure of 3 contains two independent metal complexes occupying the asymmetric unit, therefore, all structural metrics were obtained from an average of the two molecules. The Co center of 3 adopts a square planar geometry (*τ*_4_ = 0.15). Each of the two potassium cations present in the asymmetric unit are ligated by an 18-crown-6 molecule. A similar square planar geometry is also observed for 4 (*τ*_4_ = 0.17) in the solid state, with a single molecule in the asymmetric unit and one K^+^ counterion encapsulated by a crown ether molecule. The C–C and C–N bond distances of 3 and 4 do not differ significantly from their neutral analogues (1 and 2), supporting a Co-centered rather than ligand-centered reduction (Table 1). There is a slight difference between the neutral (1 and 2) and anionic (3 and 4) species when comparing the Co–N and Co–P bond distances (Table 1). Decreased π-donation from the amides due to a more reduced Co center explains the slightly elongated Co–N distances, while increased π-back-bonding from Co to the phosphines leads to the shorter Co–P bond distances in the reduced species. Overall, the structural data supports the assignment of Co-based reductions.

### Chemical oxidation of 2

**Scheme 6:**
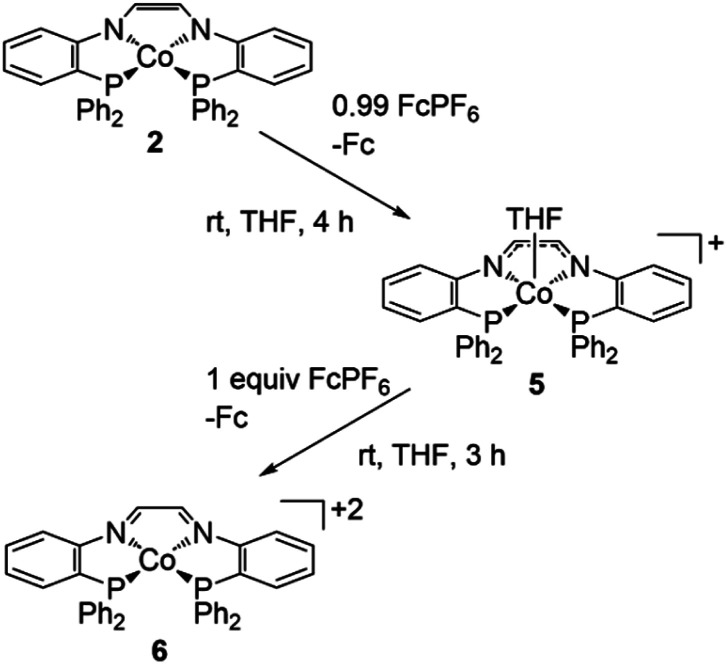
Synthesis of 5 and 6.

To discern the nature of the oxidative processes in the CV of 2, the products of its one- and two-electron oxidation were isolated and characterized. Treatment of 2 with 0.99 equivalents of FcPF_6_ in THF resulted in a rapid color change from red-orange to purple, producing the monocationic species {[(PNCHCHNP)Co(THF)][PF_6_]}·THF (5) in 85% yield (Scheme 6). The ^31^P{^1^H} spectrum of 5 in CD_2_Cl_2_ (Fig. S16†) features a septet at −143 ppm corresponding to the PF_6_ counterion and a singlet at 8.74 ppm attributed to the phosphine sidearms that is shifted substantially upfield compared to Co^I^ complex 4. The ^1^H NMR spectrum (Fig. S14†) displays eight distinct resonances between 6.5 and 11.5 ppm, consistent with a diamagnetic Co complex with *C*_2v_ symmetry. Even after rigorously drying samples of 5*in vacuo*, ^1^H NMR resonances corresponding to THF (3.61 ppm and 1.77 ppm) are observed; they are slightly shifted from free THF (3.69 ppm and 1.82 ppm) and integrate to roughly eight protons each. Variable temperature NMR spectroscopy (Fig. S18A†) suggests one THF molecule is bound in solution and rapidly exchanges with a second THF molecule. The two resonances for the bound and free THF molecules de-coalesce at 200 K and are well pronounced at 190 K. Eleven aromatic peaks are observed at 190 K (Fig. S18B†), indicating the loss of the *C*_2_ symmetry as the THF exchange is slow enough to be observed on the NMR time scale.

**Fig. 5:**
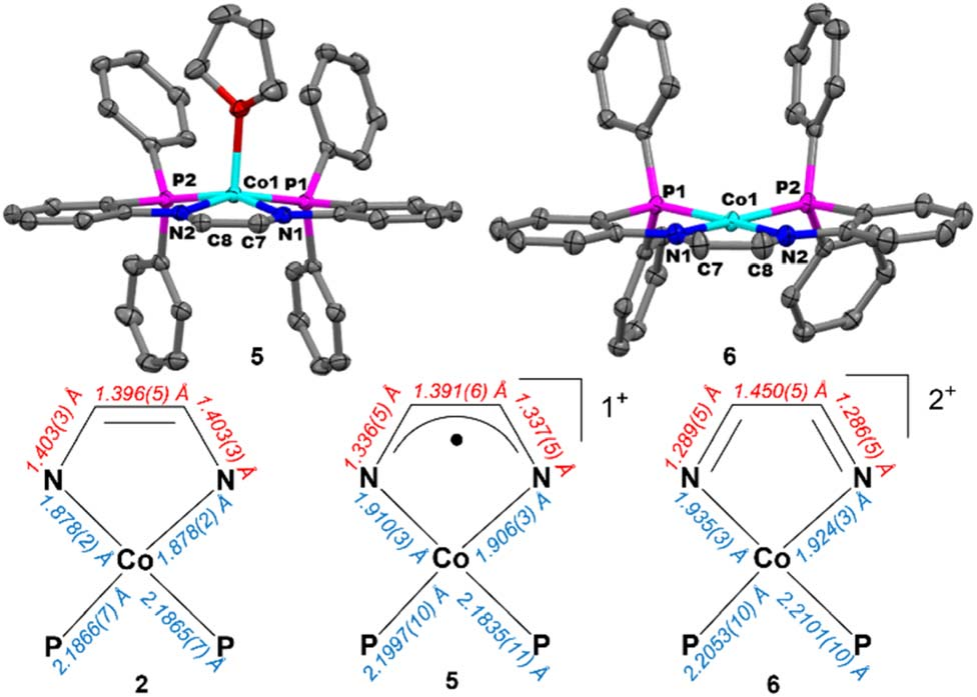
(Top) Displacement ellipsoid (50%) representations of 5 (Left) and 6 (Right). All H atoms, solvate molecules, and PF_6_^−^ anions were omitted for clarity. (Bottom) Comparison of the metal–ligand and N–C–C–N bond metrics of 2, 5, and 6.

Crystals suitable for single crystal X-ray diffraction were grown *via* vapor diffusion of Et_2_O into a concentrated THF solution of 5 at room temperature. The solid-state structure of 5 adopts a square pyramidal geometry (*τ*_5_ = 0.01)^70^ with one THF molecule occupying the axial coordination site and a second THF solvate molecule in the crystal lattice (Fig. 5). Oxidation from 2 to 5 resulted in elongated Co–N bonds (+0.030 Å), shorter C–N bonds (−0.066 Å), and minimal variations in the Co–P and C–C bond distances (+0.0050 Å and −0.005 Å, respectively) (Table 1 and Fig. 5). The change in the Co–N and C–N bond distances from 2 to 5 are consistent with a one-electron oxidized ligand in an intermediate radical anion state, intermediate between the enediamide and diimine resonance structures. In addition, the minimal change in the Co–P bond distances supports retention of the Co^II^ oxidation state. Due to the diamagnetic nature of 5 and support for an oxidized ligand bound to a Co^II^ center, we hypothesized the ground-state electronic configuration of 5 to be an open-shell singlet. To our delight, SQUID magnetometry data (*vide infra*) confirmed that 5 shows diamagnetic behaviour across all temperatures, supporting our hypothesis.

A second ligand-based oxidation can be accomplished through addition of one equivalent of FcPF_6_ to a THF solution of 5 resulting in a rapid color change from purple to green to give 6 in 85% yield (Scheme 6). Crystals suitable for single-crystal X-ray diffraction were grown by vapor diffusion of Et_2_O into a saturated THF solution of 6 at room temperature. The solid-state structure of 6 reveals a square planar geometry about the Co center (*τ*_4_ = 0.16) with two PF_6_^−^ anions and a THF solvate molecule in the crystal lattice (Fig. 5). Compared to 5, the Co–N (+0.022 Å) and C–C (+0.059 Å) bond distances have lengthened while the C–N (−0.049 Å) bond distances have contracted in 6 (Table 1 and Fig. 5). These changes in bond distance support a two-electron oxidized diimine ligand bound to a Co^II^ center.

With the dehydrogenated ligand framework demonstrating the reversible storage of two electrons *via* the addition of outersphere oxidants, we next assessed whether a similar two-electron ligand oxidation process could be realized through substrate oxidative addition. Attempts to oxidatively add BuBr to 2 resulted in products consistent with one-electron reactivity, forming two new Co complexes proposed to be the neutral five-coordinate Co-butyl and Co-bromide products. Full identification and characterization of these products was not pursued further, but spectral data is provided in Fig. S25† for the interested reader.

Since 1 could be regenerated through addition of H_2_(g) to 2 (*vide supra*), we hypothesized that the addition of two hydride equivalents to 6 might, likewise, regenerate 1. Addition of 2.2 equivalents KBEt_3_H to 6 resulted in formation of some 1, but the major product was an as-yet-unidentified diamagnetic complex (Fig. S24†).

### Magnetic susceptibility and EPR spectroscopy

**Fig. 6:**
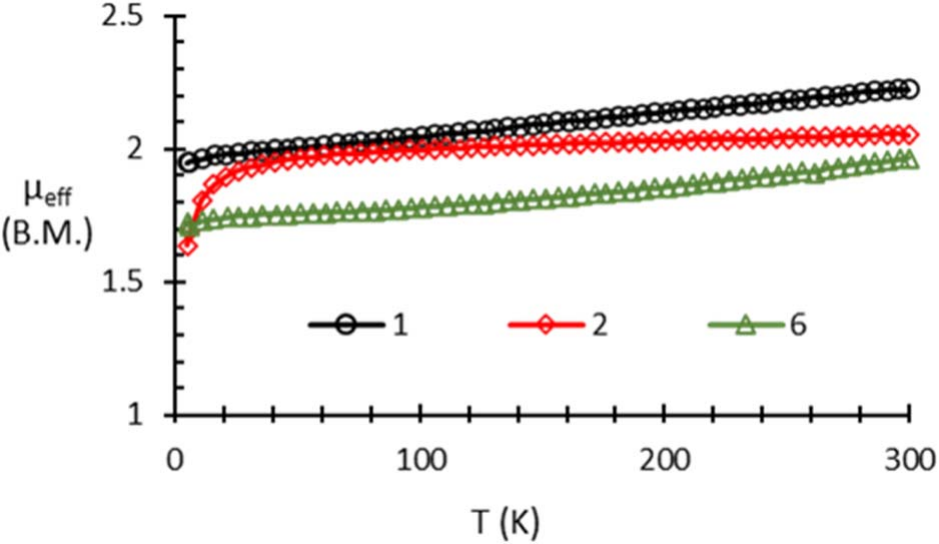
Solid-state SQUID magnetometry data (*μ*_eff_*vs. T*) for 1 (black circles), 2 (red diamonds), and 6 (green triangles) recorded at 1 T.

To further support the assigned electronic structures of 1, 2, 5 and 6, magnetic susceptibility measurements using a super-conducting quantum interface device (SQUID) magnetometer were performed (Fig. 6). The variable temperature magnetic susceptibility data for 1 shows *μ*_eff_ gradually increasing from *μ*_eff_ = 1.9 *μ*_B_ at 5 K to *μ*_eff_ = 2.22 *μ*_B_ at 300 K. The magnetic data for 2 shows a sharp increase in *μ*_eff_ from *μ*_eff_ = 1.64 *μ*_B_ at 5 K to *μ*_eff_ = 1.94 *μ*_B_ at 36 K and then a gradual increase to *μ*_eff_ = 2.05 *μ*_B_ at 300 K. Variable temperature magnetic susceptibility analysis of 5 showed diamagnetic behavior at all temperatures (Fig. S34 and S37†). The magnetic data for 6 shows a gradual increase from *μ*_eff_ = 1.72 *μ*_B_ at 5 K to *μ*_eff_ = 1.97 *μ*_B_ at 300 K. The room temperature *μ*_eff_ values of 1, 2 and 6 (2.22 *μ*_B_, 2.05 *μ*_B_, and 1.97 *μ*_B_, respectively) are consistent with the spin-only value expected for a compound with one unpaired electron (1.73 *μ*_B_) and with previously reported Co^II^*S* = 1/2 complexes in a similar coordination environment.^66,71,72^

**Fig. 7:**
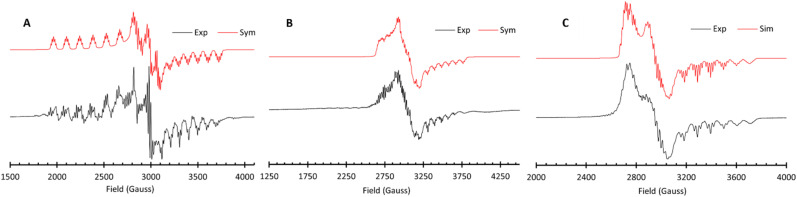
(A) X-band (9.4 GHz) EPR spectrum of a 2.5 mM frozen solution of 1 in THF at 30 K at a power of 30 dB. (B) X-band (9.4 GHz) EPR spectrum of a 2.5 mM frozen solution of 2 in THF at 30 K at a power of 20 dB. (C) X-band (9.4 GHz) EPR spectrum of a saturated frozen solution of 6 in THF at 30 K at a power of 30 dB.

EPR spectroscopy was also used to probe and compare the electronic and structural properties of 1, 2 and 6. The EPR spectrum of 1 in frozen THF (Fig. 7A) displays a rhombic signal with three separate *g* values (*g* = 2.72, 2.29 and 1.97) consistent with previously reported low-spin square planar Co^II^ complexes.^64,73,74^ Each portion of the spectrum features an 8-line splitting pattern owing to hyperfine coupling to the ^59^Co (*I* = 7/2) nucleus (*A*_Co_ = 541, 61 and 269 MHz), with additional superhyperfine coupling to the two ^14^N (*I* = 1) nuclei (*A*_N1_ = 47, 50, and 40 MHz and *A*_N2_ = 60, 48 and 50 MHz). The asymmetry of the nitrogen hyperfine tensors is consistent with the non-planar backbone orientation observed in the solid-state structure of 1 (Fig. 2), which likely disrupts Co–N interactions in the *xy* plane. The EPR spectrum of 2 (Fig. 7B) is also rhombic (*g* = 2.35, 2.19 and 1.94), but with notably less anisotropy than 1. Similar to 1, the signal is also split by ^59^Co (*A*_Co_ = 160, 95 and 248 MHz) and the ^14^N nuclei (*A*_N1_ = 50, 50 and 51 MHz and *A*_N2_ = 50, 50 and 50 MHz). The increased symmetry observed for the nitrogen hyperfine tensors and less anisotropic *g* values are consistent with the more planar backbone observed in the solid-state structure of 2 (Fig. 2).

The EPR spectrum of 6 in frozen THF displays a rhombic EPR signal with three separate *g* values (*g* = 2.43, 2.22 and 2.00) (Fig. 7C). The average *g* value of 2.22 is in support of a Co^II^ complex and is similar to the average *g* value obtained for 2 (*g*_avg_ = 2.16). Hyperfine coupling to the ^59^Co nucleus (*A*_Co_ = 32, 19, 291 MHz) is observed, along with superhyperfine coupling to the two nitrogen atoms (*A*_N1_ = 50, 49, 51 MHz, *A*_N2_ = 49, 47, 51 MHz).

The similarity of the *g* values and hyperfine tensors observed for 2 and 6 are in agreement with the assignment of 6 as a Co^II^ complex. The EPR is inconsistent with a Co^III^ complex containing a ligand-based radical, as the *g* value for a ligand-centered radical would be centered around *g* = 2.002 with much weaker hyperfine coupling to ^59^Co.

### Quantum chemical calculations

**Fig. 8:**
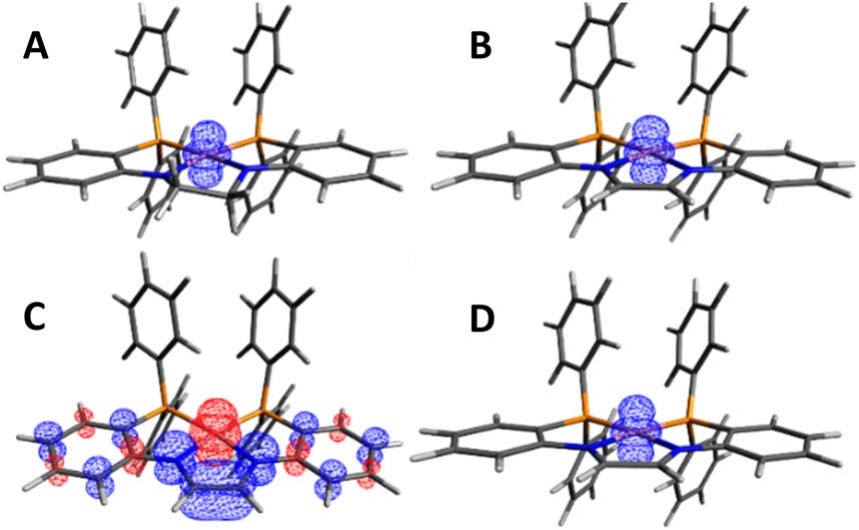
(A) Spin density plot of 1 (isovalue = 0.008). (B) Spin density plot of 2 (isovalue = 0.04). (C) Spin density plot of 5^0^_OS_ (isovalue = 0.004). (D) Spin density plot of 6 (isovalue = 0.008).

The electronic structures of 1, 2, 5, and 6 were investigated using density functional theory (DFT) calculations. Starting from crystallographically derived coordinates, geometries were optimized at the ωB97X-D3/def2-SVP level, followed by numerical frequency calculations. Calculations on the *S* = 1/2 complexes 1 and 2 revealed that the majority of the unpaired spin density is localized on the d_*z*^2^_ orbital of the Co^II^ center (Fig. 8A and B, respectively).

**Fig. 9:**
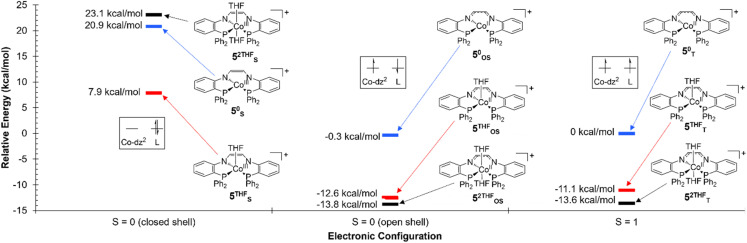
Relative energies of 5^0^ (blue), 5^THF^ (red), and 5^2THF^ (black) in the *S* = 0 (closed shell), *S* = 0 (open shell), and *S* = 1 electronic configurations. All energies are in reference to 5^0^ in the *S* = 1 electronic configuration.

A similar evaluation of the electronic structure of 5 was complicated by both the fluxionality of the complex in solution with respect to THF binding and the multiple possible spin configurations. Geometry optimizations and single-point numerical frequency calculations were conducted on the closed-shell *S* = 0 and open-shell *S* = 1 electronic configurations for 5, with either 0 (5^0^_S_, 5^0^_T_), one (5^THF^_S_, 5^THF^_T_), or two (5^2THF^_S_, 5^2THF^_T_) coordinated THF molecules. In all cases, the *S* = 1 electronic states 5^0^_T_/5^THF^_T_/5^2THF^_T_ were lower in energy than the closed shell *S* = 0 electronic configurations 5^0^_S_/5^THF^_S_/5^2THF^_S_ by more than 20 kcal mol^−1^ (Fig. 9). Since the prediction of a triplet state was inconsistent with the diamagnetic behavior of 5, an open-shell singlet configuration was probed using broken symmetry calculations. The open-shell singlet solutions 5^0^_OS_, 5^THF^_OS_, and 5^2THF^_OS_ were found to be slightly (less than 2 kcal mol^−1^) lower in energy than the corresponding triplet electronic configurations 5^0^_T_, 5^THF^_T_, and 5^2THF^_T_ (Fig. 9). 5^THF^_OS_ and 5^2THF^_OS_ were found to be similar in energy (within 2 kcal mol^−1^). The spin density plots for 5^0^_OS_, 5^THF^_OS_, and 5^2THF^_OS_ (Fig. S42–44†) do not differ significantly; therefore, the spin density plot of 5^0^_OS_ is displayed in Fig. 8C for simplicity. The spin density plot of 5^0^_OS_ shows unpaired electron density in a Co d_*z*^2^_ orbital with the electron density of the opposite sign delocalized throughout the ligand backbone and aryl linkers. The Loewdin population analysis shows significant electron density localized on the Co^II^ center (−1.05), nitrogen atoms (0.48), and C–C backbone (0.32). The spin density plot and Loewdin population analysis are in agreement with a singlet biradical electronic configuration for 5.

To aid in the assignment of 6 as an *S* = 1/2 Co^II^ diimine complex, geometry optimizations and single-point numerical frequency calculations were performed on 6 with an *S* = 1/2 and *S* = 3/2 electronic configuration. Optimization of 6 in an *S* = 1/2 electronic configuration resulted in good agreement with the solid-state structure (Table S8†) and the spin density plot (Fig. 8D) shows the majority of unpaired electron density localized on the Co^II^ center in a d_*z*^2^_ orbital. The *S* = 3/2 electronic configuration of 6 was in poor agreement with the solid-state structure of 6 (Table S8†) and was 16.3 kcal mol^−1^ higher in energy than the *S* = 1/2 electronic state. The above computations support the assignment of an *S* = 1/2 Co^II^ diimine complex as the well-isolated ground state of 6.

### Addition of PMe_3_ to 5

**Scheme 7:**
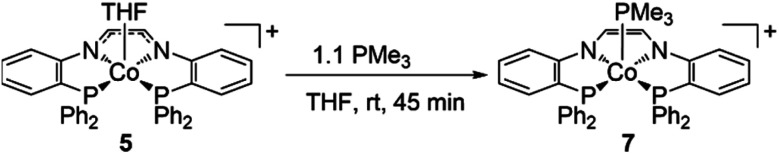
Synthesis of 7.

**Fig. 10:**
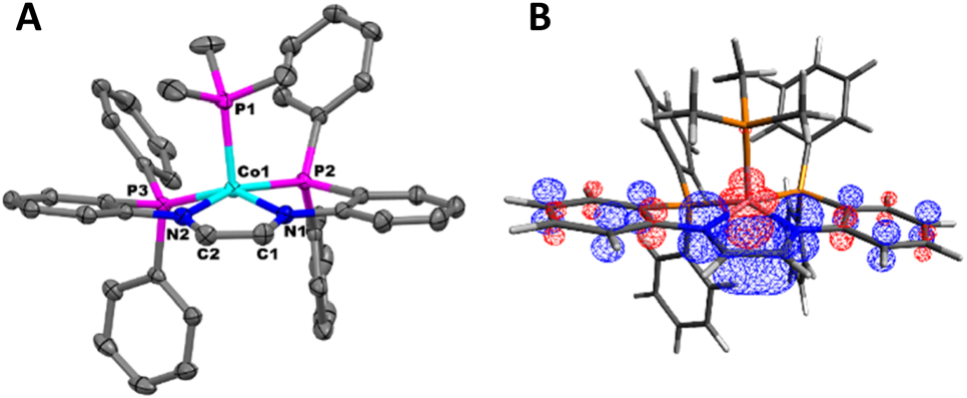
(A) Displacement ellipsoid (50%) representation of 7. All H atoms, solvate molecules, and PF_6_^−^ anions were omitted for clarity. (B) Spin density plot of 7_OS_ (isovalue = 0.004).

To examine the influence of ligand donor strength on the electronic structure and to simplify the fluxional coordination behavior of 5, the THF molecule bound to 5 was exchanged with a stronger donor ligand. Addition of PMe_3_ to 5 afforded 7, an analogue of 5 with a PMe_3_ molecule occupying an axial coordination site (Scheme 7). The ^1^H and ^31^P{^1^H} NMR spectra of 7 are consistent with a diamagnetic complex with a single bound PMe_3_ ligand, with ^31^P{^1^H} NMR chemical shifts at 52.29 and 8.60 ppm (Fig. S20 and S22†). Single crystals of 7 suitable for X-ray diffraction were grown from a C_6_D_6_/THF solution of 7 at room temperature (Fig. 10A). The average Co–N (1.8864(18) Å) and C–N (1.350(3) Å) bond distances of 7 lie between those found for 2 and 5, while the C–C bond distance of 7 (1.378(2) Å) is shorter than in both 2 and 5 (Table 1). Additionally, 7 displays a *τ*_5_ of 0.21, indicating a larger distortion away from ideal square pyramidal geometry compared to 5 (*τ*_5_ = 0.01) as the Co atom is pulled out of the P–N–N–P plane by the strongly donating PMe_3_ ligand.

Due to the diamagnetic properties of 7 the *S* = 0 closed shell (7_S_) and *S* = 0 open shell (7_OS_) electronic configurations were investigated computationally using DFT. The 7_OS_ electronic state was found to be 9 kcal mol^−1^ lower in energy than the 7_S_ electronic state (Fig. S39†). The spin density plot of 7_OS_ shows spin density in a Co d_*z*^2^_ orbital and spin density of the opposite sign delocalized throughout the ligand backbone (Fig. 10B).

Following experimental and computational analysis, a Co^II^/L˙^−^ electronic configuration is probable for 7. When comparing 7 to 2, the C–N bond distances are shortened and the Co–N bond distances are elongated, which is consistent with an oxidized ligand. Additionally, the average Co–P bond distance associated with the tetradentate ligand in 7 is similar to 2 and 5, which suggests a similar oxidation state between these three complexes (Table 1). DFT calculations also determined 7_OS_ to be lower in energy than the 7_S_ electronic configuration. Although we propose 7 to adopt an open-shell singlet electronic configuration, it is possible there is a resonance contribution from the Co^III^/L^2−^ electronic state, indicated by the smaller energy gap between 7_S_ and 7_OS_ compared to 5^THF^_S_ and 5^THF^_OS_.^42^ In either case, a stronger σ donor ligand was shown to reduce the energy gap between the open-shell and closed-shell singlet electronic configurations of the [(PNCHCHNP)CoL]^+^ complex (Fig. S39†). A similar phenomenon was observed with ligand-mediated spin-state changes in a cobalt–dipyrrin–bisphenol complex.^52^

## Conclusion

In conclusion, a new tetradentate Co^II^ complex (1) was synthesized and the ligand backbone dehydrogenated through hydrogen atom abstraction to afford a Co^II^ complex with a redox active ligand (2). Cyclic voltammetry experiments revealed reversible reductions for 1 and 2, and two reversible ligand-based oxidations for 2. Reduction of 1 and 2 with KC_8_ resulted in formation of Co^I^ species 3 and 4, supported by single crystal X-ray diffraction data. The singly oxidized (5) and doubly oxidized (6) analogues of 2 were isolated following treatment of 2 or 5 with FcPF_6_. A combination of spectroscopic techniques, magnetic susceptibility measurements, and DFT calculations determined 5 and 6 are Co^II^ complexes with either one- or two-electron oxidized ligands, respectively.

We hope the unconventional synthetic route reported here will inspire access to new redox-active ligand scaffolds. From our attempts to replicate post-metalation hydrogen atom abstraction with the Ni analogue of 1, it is clear that the identity of the coordinated transition metal plays an important role in dictating whether such synthetic methods are possible. The 270 mV more positive oxidation potential of 1-Ni leads to no reaction with TEMPO and a reversible equilibrium with 2,4,6-tri-*tert*-butylphenoxyl radical. At the same time, it is likely important that the oxidation potential of the metal complex is high enough to prevent direct oxidation of the metal center by the organic radical reagent. It is also worth noting that the structure and conjugation of the ligand backbone also plays an important role in dictating whether hydrogen atom abstraction is feasible as a method to generate redox-active ligands. For example, the pyrrole-based (PNP)Co complex reported by Tonzetich (Fig. 1) has a much more positive oxidation potential (0.3 V) than 1 or 1-Ni, but readily undergoes hydrogen atom abstraction when treated with *p*-benzoquinone,^54^ likely owing to the more acidic C–H bonds in the ligand backbone. Likewise, the bis(phosphine)amido (PNP)Co and (PNP)Ni complexes reported by Schneider (Fig. 1) have oxidation potentials of ∼100 mV more positive than 1 and 1-Ni but, in this case, both the Co and Ni complexes readily undergo irreversible hydrogen atom abstraction with 2,4,6-tri-*tert*-butyl-phenoxy radical.^55,56^ The latter comparison showcases the differences that ethylene backbone substituents (N *vs.* P; *N*-aryl *vs. N*-alkyl) can impart on the driving force for hydrogen atom abstraction reactions.

Future directions will assess the utility of the redox-active PNNP ligand to facilitate substrate activation and catalysis, with the ligand acting as an electron reservoir.

## Author contributions

C. M. T. supervised and acquired funding for the project. J. D. M. performed experiments and data analysis on complexes 1–7 and wrote the original draft of the manuscript. C. E. M. collected and refined data for the reported single-crystal structures. M. M. W. and K. L. performed the preliminary experiments and data analysis for the Ni complexes. C. M. T. and J. D. M. contributed to reviewing and editing the manuscript and all authors gave approval to the final version.

## Conflicts of interest

There are no conflicts to declare.

## Supplementary Material

SC-015-D4SC03364G-s001

SC-015-D4SC03364G-s002

## Data Availability

The data supporting this article have been included as part of the ESI.† Crystallographic data for 1–7 have been deposited at the Cambridge Crystallographic Data Center (CCDC) under CCDC 2354340–2354345 and 2372722.
