# Supplementary material for: Hydrogen atom abstraction as a synthetic route to a square planar CoII complex with a redox-active tetradentate PNNP ligand
Source: Chem Sci. 2024 Aug 30;15(37):15311–20. doi: 10.1039/d4sc03364g (PMC11362828; doi:10.1039/d4sc03364g)
Supplement: SC-015-D4SC03364G-s001 [file SC-015-D4SC03364G-s001.pdf]

Supplementary Information (SI) for Chemical Science.
This journal is © The Royal Society of Chemistry 2024

Hydrogen Atom Abstraction as a Synthetic route to a Square Planar Co^{II} Complex with a Redox-Active Tetradentate PNNP Ligand.

Justin D. Miller, Mitchell M. Walsh, Kyounghoon Lee, Curtis E. Moore, and Christine M. Thomas *

Department of Chemistry and Biochemistry, The Ohio State University, 100 W. 18th Ave, Columbus, OH 43210, USA. thomasc@chemistry.ohio-state.edu

Table of Contents

Experimental Details 2
- General Considerations 2
- Synthesis of ($PNCH_2CH_2NP$)Co (1). 3
- Synthesis of (PNCHCHNP)Co (2). 3
- Synthesis of [($PNCH_2CH_2NP$)Co][K(18-crown-6)] (3). 3
- Synthesis of [(PNCHCHNP)Co][K(18-crown-6)] (4). 4
- Synthesis of {[(PNCHCHNP)Co(THF)][PF_6]}•THF (5). 5
- Synthesis of [(PNCHCHNP)Co][PF_6]$_2$ (6). 5
- Synthesis of [(PNCHCHNP)Co(PMe_3)][PF_6] (7). 5
- Addition of H_2 to 2. 6
- H-atom abstraction from ($PNCH_2CH_2NP$)Ni (1-Ni). 6
- Reaction of 6 with $KBEt_3H$. 6
- Reaction of 2 with BuBr 6

NMR Spectroscopy 7

Figure S26. Cyclic voltammogram of ($PNCH_2CH_2NP$)Ni (1-Ni). 20

Crystal Structures 21

Crystal Structure Refinement Data 29

Magnetic Susceptibility Data 33

Moment vs Field Strength Graphs 34

Quantum Chemical Calculations 38
- Computational Details 38
- Spin Density Plots 39
- Comparison to X-ray structures 42
- XYZ coordinates of optimized structures 44

References 78

Experimental Details

General Considerations. Unless otherwise noted, all manipulations were carried out under an inert atmosphere using a nitrogen-filled glovebox or standard Schlenk techniques. Glassware was oven-dried before use. Solvents were degassed by sparging with ultra-high purity argon and dried via passage through columns of drying agents using a Glass Contours solvent purification system from Pure Process Technologies. Benzene-d_6 was degassed via repeated freeze-pump-thaw cycles and dried over 3 Å molecular sieves before use. Tetrahydrofuran-d_8 and methylene chloride-d_2 were dried over calcium hydride overnight then vacuum transferred into a sealed Schlenk tube and stored over 3 Å molecular sieves in a nitrogen filled glovebox. H_2[PNNP],[1] Co[N(SiMe$_3$)$_2$]$_2$(THF),[2] and 2,4,6-tri-tert-butylphenoxyl radical,[3] were prepared according to literature methods. 2,2,6,6-tetramethyl-1-piperidinyloxy (TEMPO) was purchased commercially then purified by sublimation and stored in a nitrogen filled glovebox. KC_8 was prepared by mixing 1.1 equiv of potassium metal with one equiv of graphite powder in a vial then heating to 100 ^oC with occasional stirring using a glass pipet until the solid mixture turned golden brown (about 10 minutes). $CoCl_2$ was dried at 120 ^oC in vacuo overnight. 18-crown-6 was dried at 60 ^oC in vacuo overnight. All other chemicals were purchased from commercial vendors and used without purification. NMR spectra were recorded at ambient temperature on a AVANCE NEO 400 MHz or Bruker AVANCE III HD Ascend 700 MHz NMR spectrometer. ^1H and ^{13}C NMR chemical shifts were referenced to residual solvent resonances and are reported in ppm. ^{31}P NMR chemical shifts (in ppm) were referenced to 85% H_3PO_4 (0 ppm) using an external standard. Elemental analysis (CHN) data were collected by either Midwest Microlab, Indianapolis, IN or at the Central Instrumentation Center, Department of Chemistry, University of Buffalo, Buffalo NY.

Cyclic Voltammetry

Cyclic voltammetry measurements were conducted in a nitrogen filled glovebox using a 0.1 M [^{n}Bu$_4$N][PF$_6$] electrolyte solution in THF using a CHI 620 E potentiostat (CH instruments Inc., Austin TX). A glassy carbon electrode was used for the working electrode, a platinum wire was used for the counter electrode, and a Ag/Ag$^+$ reference electrode was used with 0.01 M $AgNO_3$/0.1 M [^{n}Bu$_4$N][PF$_6$] as the reference solution.

Electron Paramagnetic Resonance

X-band (9.4 GHz) EPR spectra were collected at 30 K at either 20 dB or 30 dB using a modulation amplitude of 10 G with a Bruker EMX-plus spectrometer, and simulated using EasySpin for MATLAB.[4] All samples were prepared in THF solutions in a nitrogen-filled glovebox and immediately frozen in liquid nitrogen once removed from the glovebox.

Magnetic Measurements

Solid-state magnetic susceptibility measurements were obtaining using a Quantum Design MPMS3 SQUID magnetometer (NanoSystems Laboratory, The Ohio State University). The sample was prepared inside a nitrogen-filled glovebox and sealed in an inert atmosphere with eicosane wax inside of a gel capsule. A plastic straw was used to suspend the sample inside the SQUID magnetometer and magnetic susceptibility measurements were collected at 1 T. Sample purity was verified using magnetic moment vs field (M vs H) plots (Figures S33-S36).

Synthesis of (PNCH$_2$CH$_2$NP)Co (1). A 500 mL round bottom flask was charged with THF (200 mL), H$_2$[PNNP] (2.7785 g, 4.7828 mmol) and a stir bar, then placed in a liquid nitrogen-cooled cold well in the glovebox for 10 min. With stirring, a solution of Co[N(SiMe$_3$)$_2$]$_2$(THF) (2.1908 g, 4.8486 mmol) in THF (20 mL) was added dropwise over 20 minutes. The solution turned from colorless to black over the course of the addition. The reaction flask was removed from the cold well and allowed to warm to room temperature and stir for 2 hours. The solvent volume was reduced to 150 mL in vacuo then pentane was added (150 mL). The resulting suspension was filtered over a medium frit and the collected solids were washed with hexanes (3 x 50 mL) to afford a shiny olive-green solid. The solid was transferred to a pre-weighed 20 mL scintillation vial and dried in vacuo to afford **1** (2.7118 g, 87.7%) as an olive-green powder. **Alternative Synthesis.** H$_2$[PNNP] (0.0291 g, 0.0501 mmol) was dissolved in THF (4 mL) in a 20 mL scintillation vial, Co[N(SiMe$_3$)$_2$]$_2$(THF) (0.0227 g, 0.0502 mmol) was dissolved in THF (4 mL) in a 20 mL scintillation vial. Each vial was placed in the glovebox freezer (-35 ^{o}C) and after 20 minutes the Co[N(SiMe$_3$)$_2$]$_2$(THF) solution was added dropwise to the H$_2$[PNNP] solution resulting in a color change from colorless to black over the course of the addition. The solution was allowed to stir for two hours, then the solvent was removed in vacuo. The remaining solid was redissolved in THF (8 mL) and pentane (8 mL) was layered on top of the resulting solution. The crystallization was placed in the glovebox freezer (-35 ^{o}C), affording crystalline solid after three days. The mother liquor was decanted and the residual solvent was removed from the crystalline solid in vacuo, affording **1** as an olive green solid (0.0164 g, 51.3%). Single crystals suitable for X-ray diffraction were grown at room temperature from the diffusion of pentane vapor into a saturated benzene solution of **1**. ^{1}H NMR (400 MHz, C$_6$D$_6$): δ 145.87 (br s, C*H*$_2$C*H*$_2$, 4 H), 17.54 (br s, Ar-*H*, 2 H), 13.03 (br s, Ar-*H*, 2 H), 8.04 (t, *J* = 7.3 Hz, *para*-PPh$_2$, 4 H), 3.72 (d, *J* = 7.2 Hz, *ortho*-PPh$_2$, 8 H), 2.61 (br s, *meta*-PPh$_2$, 8 H), -2.66 (br s, Ar-*H*, 2 H), -16.46 (br s, Ar-*H*, 2 H). Elemental analysis calculated for C$_{38}$H$_{32}$CoN$_2$P$_2$: 71.59% C, 5.06% H, 4.39% N. Found: 71.03% C, 5.16% H, 4.32% N.

Synthesis of (PNCHCHNP)Co (2). To a 500 mL Strauss flask, a stir bar, (2,2,6,6-tetramethylpiperidin-1-yl)oxyl (TEMPO) (0.8405 g, 5.379 mmol) and **1** (0.6722 g, 1.054 mmol) were added, then the two solids were dissolved in THF (270 mL). The headspace was removed, then the Strauss flask was removed from the glovebox and heated to 55 ^{o}C in an oil bath with stirring. After two days, the flask was removed from the oil bath and allowed to cool to room temperature. The flask was brought into the glovebox and the solvent volume was reduced to 100 mL in vacuo, then the solution was split between two 150 mL jars. Pentane was layered on top of the reaction solution in each jar to achieve a 1:1 ratio of THF:pentane and placed in the glovebox freezer (-35 ^{o}C). After 3 days, the resulting crystalline solids were collected via filtration using a medium frit and the resulting red solid was washed with hexanes (3 x 50 mL). The solid was collected and dried in vacuo to yield **2** as a red powder (0.4866 g, 72.6%). Single crystals suitable for X-ray analysis were obtained from the reaction solution. ^{1}H NMR (400 MHz, C$_6$D$_6$): δ 39.41 (br s, Ar-*H*, 2 H), 29.26 (br s, Ar-*H*, 2 H), 4.88 (br s, PPh$_2$, 4 H), 3.08 (br s, PPh$_2$, 8 H), -1.97 (br s, PPh$_2$, 8 H), -41.91 (br s, Ar-*H*, 2 H), -47.68 (br s, Ar-*H*, 2 H), -92.84 (br s, NC*H*C*H*N, 2H). Elemental analysis calculated for C$_{38}$H$_{30}$CoN$_2$P$_2$: 71.81% C, 4.76% H, 4.41% N. Found: 71.83% C, 4.50% H, 4.21% N.

Synthesis of [(PNCH$_2$CH$_2$NP)Co][K(18-crown-6)] (3). KC$_8$ (0.0410 g, 0.303 mmol) and a stir bar were added to a 20 mL scintillation vial. In a separate 20 mL scintillation vial, **1** (0.0977 g, 0.153 mmol) was dissolved in THF (18 mL) then added to the vial containing

KC_8 with stirring, resulting in a color change from brown to green within 5 minutes. After 10 minutes, 18-crown-6 (0.0410 g, 0.155 mmol) was dissolved in THF (2 mL) then added to the reaction mixture with stirring. After two hours the reaction mixture was filtered through Celite and an additional 10 mL THF was used to extract any remaining solid. The solvent was removed from the filtrate in vacuo. The resulting shiny black residue was redissolved in benzene (10 mL), then the solution was frozen and the volatile components were removed via lyophilization to afford **3** as a black powder (0.1381 g, 95.8%). Single crystals suitable for X-ray diffraction were grown via the diffusion of pentane vapor into a saturated benzene solution of **3**. *Note: While only one stoichiometric equivalent of KC_8 should be required for the reduction of **1** to **3**, we report a reliable procedure that uses an excess (2 equiv) of KC_8 as this eliminates reproducibility concerns related to batch-to-batch variability of KC_8 purity, which is often difficult to assess.* ^{1}H NMR (700 MHz, THF-*d$_8$*): δ 7.35 (m, *ortho*-PPh_2, 8H), 6.93 (t, *J* = 7.3 Hz, *para*-PPh_2, 4H), 6.87 (m, Ar-*H*, 2H), 6.81 (t, *J* = 7.5 Hz, *meta*-PPh_2, 8H), 6.72 (t, *J* = 6.8 Hz, Ar-*H*, 2H), 6.04 (d, *J* = 8.0 Hz, Ar-*H*, 2H), 5.78 (t, *J* = 7.0 Hz, Ar-*H*, 2H), 3.32 (s, NCH_2CH_2N, 4H), 3.18 (br s, 18-crown-6, 24H). ^{31}P{^{1}H} NMR (162 MHz, THF): δ 59.0 (s). ^{13}C{^{1}H} NMR (176 MHz, THF-*d$_8$*) δ 165.91 (m, *ipso*), 138.49 (m, *ipso*-PPh_2), 133.09 (m, *ortho*-PPh_2), 131.47 (s, Ar-*C*), 130.03 (s, Ar-*C*), 127.35 (m, *ipso*), 126.97 (s, *meta*-PPh_2), 126.43 (s, *para*-PPh_2), 107.83 (s, Ar-*C*), 107.15 (m, Ar-*C*), 70.55 (s, 18-crown-6), 53.95 (s, NCH_2CH_2N). Elemental analysis calculated for $C_{50}H_{56}CoN_2P_2O_6K$: 63.82% C, 6.00% H, 2.98% N. Found: 63.82% C, 5.96% H, 3.02% N.

Synthesis of [(PNCHCHNP)Co][K(18-crown-6)] (4). KC_8 (0.0375 g, 0.277 mmol) and a stir bar were added to a 20 mL scintillation vial. In a separate 20 mL scintillation vial, **2** (0.0870 g, 0.137 mmol) was dissolved in THF (15 mL) then added to the vial containing KC_8 with stirring, resulting in a color change from red-orange to brown within 5 minutes. After 10 minutes, 18-crown-6 (0.0370 g, 0.140 mmol) was dissolved in THF (3 mL) then added to the reaction mixture with stirring. After two hours, the reaction mixture was filtered through Celite and an additional 5 mL THF was used to extract any residual solid. The solvent was removed from the filtrate in vacuo. The resulting shiny black residue was redissolved in benzene (5 mL), then the solution was frozen and the volatile components were removed in vacuo via lyophilization to afford **4** as a black powder (0.1142 g, 88.9%). Single crystals suitable for X-ray diffraction were grown from the diffusion of pentane vapor into a saturated benzene solution of **4**. *Note: While only one stoichiometric equivalent of KC_8 should be required for the reduction of **2** to **4**, we report a reliable procedure that uses an excess (2 equiv) of KC_8 as this eliminates reproducibility concerns related to batch-to-batch variability of KC_8 purity, which is often difficult to assess.* ^{1}H NMR (400 MHz, C_6D_6): δ 7.68 (m, *ortho*-PPh_2, 8H), 7.43 (m, Ar-*H*, 2H) 7.19 (t, *J* = 7.4 Hz, Ar-*H*, 2H), 6.99 (t, *J* = 7.2 Hz, *para*-PPh_2, 4H), 6.94 (d, *J* = 8.0 Hz, Ar-*H*, 2H), 6.85 (t, *J* = 7.5 Hz, *meta*-PPh_2, 8H), 6.73 (s, NC*H*C*H*CN, 2H), 6.46 (t, *J* = 7.1 Hz, Ar-*H*, 2H), 2.75 (br s, 18-crown-6, 24H). ^{31}P{^{1}H} NMR (162 MHz, C_6D_6): δ 59.08 (s). ^{13}C{^{1}H} NMR (176 MHz, C_6D_6): δ 161.78 (m, PPh_2 *ipso*), 137.98 (m, *ipso*), 133.12 (s, *ortho*-PPh_2), 131.78 (s, Ar-*C*), 130.42 (s, Ar-*C*), 128.35 (s, *ipso*), 127.35 (s, *meta*-PPh_2), 127.18 (s, *para*-PPh_2), 119.00 (s, N*C*H*C*HN), 112.87 (s, Ar-*C*), 108.93 (s, Ar-*C*), 69.84 (s, 18-crown-6). Repeated attempts to collect elemental analysis data for this compound were unsuccessful. Elemental analysis calculated for $C_{50}H_{54}CoN_2P_2O_6K$: 63.96% C, 5.80% H, 2.98% N. Found: 61.32% C, 5.59% H, 2.93% N. Due to the air sensitivity of this compound, the low carbon content could be due to oxidation during transport or handling of the material prior to analysis. For example, oxidation of the phosphine ligands would lead to

$C_{50}H_{54}CoN_2P_2O_8K$ (61.85% C, 5.61% H, 2.89% N) and these values align well with the obtained experimental data.

Synthesis of {[(PNCHCHNP)Co(THF)][PF_6]}•THF (5). $FcPF_6$ (0.0734 g, 0.222 mmol), **2** (0.1435 g, 0.2257 mmol), and a stir bar were added to a 20 mL scintillation vial, then THF (20 mL) was added with stirring, resulting in a dark purple solution within 5 minutes. After 4 h, the reaction solution was added dropwise to two vials containing pentane (10 mL each) that were then placed in the freezer (-35 °C) for 30 min. The solids were collected from the resulting suspension via filtration, followed by washing with benzene (3 x 15 mL) and hexanes (3 x 15 mL). The remaining solids were collected and dried in vacuo to afford **5** as a dark purple solid (0.1763 g, 85.4%). Single crystals suitable for X-ray diffraction were grown via diffusion of Et_2O vapor into a concentrated THF solution of **5**. ^{1}H NMR (400 MHz, CD_2Cl_2): δ 11.28 (s, NC*H*C*H*N, 2H), 9.39 (d, Ar-*H*, *J* = 8.1 Hz, 2H), 8.61 (t, Ar-*H*, *J* = 7.5 Hz, 2H), 7.73 (br s, Ar-*H*, 2H), 7.50 (t, Ar-*H*, *J* = 7.8 Hz, 2H), δ 7.36 (t, *para*-PPh_2, *J* = 7.5 Hz, 4H), 7.03 (t, *J* = 7.7 Hz, *meta*-PPh_2, 8H), 6.62 (br s, *ortho*-PPh_2, 8H), 3.61 (m, THF, 8H), 1.77 (m, THF, 8H). ^{31}P{^{1}H} NMR (162 MHz, CD_2Cl_2): δ 8.74 (br, s, *P*Ph_2, 2 P), 143.17 (sept, *P*F_6, *J* = 703 MHz, 1 P). ^{13}C{^{1}H} NMR (176 MHz, CD_2Cl_2) δ 168.29 (s, *ipso*), 149.79 (s), 138.32 (s), 137.18 (s), 132.77 (s), 131.32 (s), 130.30 (s, *ipso*), 129.21 (s), 128.57 (s, *ipso*), 127.30 (s), 118.01 (s), 69.69 (s, THF), 26.87 (s, THF). Elemental analysis calculated for $C_{46}H_{46}CoN_2P_3F_6O_2$: 59.75% C, 5.01% H, 3.03% N. Found: 59.05% C, 5.07% H, 3.09% N.

Synthesis of [(PNCHCHNP)Co][PF_6]$_2$ (6). $FcPF_6$ (0.0606 g, 0.183 mmol) and **5** (0.1723 g, 0.1814 mmol) were added to a 20 mL scintillation vial, then THF (18 mL) was added with stirring resulting in a dark green solution within 5 minutes. After 3 h, the reaction solution was split between two vials and pentane (10 mL each) was layered on top and the solutions were placed in a freezer (-35 °C). After three hours, the resulting green crystalline solids were collected via filtration and washed with benzene (3 x 6 mL) and hexanes (3 x 6 mL). The remaining solids were collected and dried in vacuo to yield **6** as an army-green powder (0.1420 g, 84.6 %). Single crystals suitable for X-ray analysis were grown by the diffusion of Et_2O vapor into a saturated THF solution of **6** at room temperature. ^{1}H NMR (400 MHz, THF-d_8): δ 14.10 (br s), 8.95 (br s), 7.40 (br s), 7.00 (br s), 5.40 (br s). Elemental analysis calculated for $C_{38}H_{30}CoN_2P_4F_{12}$: 49.32% C, 3.27% H, 3.03% N. Found: 49.31% C, 3.98% H, 3.01% N.

Synthesis of [(PNCHCHNP)Co(PMe_3)][PF_6] (7). Complex **5** (0.0805 g, 0.0870 mmol) was added to a 20 mL scintillation vial, dissolved in THF (10 mL) with stirring, then PMe_3 (9.9 μL, 0.096 mmol) was added via glass syringe resulting in a color change from dark purple to dark red within 5 minutes. After 30 min, the solvent volume was reduced to 5 mL, pentane (15 mL) was added, and the vial was placed in the freezer (-35 °C). After three hours, the resulting red crystalline solids were collected via filtration, washed with benzene (3 x 5 mL), then hexanes (3 x 5 mL). The remaining solids were collected and dried in vacuo to yield **7** as a red powder (0.0746 g, 95.2%). Single crystals suitable for X-ray analysis were grown from a C_6D_6 / THF solution at room temperature. ^{1}H NMR (400 MHz, CD_2Cl_2): δ 8.65 (d, *J* = 8.2 Hz, 2H), 8.11 (m, 2H), 7.60 (t, *J* = 7.5 Hz, 2H), 7.55 (m, PPh_2, 4H), 7.46 (m, 2H), 7.37 (m, PPh_2, 4H), 7.29 (m, PPh_2, 4H), 7.17 (t, *J* = 7.5 Hz, 2H), 6.92 (t, *J* = 7.8 Hz, PPh_2, 4H), 6.84 (m, PPh_2, 4H), 0.44 (d, *J* = 9.8 Hz, PMe_3, 9H). ^{31}P{^{1}H} NMR (162 MHz, CD_2Cl_2): δ 52.29 (br, s, *P*Ph_2, 2P), 8.60 (br, s, *P*Me_3, 1P), -143.95 (sept, *P*F_6, *J* = 710 Hz, 1P). ^{13}C{^{1}H} NMR (100 MHz, CD_2Cl_2) δ 158.72 (m, *ipso*), 136.93 (d, *J* = 1.8 Hz), 134.46 (d, *J* = 2.9 Hz), 133.79 (t, *J* = 4.9 Hz), 133.25 (d, *J* = 3.0 Hz), 132.68 (m,

ipso), 132.24 (s), 132.07 (t, J = 5.8 Hz), 131.70 (m, *ipso*), 130.85 (s), 129.64 (t, J = 5.0 Hz), 128.99 (t, J = 5.2 Hz), 128.33 (m, *ipso*), 127.38 (m), 116.18 (m), 13.66 (d, J = 23.5 Hz, PMe_3). Elemental analysis calculated for $C_{41}H_{39}CoF_6N_2P_2$: 57.49% C, 4.59% H, 3.27% N. Found: 57.49% C, 5.05% H, 3.09% N.

Addition of H_2 to 2. Complex **2** (0.0042 g, 0.0066 mmol) was dissolved in C_6D_6 (1 mL) then added to a J. Young tube in a nitrogen filled glovebox. The J. Young tube was then removed from the glovebox and two freeze-pump-thaw cycles were performed. The J. Young tube was then submerged in a dry ice/isopropanol cold bath. After 10 minutes, H_2 was added, the Teflon valve on the J. Young tube was closed, and the solution was allowed to warm to room temperature. After 4 h, the sample was analyzed using ^{1}H NMR spectroscopy (Figure S19).

H-atom abstraction from ($PNCH_2CH_2NP$)Ni (1-Ni). ($PNCH_2CH_2NP$)Ni (**1-Ni**) (0.0151 g, 0.0237 mmol) was added to a 20 mL scintillation vial and dissolved in THF (3 mL), then a THF solution (3 mL) of 2,4,6-tri-tert-butylphenoxyl radical (0.0156 g, 0.0597 mmol) was added with stirring. After stirring overnight, an aliquot was taken, dried in vacuo, then redissolved in C_6D_6 for NMR analysis (Figure S23, top). On the same day, additional 2,4,6-tri-tert-butylphenoxyl radical (0.0153 g, 0.0585 mmol) was added and the reaction was allowed to stir. After 2 days an aliquot of the reaction solution was used for $^{31}P\{^1H\}$ NMR analysis (Figure S23, middle). The remaining reaction solution was reduced to 3 mL in vacuo, then pentane (15 mL) was added. After 20 min the resulting suspension was filtered, the residual solid was washed with hexanes (3 mL), then extracted into THF. Solvent was removed from the THF extract in vacuo then the remaining solid was used for NMR analysis (Figure S23, bottom). ***Discussion:*** The hydrogen atom abstraction product, (PNCHCHNP)Ni (**2-Ni**), could not be isolated without the presence of the saturated **1-Ni** complex or other impurities. An equilibrium likely exists between **1-Ni** and **2-Ni**. As the concentration of the hydrogen atom abstractor increases the equilibrium favors the dehydrogenated compound **2-Ni**, but when this hydrogen atom abstractor is removed the equilibrium shifts back to regenerate the unsaturated analogue **1-Ni**.

Reaction of 6 with $KBEt_3H$. Complex **6** (0.0212 g, 0.0229 mmol) was added to a 20 mL scintillation vial and dissolved in THF (4 mL), then a THF solution (2.0 M) of $KBEt_3H$ was added via glass syringe (50.0 µL, 0.050 mmol). The solution was stirred for 3 h then an aliquot (1 mL) was collected, and the volatiles were removed from it in vacuo. The residual solid was dissolved in C_6D_6 for NMR analysis shown in Figure S24.

Reaction of 2 with BuBr. Complex **2** (0.0204 g, 0.0321 mmol) was added to a 20 mL scintillation vial and dissolved in THF (5 mL), then BuBr (3.5 µL, 0.0324 mmol) was added via glass syringe. After stirring for 22 h an aliquot (1 mL) of the reaction solution was collected and the volatiles were removed from it in vacuo. The resulting solid was dissolved in C_6D_6 for NMR analysis shown in Figure S25 (top). The volatiles were removed from the remaining reaction solution, then the resulting solid was dissolved in C_6H_6 (3 mL), frozen and lyophilized. The lyophilized solid was washed with hexanes (20 mL), Et_2O (15 mL), then the remaining solid was extracted into PhF (5 mL). The volatiles were then removed from the hexanes, Et_2O, and PhF solutions and the remaining solids for each were dissolved in C_6D_6 for NMR analysis (Figure S25, middle for hexanes and Figure S25, bottom for PhF).

NMR Spectroscopy

Figure S1. ^{1}H NMR spectrum of **1** (400 MHz, C_6D_6). # denotes residual C_6D_5H.

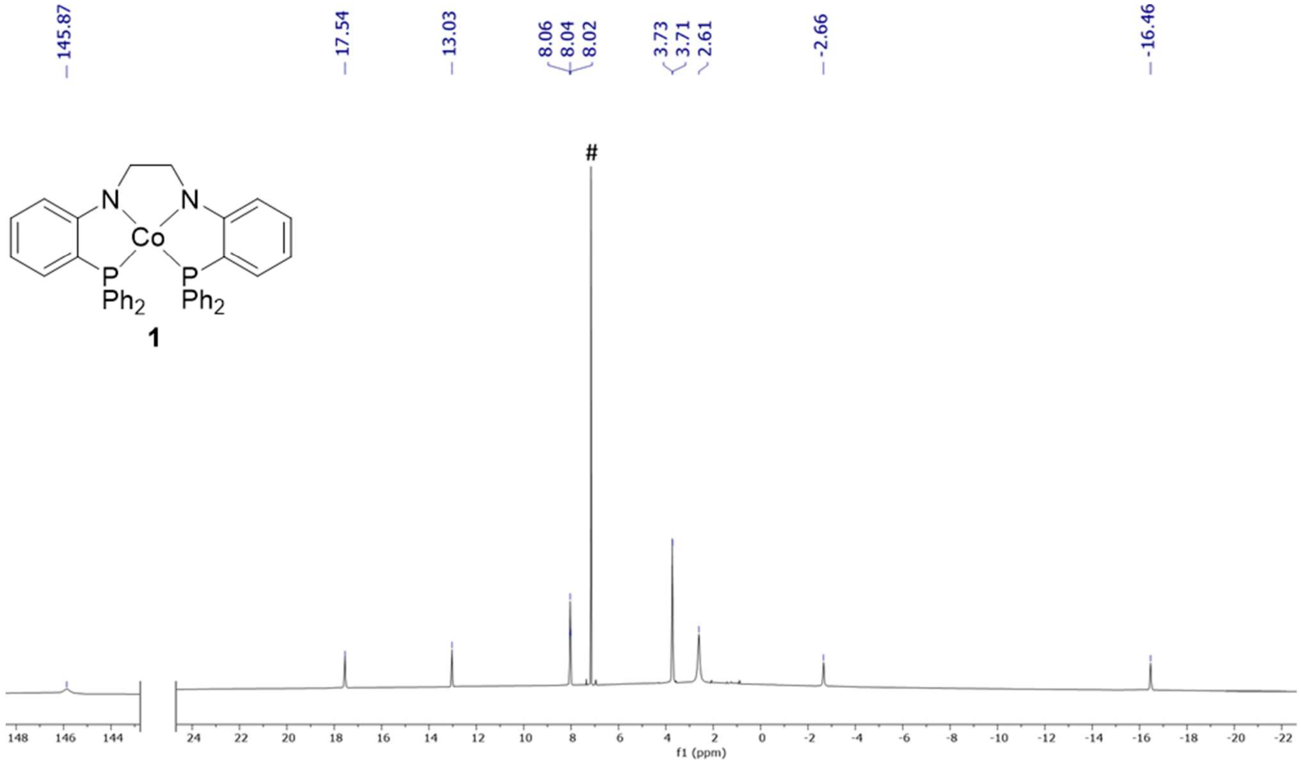

Figure S2. ^{1}H NMR spectrum of **2** (400 MHz, C_6D_6). Stars denote residual solvent impurities (C_6D_5H, THF and hexanes)

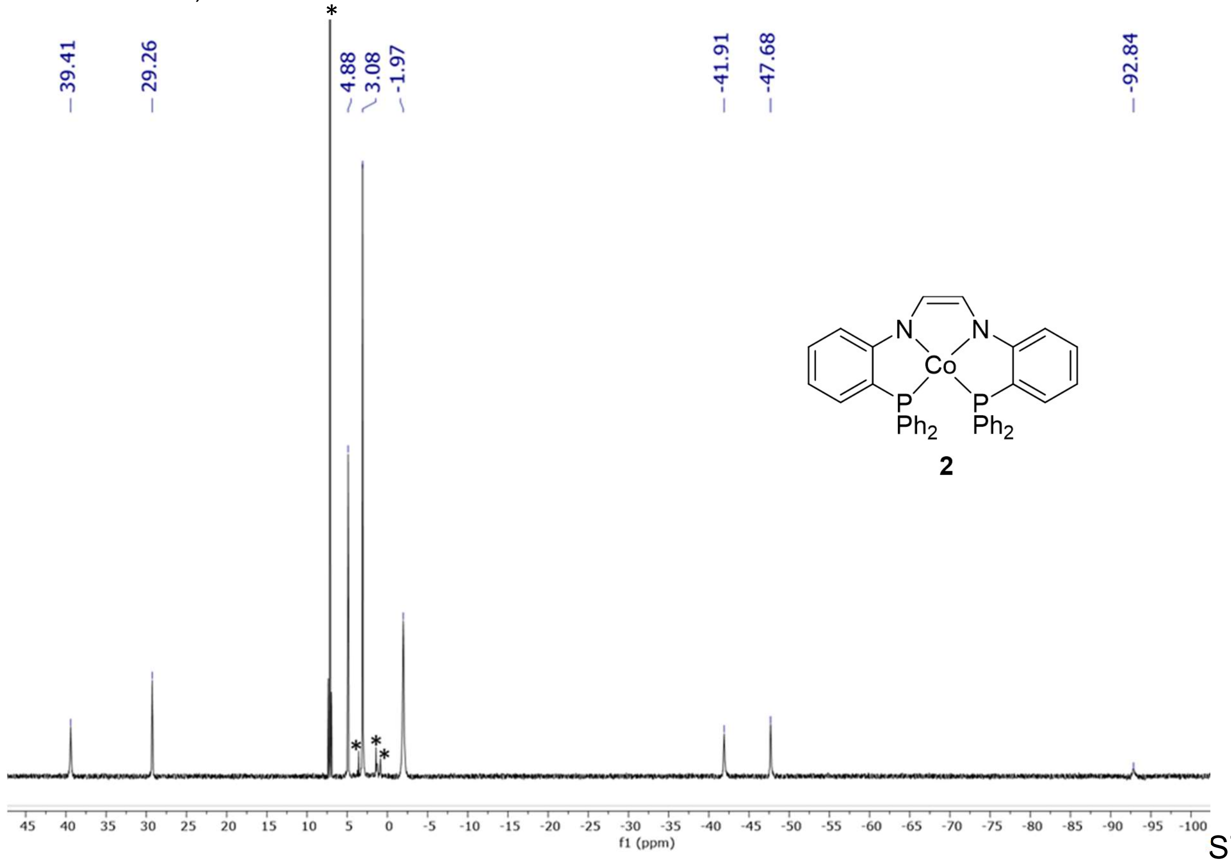

Figure S3. ^{1}H NMR spectrum of **2** (400 MHz, C_6D_6) synthesized from TEMPO (top) and 2,4,6-tri-tert-butylphenoxyl radical (bottom).

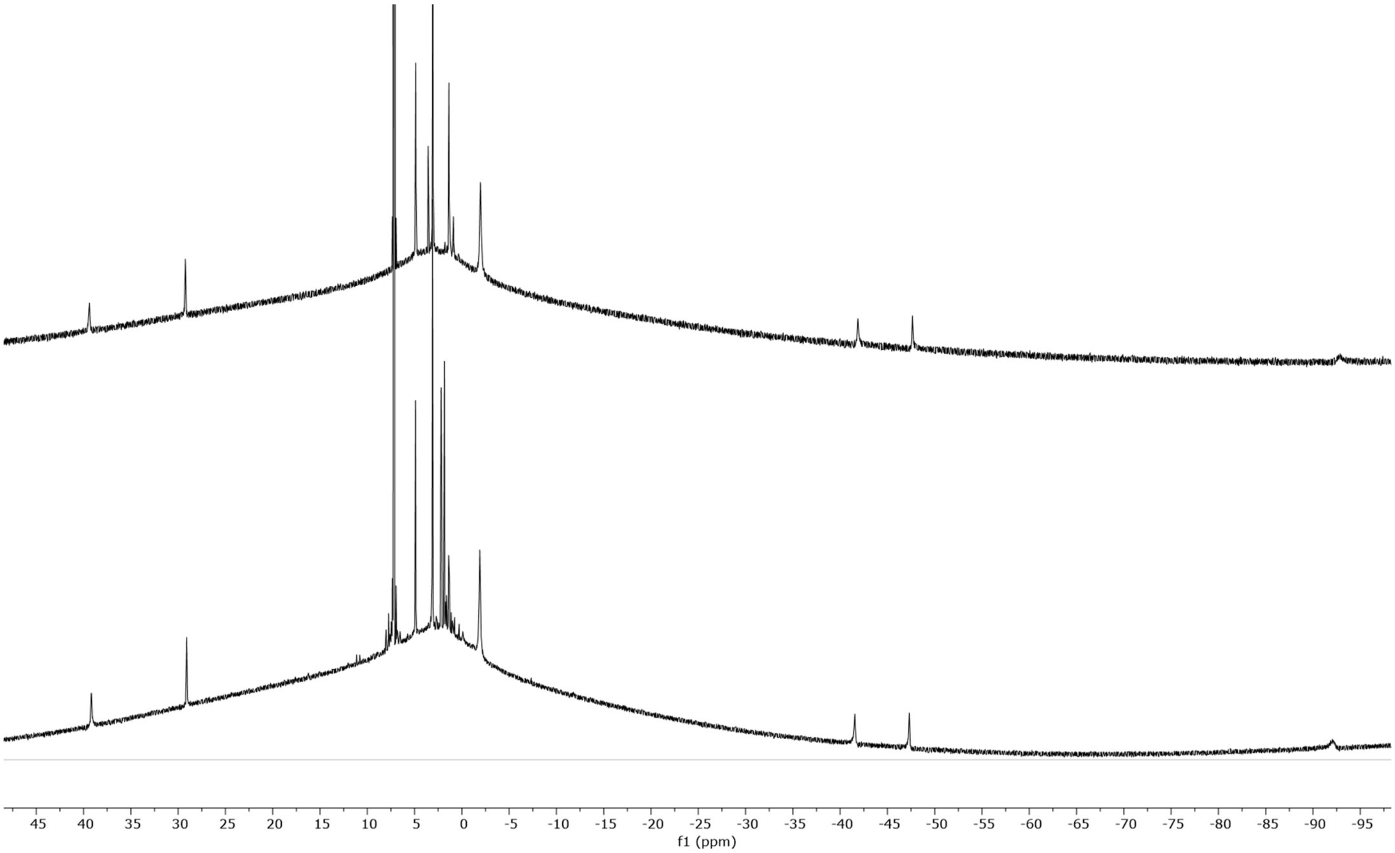

Figure S4. ^{1}H NMR spectrum of **3** (700 MHz, THF-d_8). Residual THF is denoted with a # and residual benzene is denoted with a *.

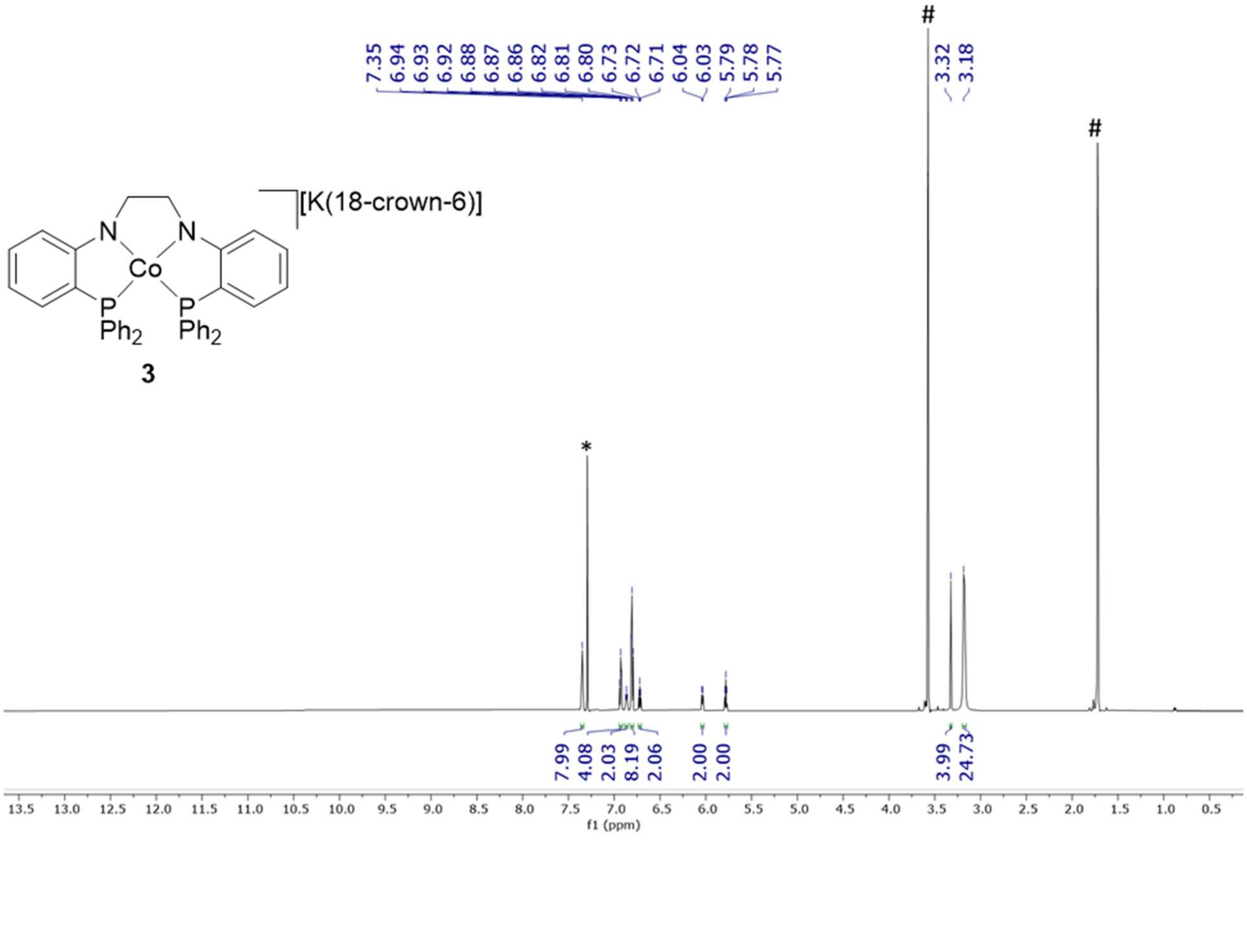

Figure S5. $^{13}C\{^{1}H\}$ NMR spectrum of **3** (176 MHz, THF-d_8). Residual THF is denoted with a # and benzene is denoted with a *.

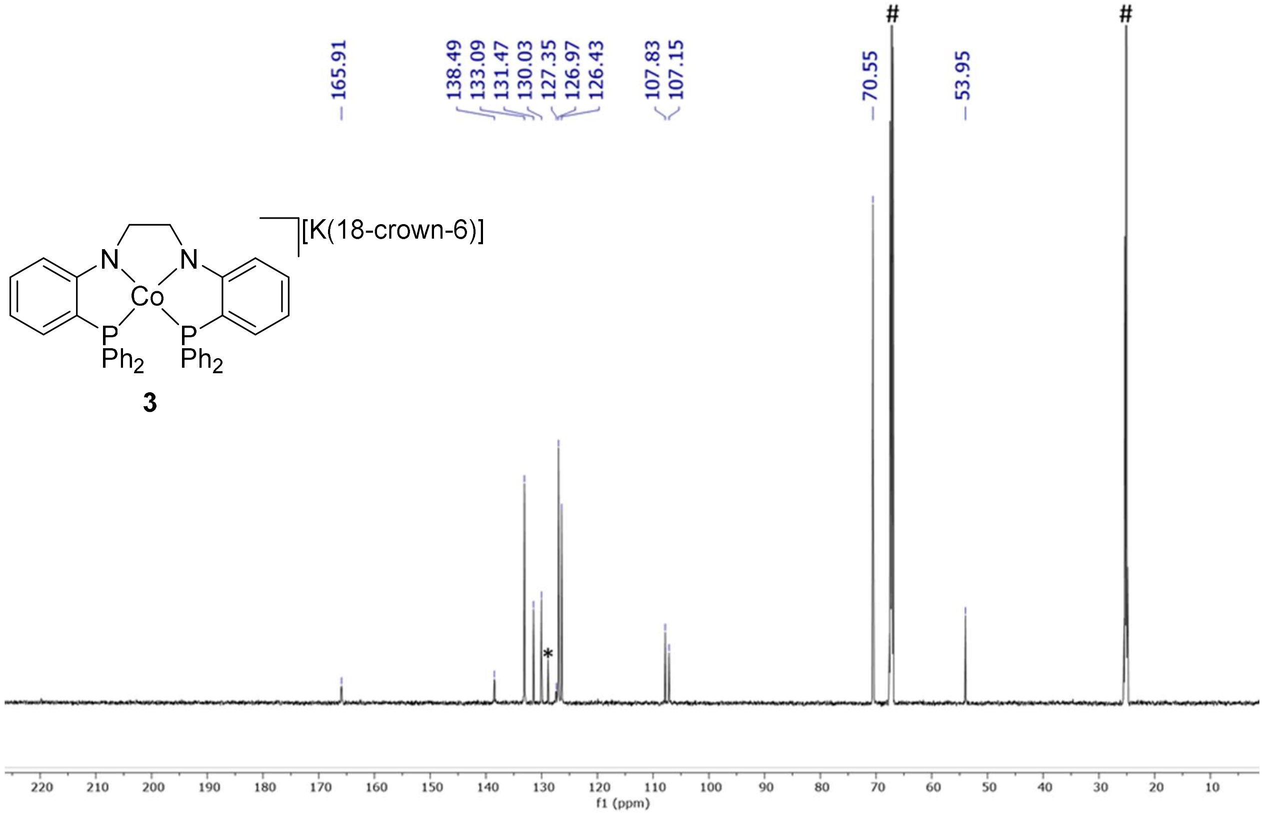

Figure S6. $^{31}P\{^{1}H\}$ NMR of **3** (162 MHz in THF).

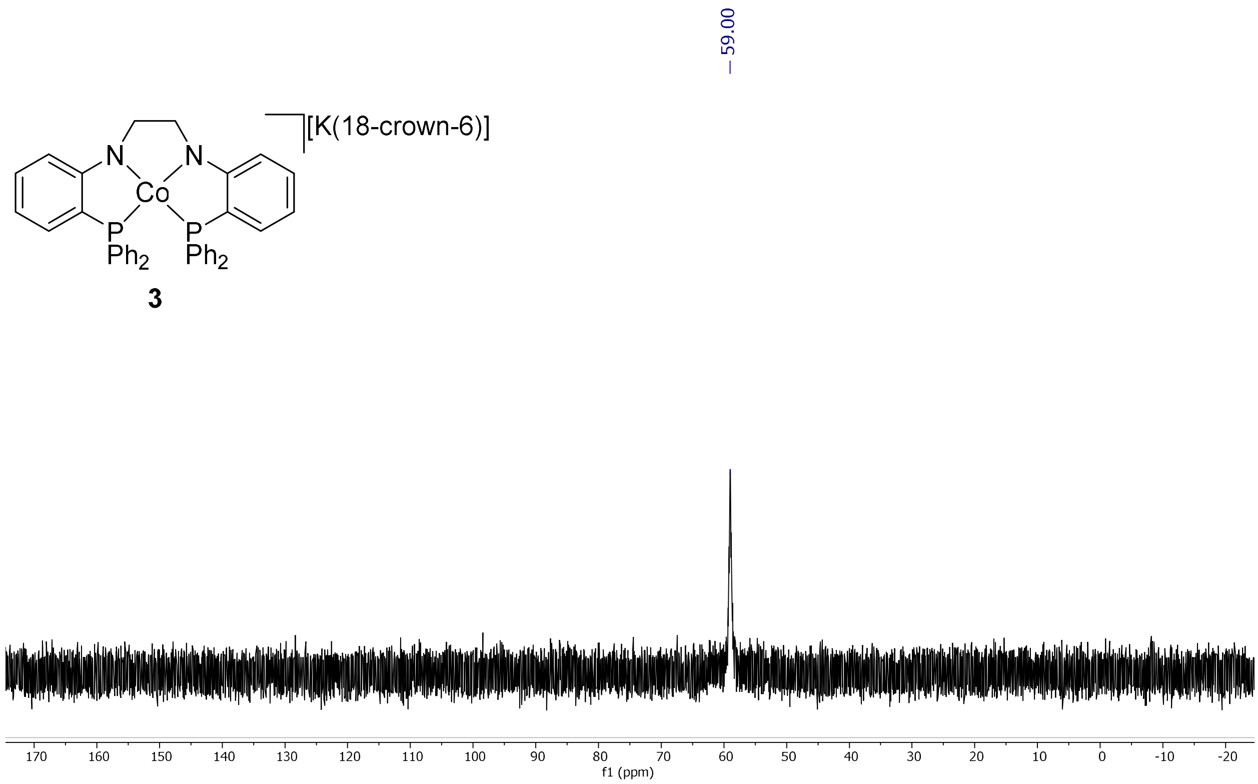

Figure S7. ^{13}C-^{1}H HSQC spectrum of **3** (700 MHz, THF-d_8) of. Cross peaks assigned to THF and benzene are labeled.

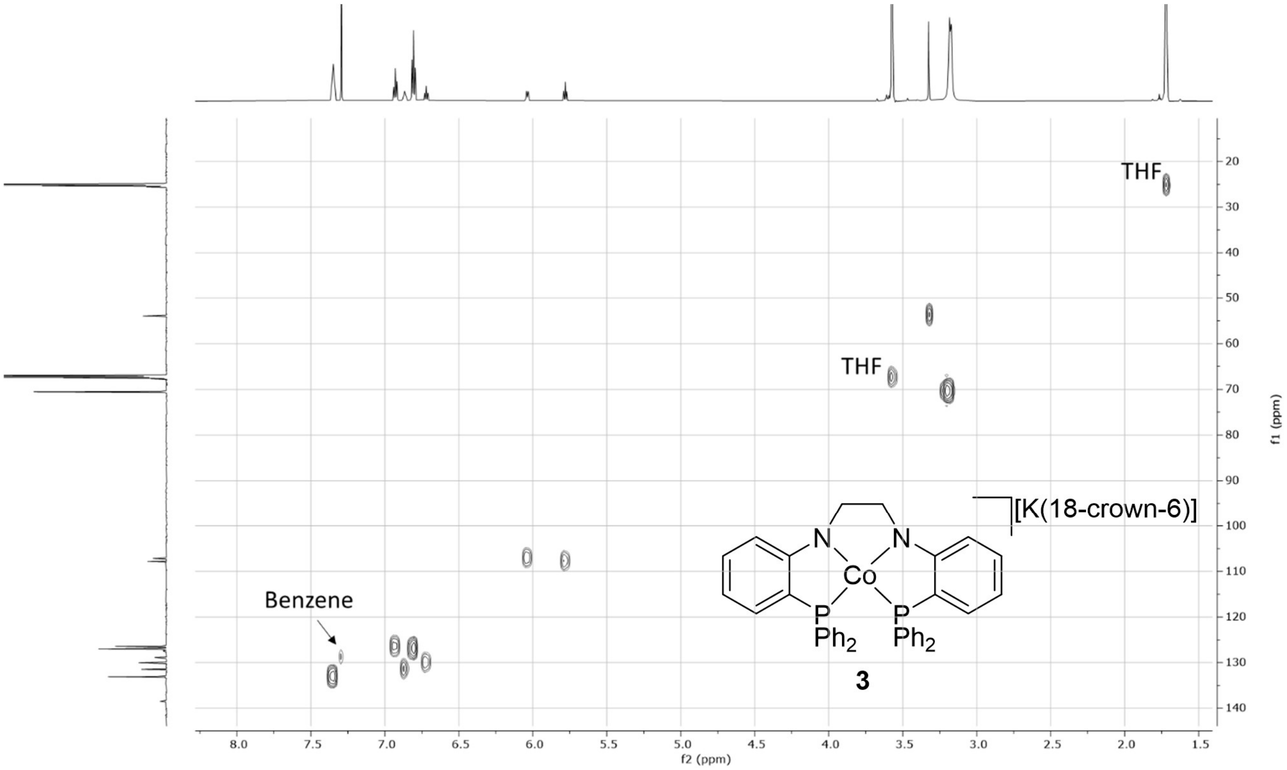

Figure S8. ^{13}C-^{1}H HMBC spectrum of **3** (700 MHz, THF-d_8). Cross peaks assigned to THF are labeled.

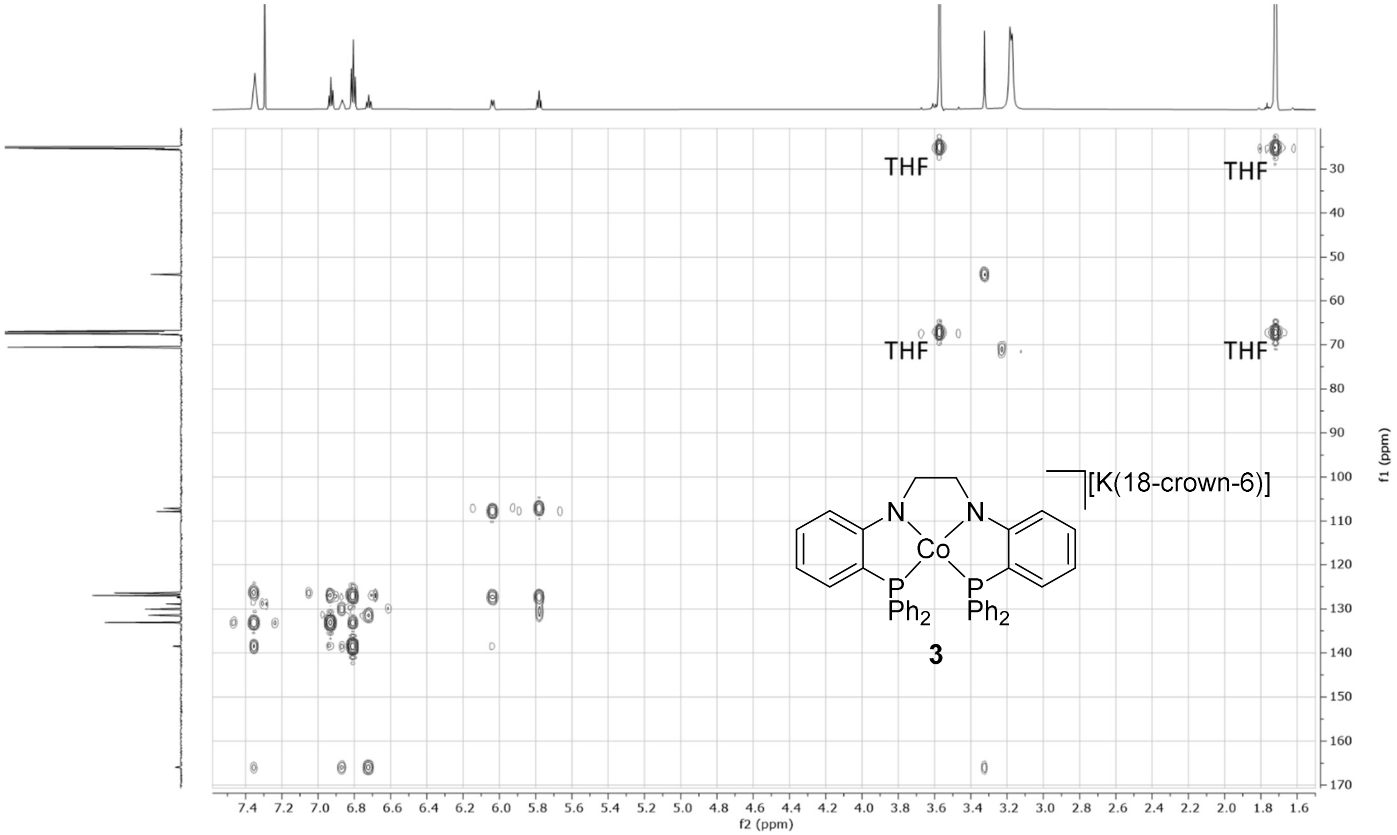

Figure S9. ^{1}H NMR spectrum of **4** (400 MHz, C_6D_6). Residual C_6D_5H is denoted with a #.

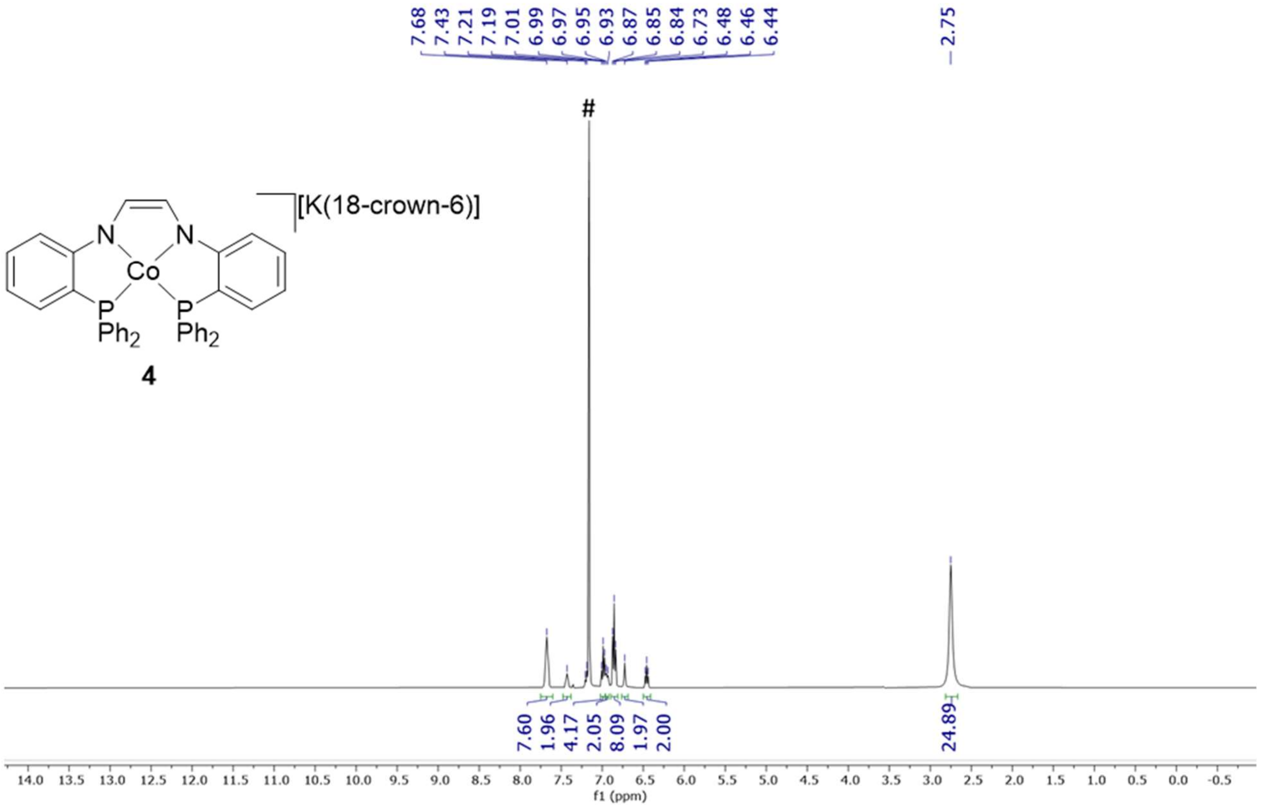

Figure S10. $^{13}C\{^{1}H\}$ NMR spectrum of **4** (176 MHz, C_6D_6). An inset was added to show the region around the residual C_6D_6. C_6D_6 is denoted with a # and residual C_6H_6 is denoted with a *.

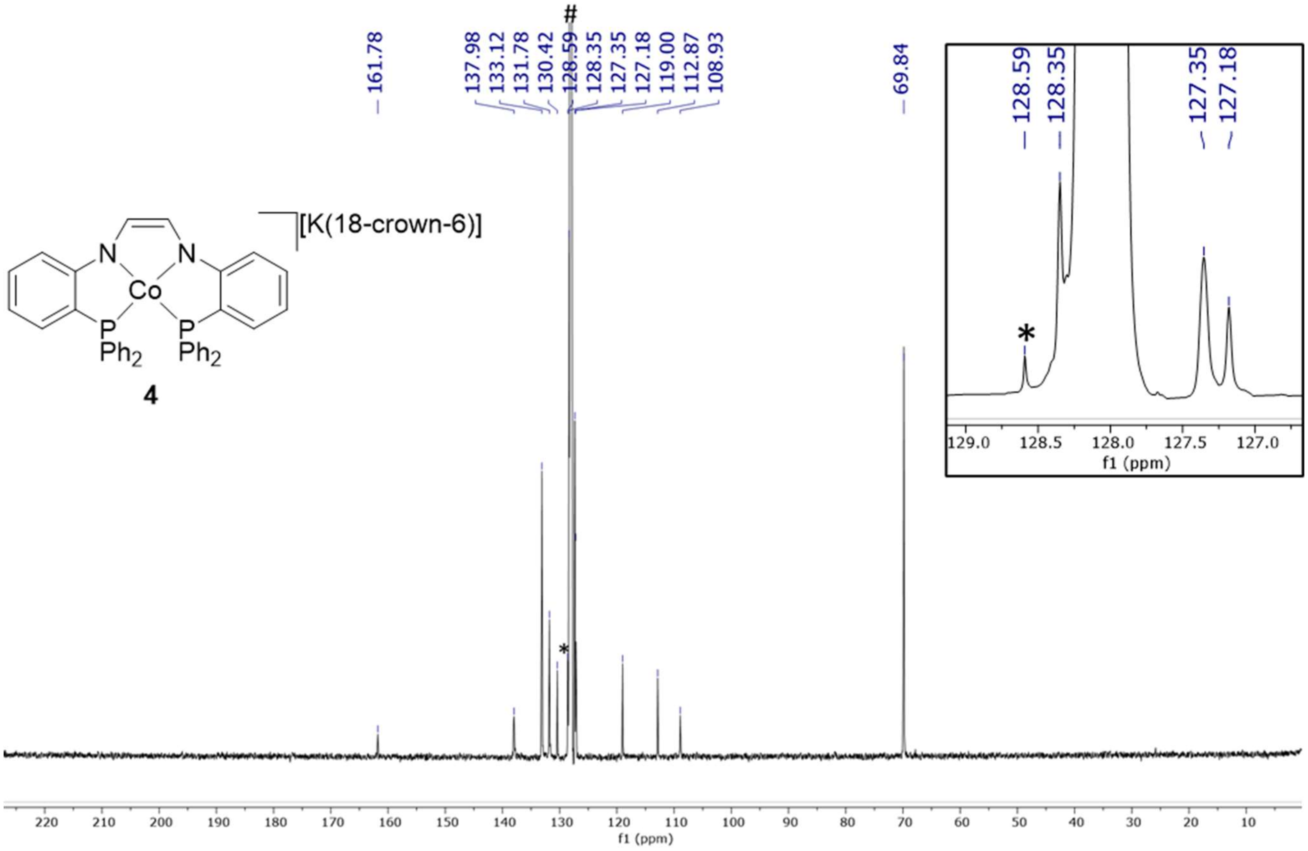

Figure S11. $^{31}P\{^1H\}$ NMR of **4** (162 MHz in C_6D_6).

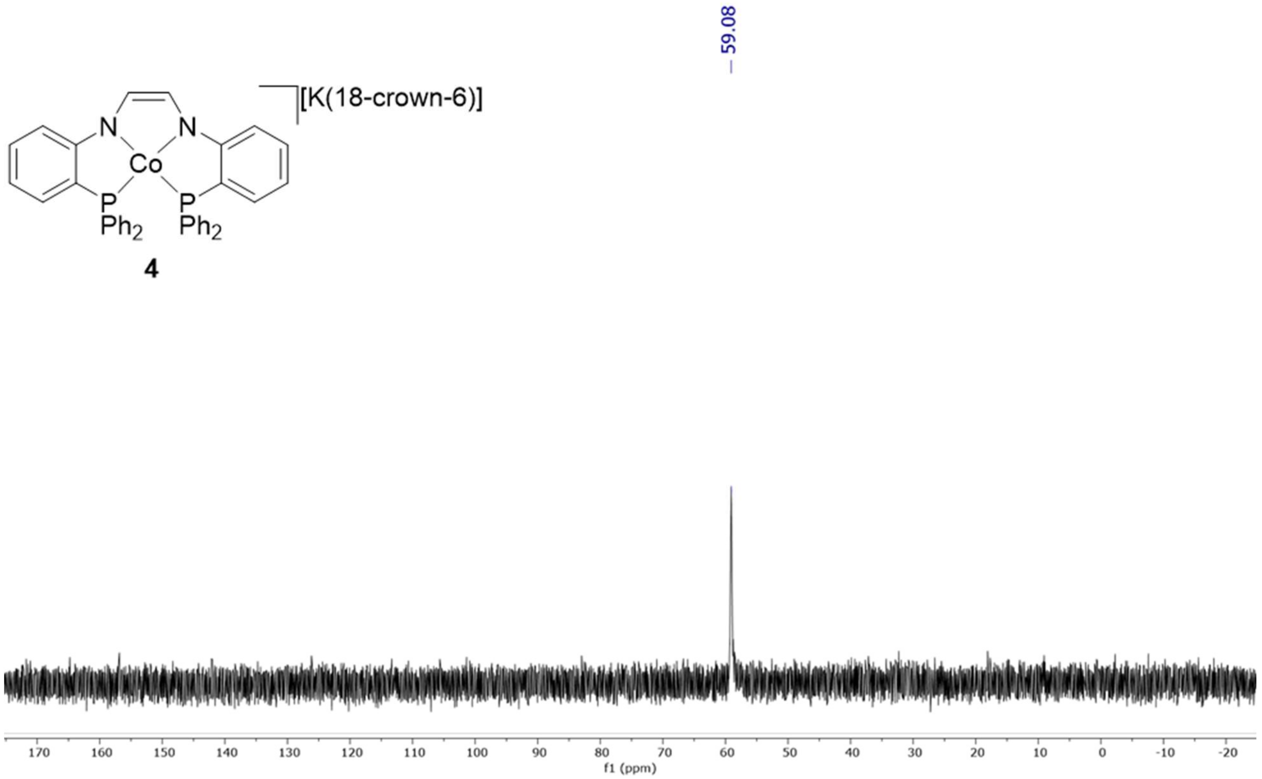

Figure S12. ^{13}C-^1H HSQC spectrum (700 MHz, C_6D_6) of **4**. The peak assigned to residual C_6H_6 is labeled.

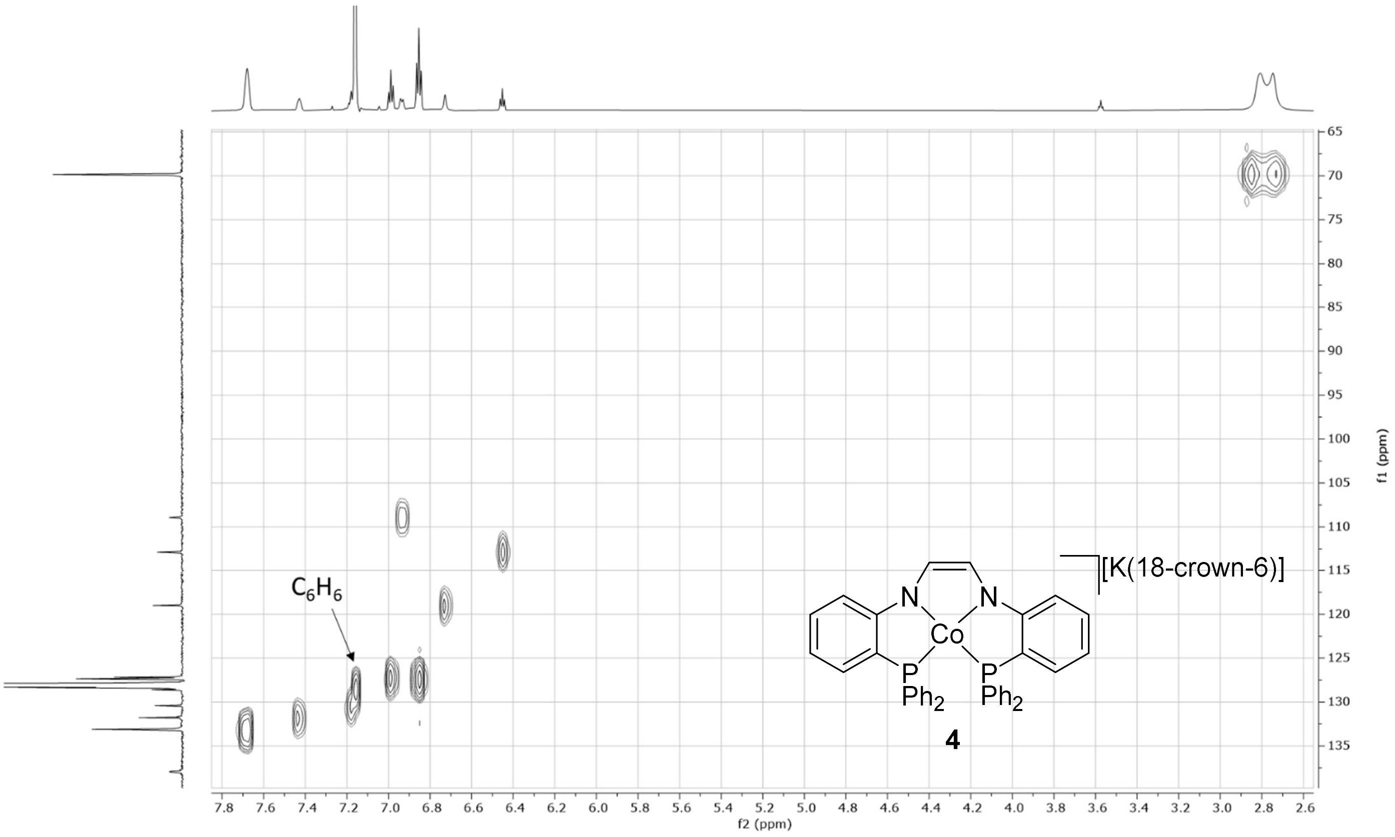

Figure S13. ^{13}C-^{1}H HMBC spectrum of **4** (700 MHz, C_6D_6).

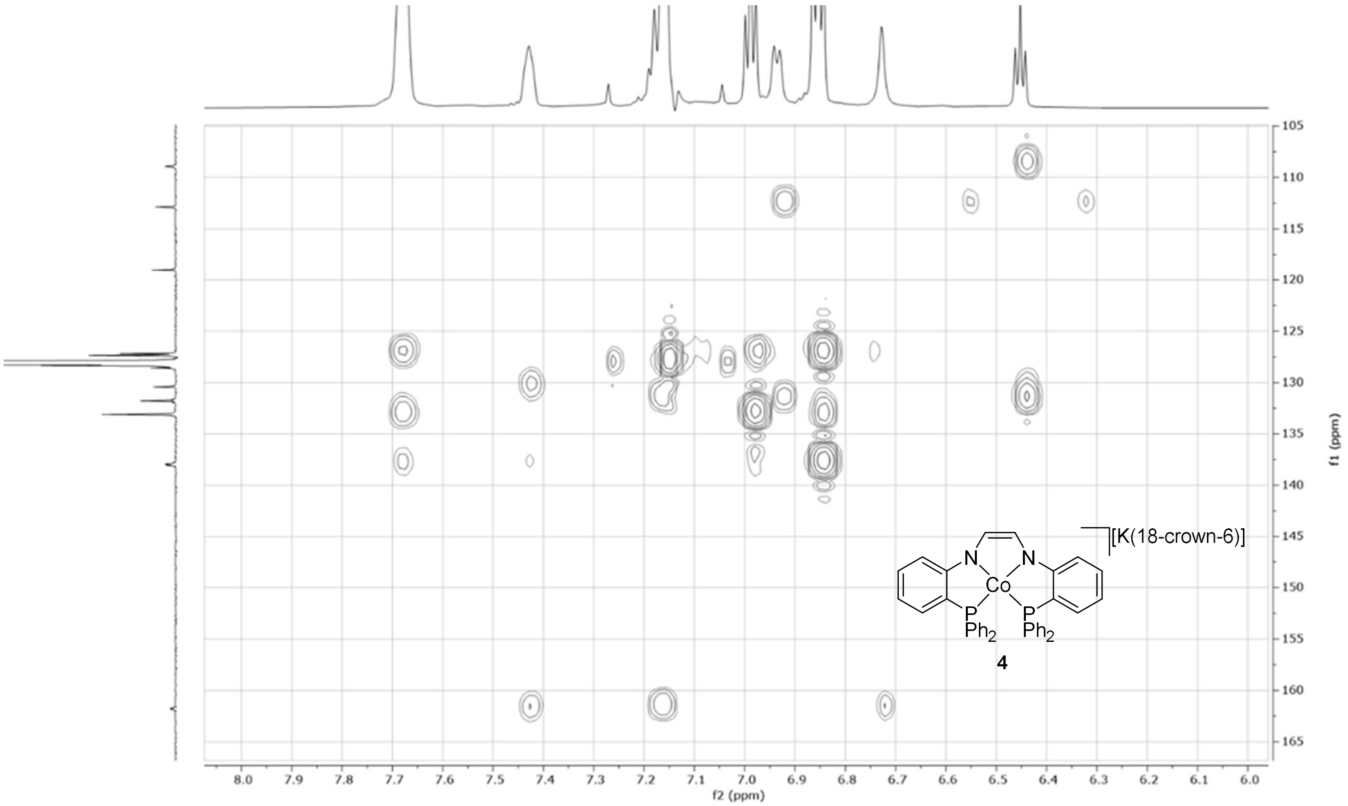

Figure S14. ^{1}H NMR spectrum of **5** (400 MHz, CD_2Cl_2). Stars denote residual solvent impurities (diethyl ether and hexanes). Residual $CHDCl_2$ is denoted with a #.

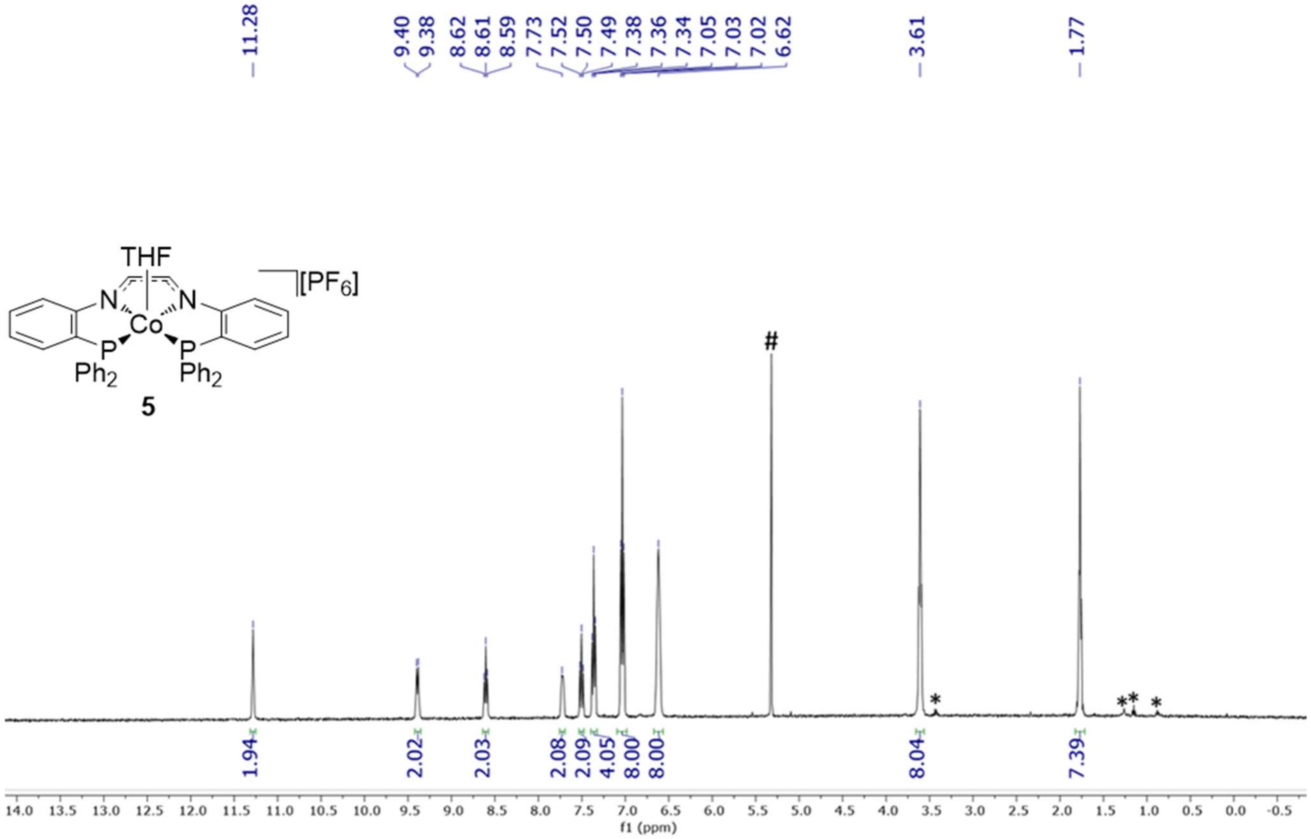

Figure S15. $^{13}C\{^1H\}$ NMR spectrum of **5** (176 MHz, CD_2Cl_2). Residual CD_2Cl_2 is denoted with a #.

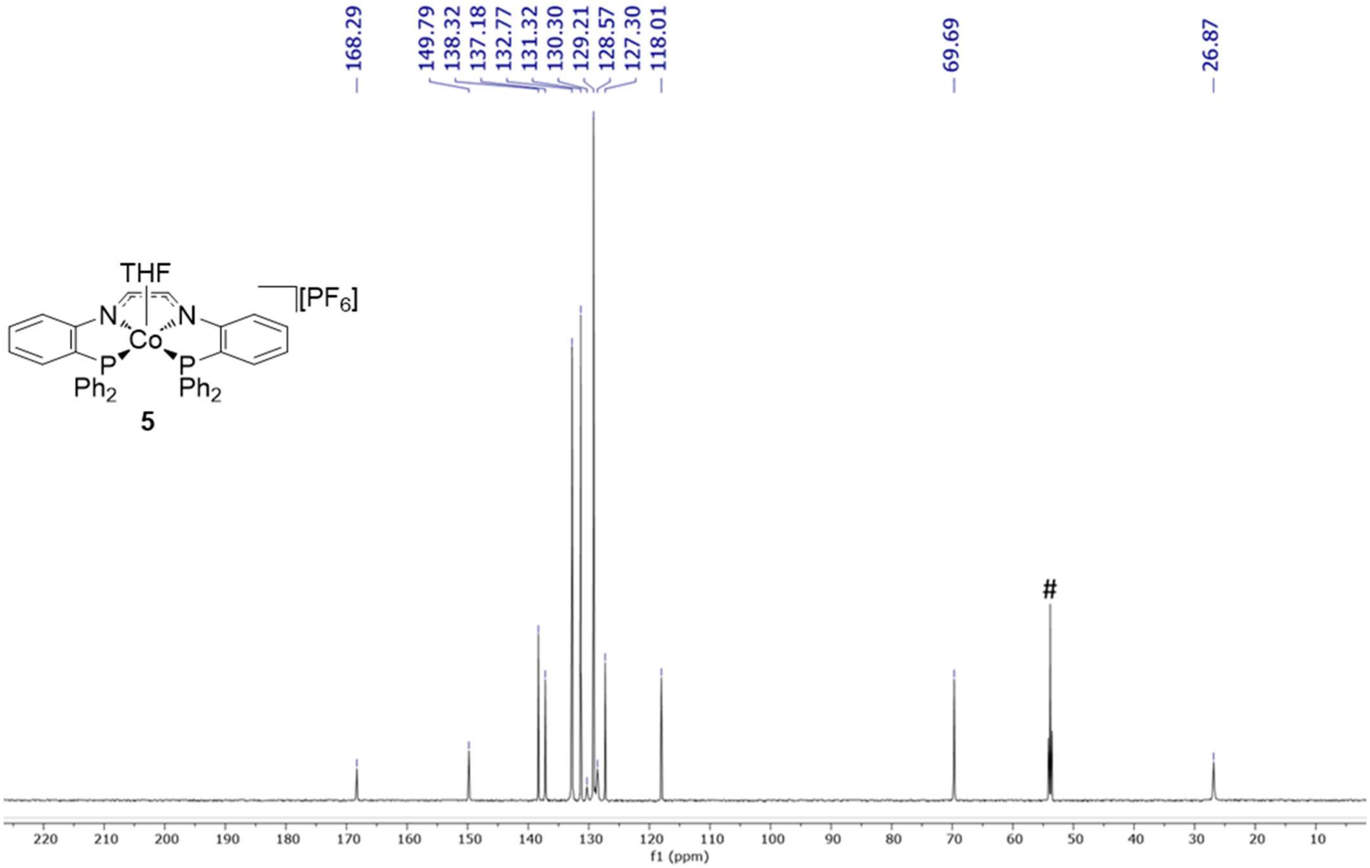

Figure S16. $^{31}P\{^1H\}$ NMR spectrum of **5** (162 MHz, CD_2Cl_2).

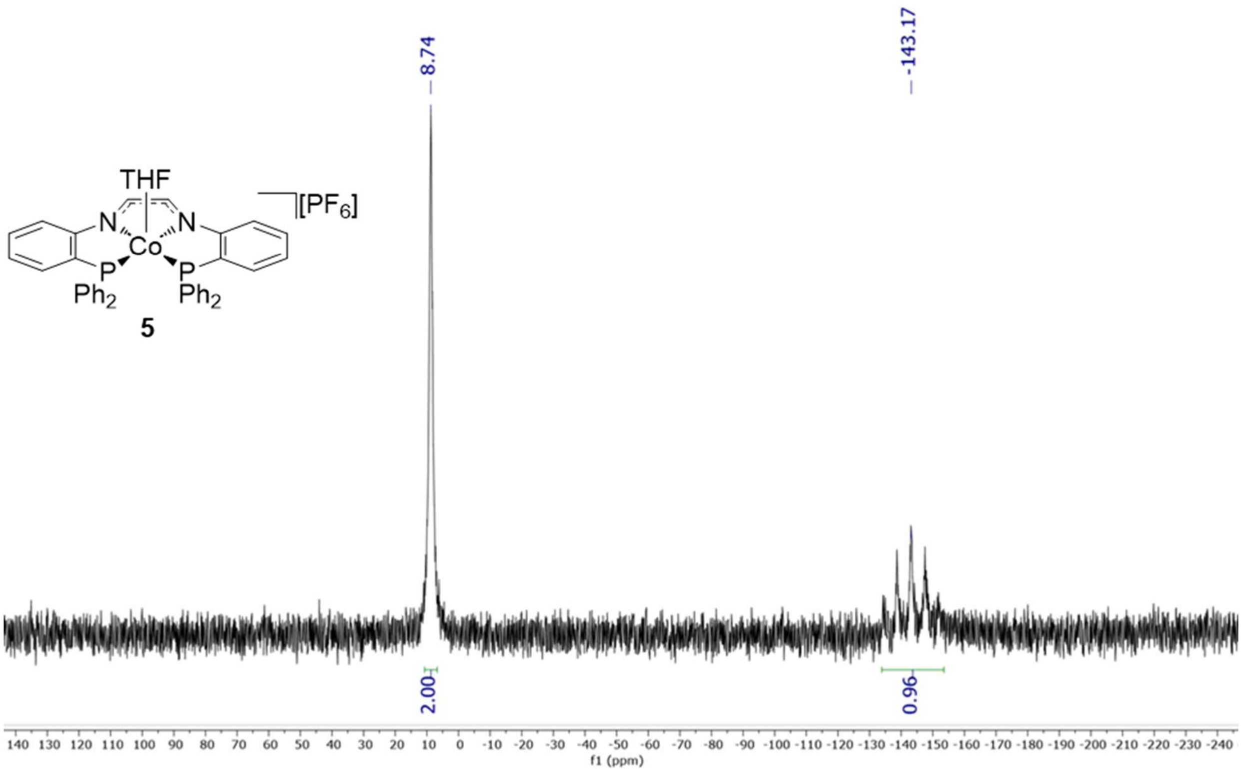

Figure S17. ^{1}H NMR spectrum of **6** (400 MHz, THF-d_8). Stars denote residual pentane and grease. Residual THF is labeled with a #.

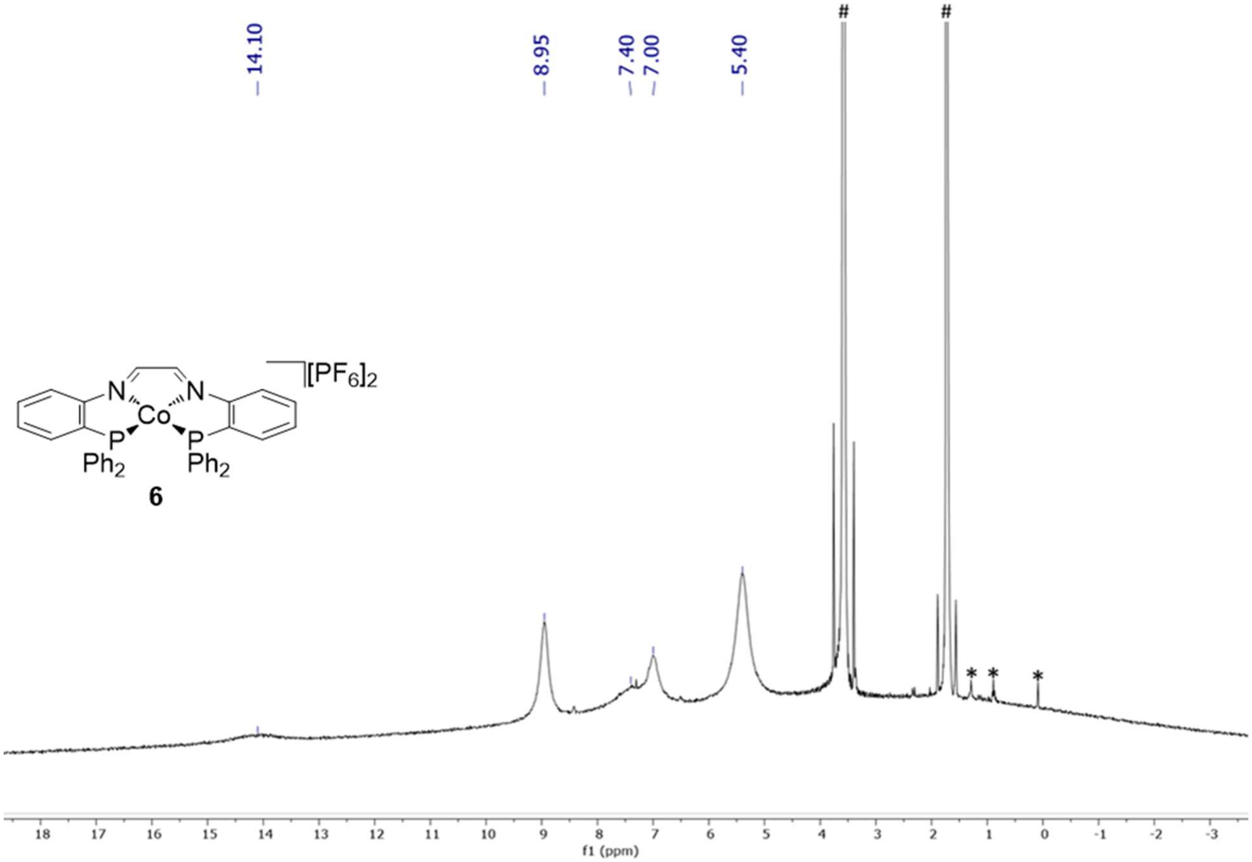

Figure S18. (A) Variable temperature ^1H NMR spectra (600 MHz, CD_2Cl_2) from 290-190 K of **5** (B) ^1H NMR spectrum of **5** at 190 K showing two sets of THF peaks and 11 aromatic peaks, consistent with the reduced symmetry of **5** due to slow exchange of two THF molecules. Residual $CHDCl_2$ is denoted with a # and residual hexanes is labeled with a *.

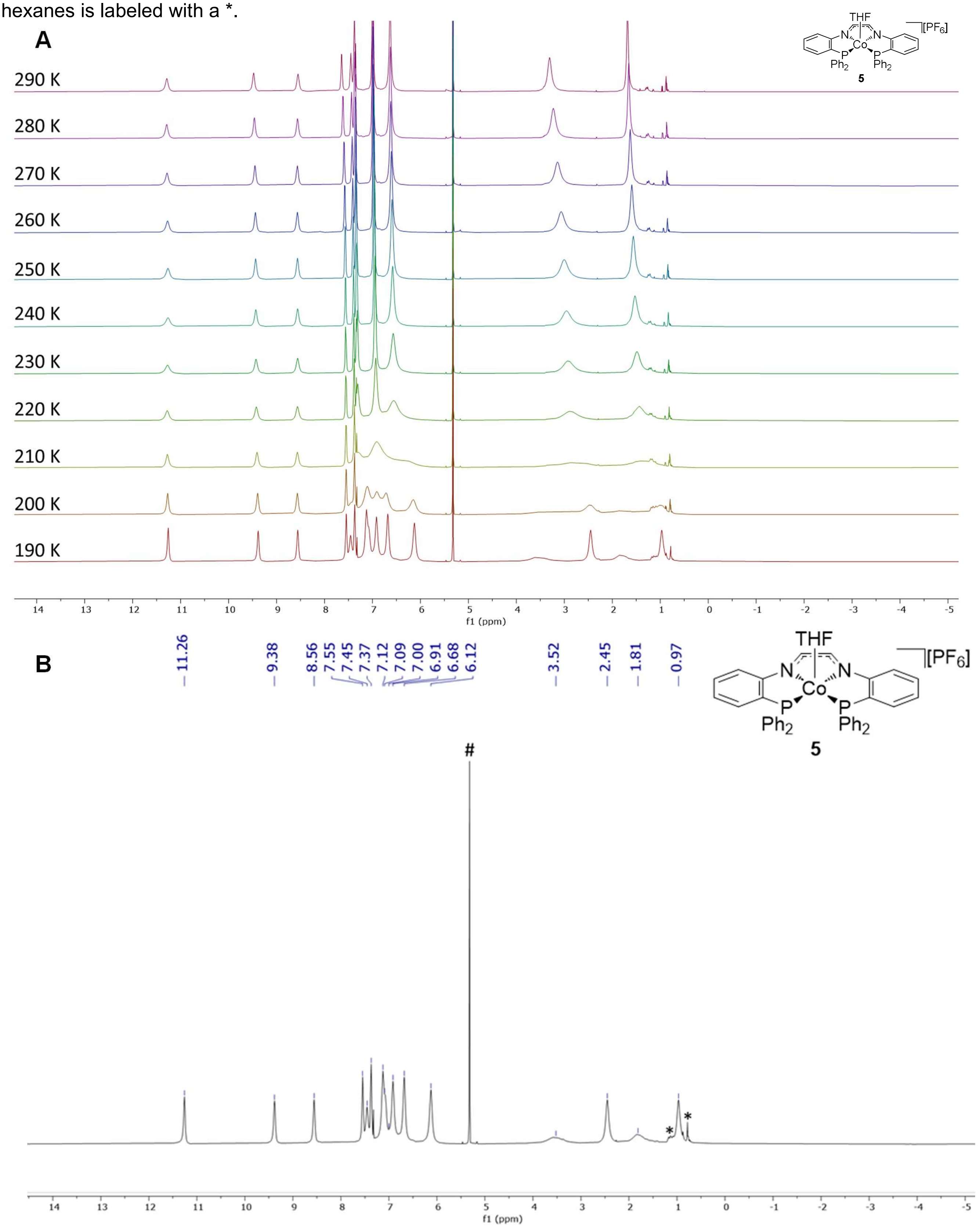

Figure S19. ^{1}H NMR spectrum (400 MHz, C_6D_6) after 4 h of H_2(g) addition to **2**. Residual THF, hexanes, and grease are labeled with a * and residual C_6D_5H is labeled with a #.

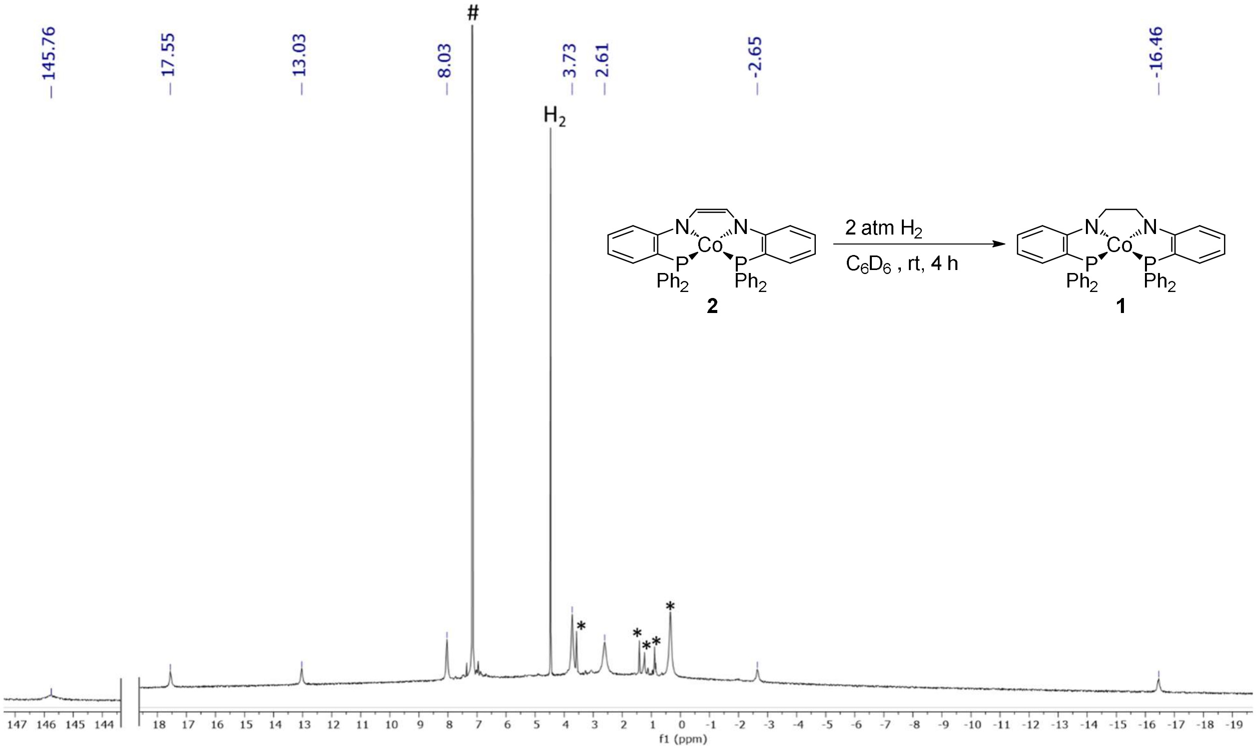

Figure S20. ^{1}H NMR spectrum of **7** (400 MHz, CD_2Cl_2). Stars denote residual solvent impurities (CH_2Cl_2, THF, and acetone). Residual $CHDCl_2$ is denoted with a #.

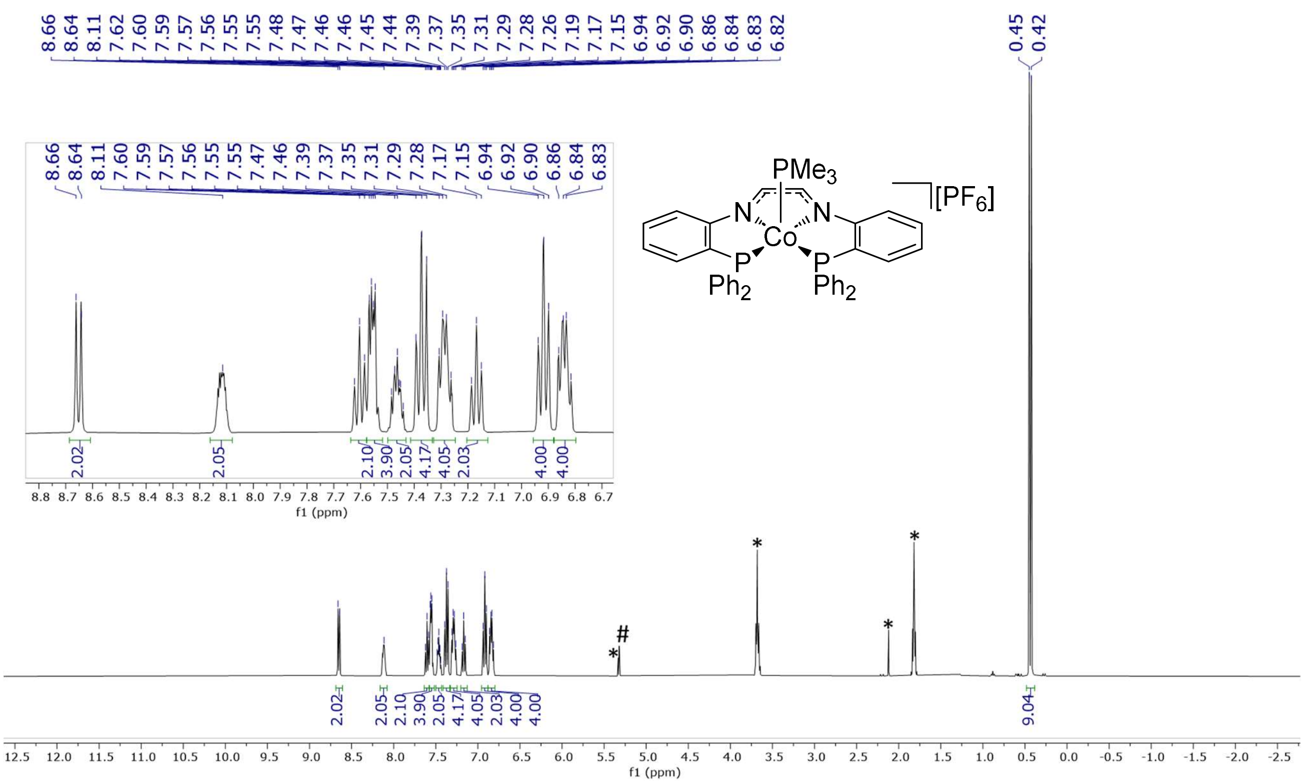

Figure S21. $^{13}C\{^{1}H\}$ NMR spectrum of **7** (100 MHz, CD_2Cl_2). Residual solvent impurities (THF and benzene) are labeled with a star. Residual CD_2Cl_2 is denoted with a #.

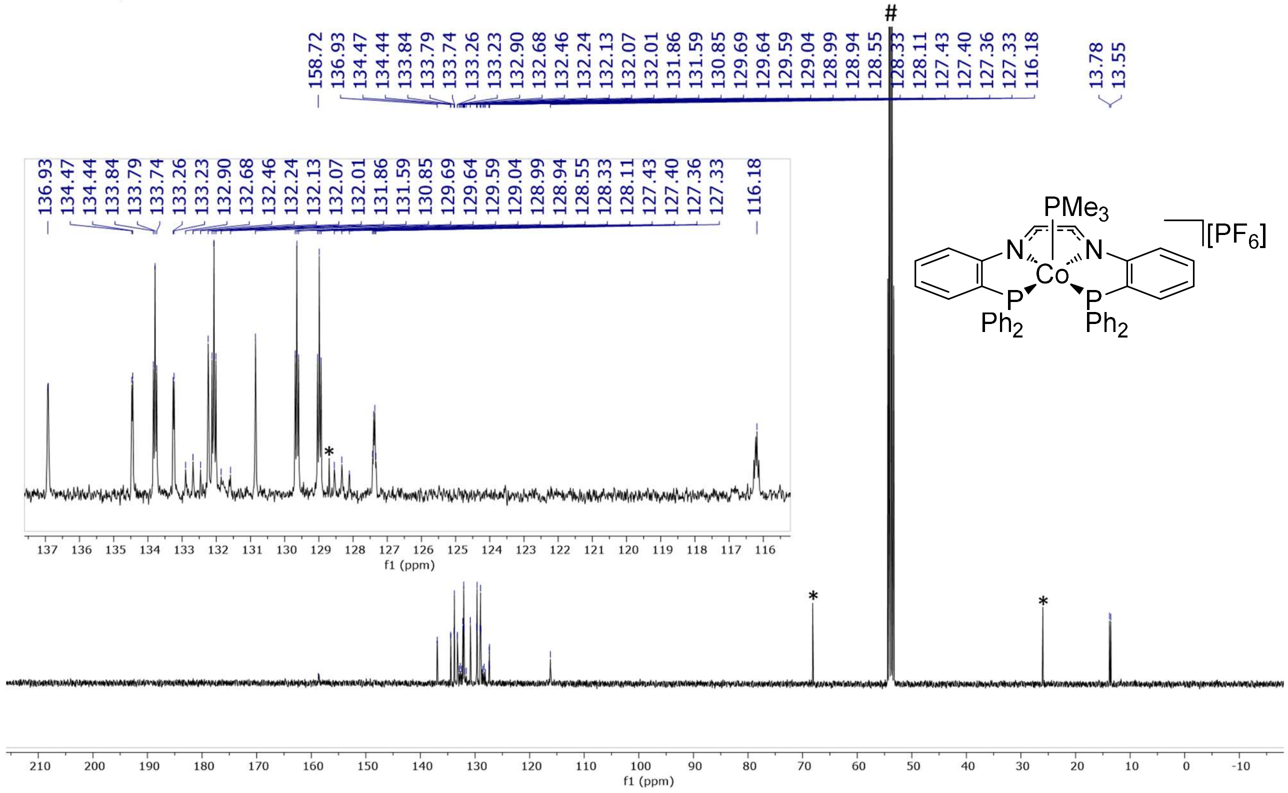

Figure S22. $^{31}P\{^{1}H\}$ NMR spectrum of **7** (162 MHz, CD_2Cl_2).

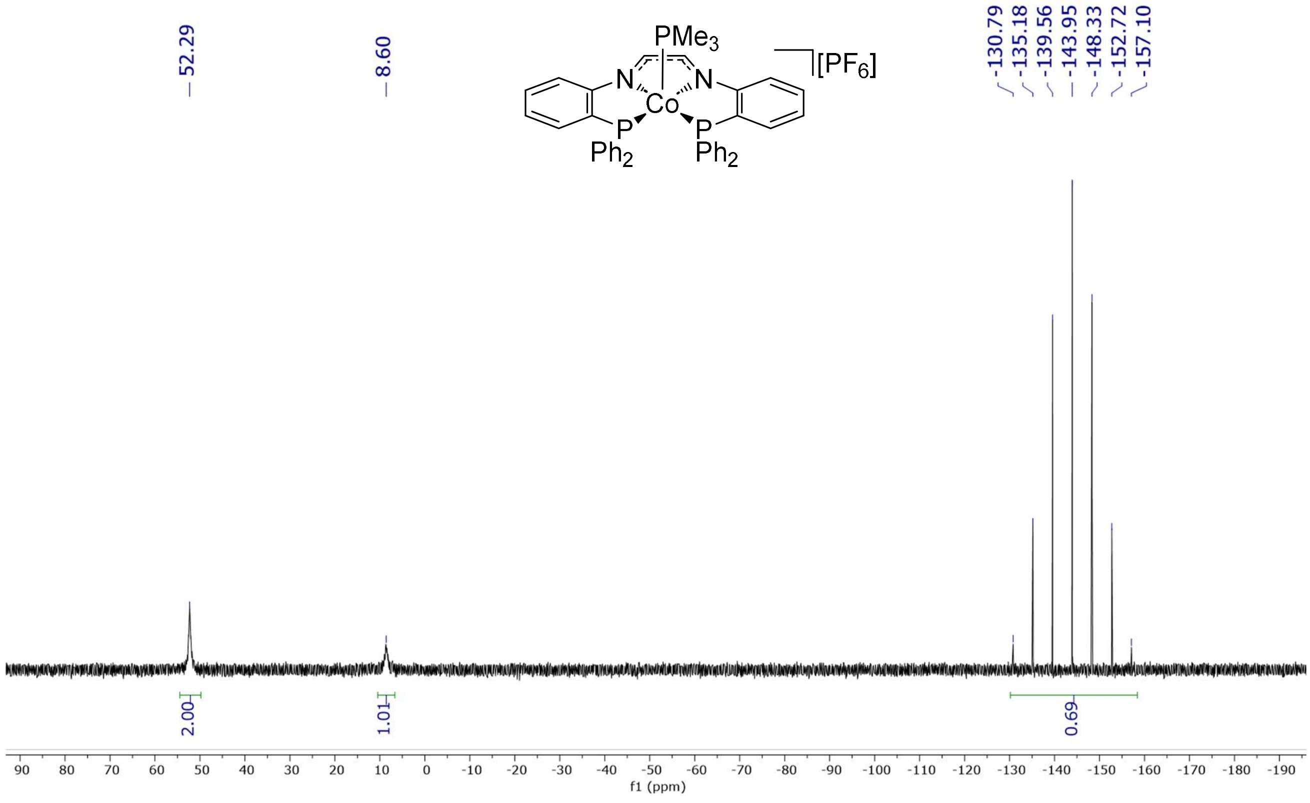

Figure S23. $^{31}P\{^{1}H\}$ NMR (162 MHz) spectra of the attempted hydrogen atom abstraction from ($PNCH_2CH_2NP$)Ni (**1-Ni**). The top and bottom spectra were collected in C_6D_6, while the middle spectrum was collected in THF.

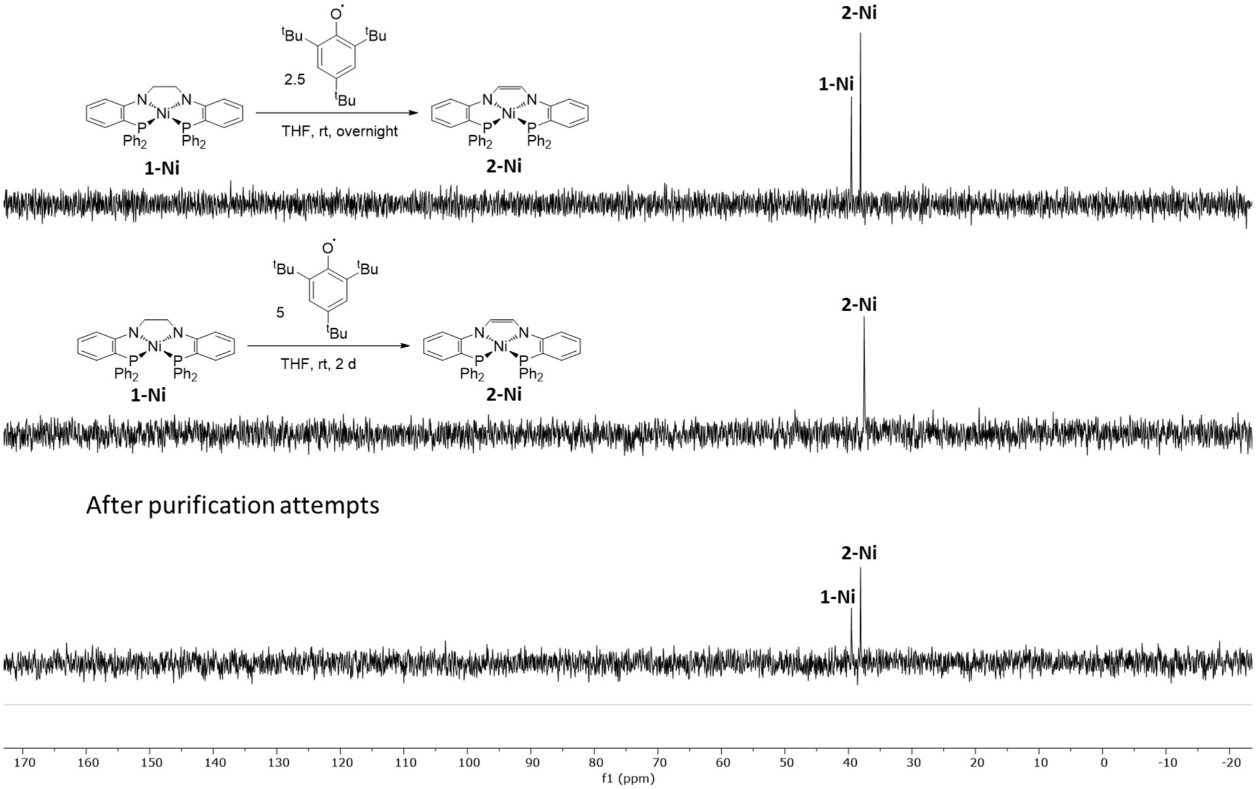

Figure 24. ^{1}H NMR (400 MHz) in C_6D_6 (left) and $^{31}P\{^{1}H\}$ NMR (162 MHz) in C_6D_6 (right) of the reaction between **2** and $KBEt_3H$. Residual CD_5H is labeled with a # and residual THF is labeled with a *.

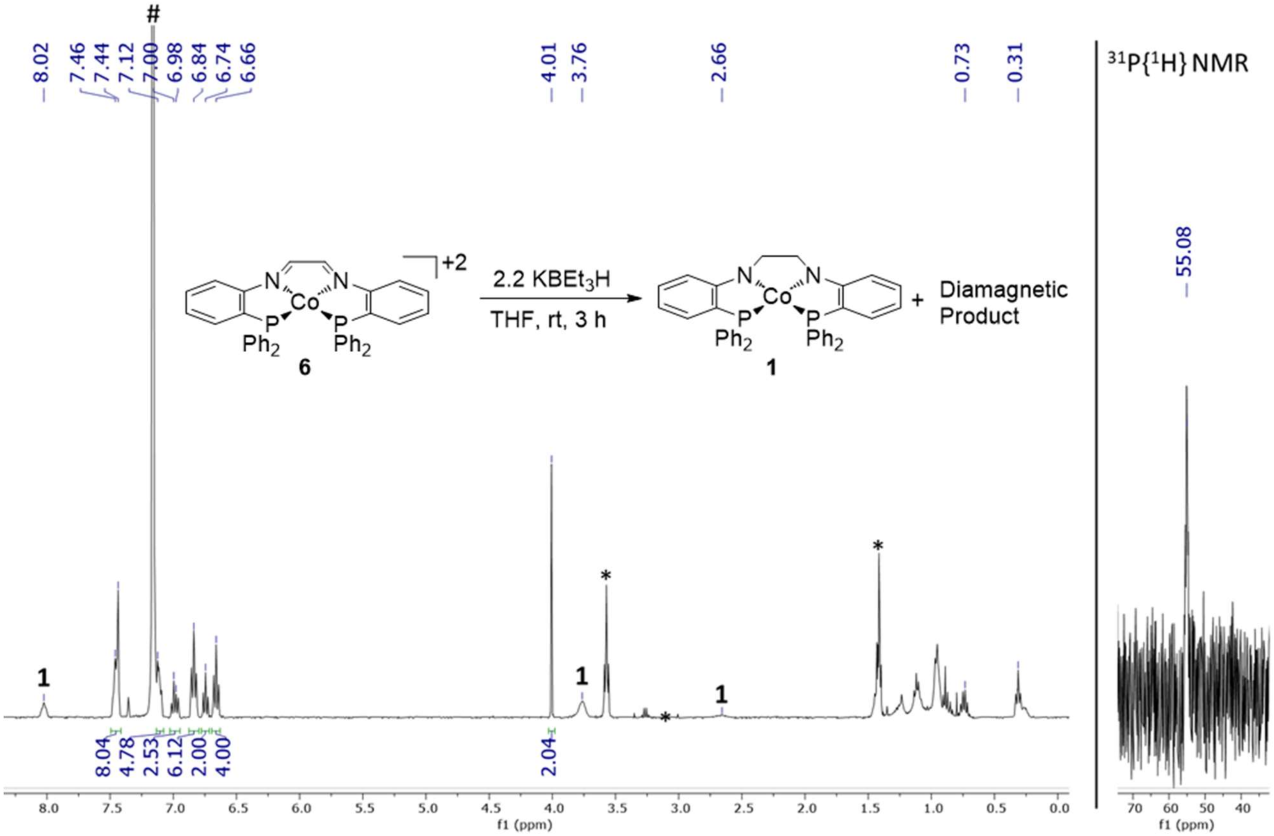

Figure S25. ^{1}H NMR (400 MHz) spectra in C_6D_6 (left) and $^{31}P\{^{1}H\}$ NMR (162 MHz) spectra in C_6D_6 (right) for the reaction between **2** and BuBr. The top spectra are of the crude reaction mixture, the middle spectra are of the hexanes extract, and the bottom spectra are of the PhF extract. Residual C_6D_5H is denoted with a # and residual THF is denoted with a *.

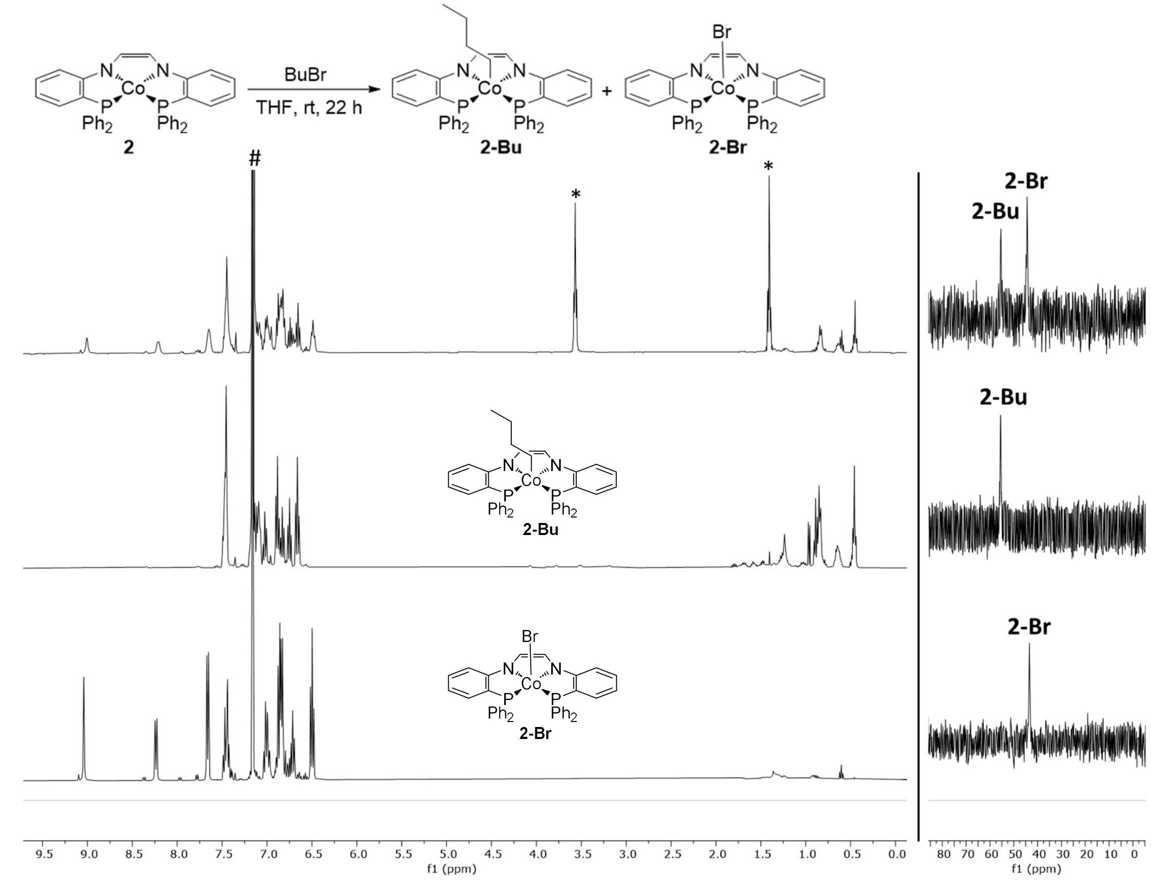

Figure S26. Cyclic voltammogram of $(PNCH_2CH_2NP)Ni$ (**1-Ni**).

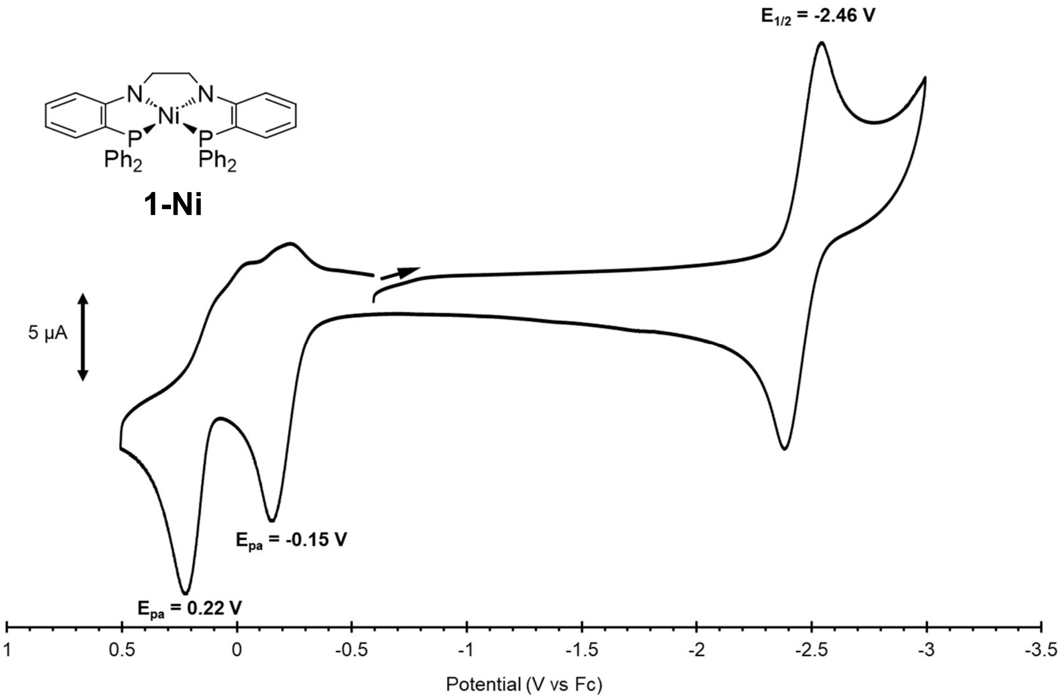

Crystal Structures:

Figure S27. Fully labeled ellipsoid representation (50%) of **1**.

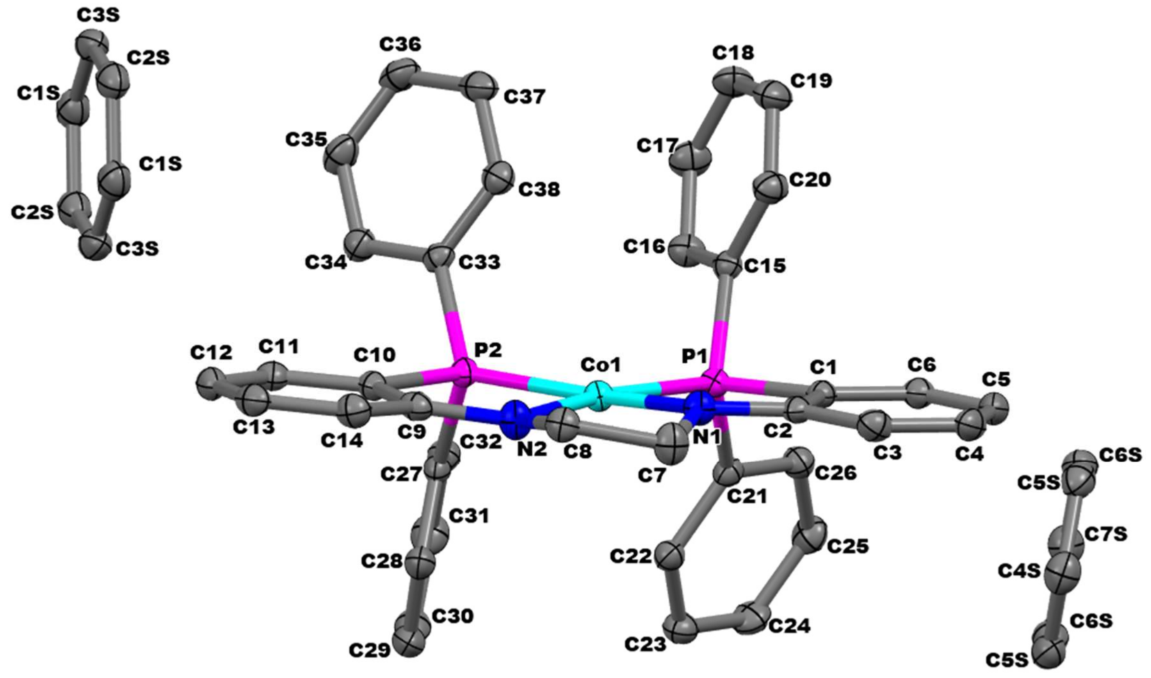

Experimental Summary

The single crystal X-ray diffraction studies were carried out on a Bruker Kappa Photon III CPAD diffractometer equipped with Mo K_α radiation (λ = 0.71073 Å). A 0.103 x 0.054 x 0.047 mm piece of a brown block was mounted on a MiTeGen MicroMount with Paratone 24EX oil. Data were collected in a nitrogen gas stream at 100(2) K using ϕ and ϖ scans. Crystal-to-detector distance was 60 mm using variable exposure time (2-10 s) depending on θ with a scan width of 1.0°. Data collection was 99.9% complete to 25.00° in θ (0.83 Å). A total of 80489 reflections were collected covering the indices, $-46 \le h \le 44$, $-12 \le k \le 12$, $-28 \le l \le 28$. 7051 reflections were found to be symmetry independent, with a R_{int} of 0.0521. Indexing and unit cell refinement indicated a *C*-centered, monoclinic lattice. The space group was found to be *C*2/c. The data were integrated using the Bruker SAINT software program and scaled using the SADABS software program. Solution by dual-space method (SHELXT) produced a complete phasing model for refinement.

All nonhydrogen atoms were refined anisotropically by full-matrix least-squares (SHELXL-2014). All hydrogen atoms were placed using a riding model. Their positions were constrained relative to their parent atom using the appropriate HFIX command in SHELXL-2014. Crystallographic data are summarized in Table S1.

Figure S28. Fully labeled ellipsoid representation (50%) of **2**.

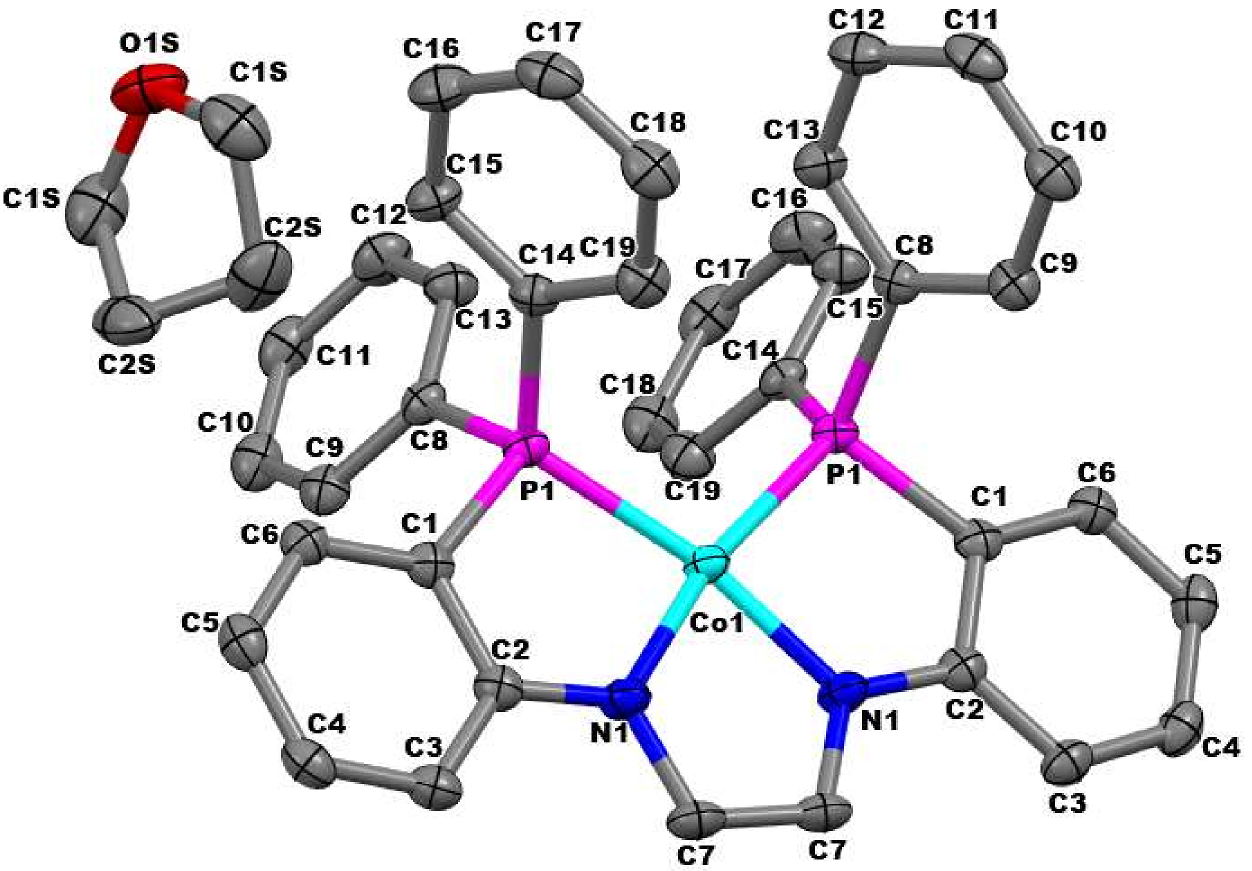

Experimental Summary

The single crystal X-ray diffraction studies were carried out on a Bruker Kappa Photon III CPAD diffractometer equipped with Mo K_{α} radiation (λ = 0.71073 Å). A 0.152 x 0.084 x 0.035 mm piece of a reddish orange block was mounted on a MiTeGen MicroMount with Paratone 24EX oil. Data were collected in a nitrogen gas stream at 100(2) K using ϕ and ϖ scans. Crystal-to-detector distance was 50 mm using variable exposure time (2-5 s) depending on θ with a scan width of 1.0°. Data collection was 99.6% complete to 25.00° in θ (0.83 Å). A total of 21081 reflections were collected covering the indices, $-20 \leq h \leq 20$, $-12 \leq k \leq 12$, $-16 \leq l \leq 17$. 3977 reflections were found to be symmetry independent, with a R_{int} of 0.0294. Indexing and unit cell refinement indicated a *C*-centered, monoclinic lattice. The space group was found to be *C*2. The data were integrated using the Bruker SAINT software program and scaled using the SADABS software program. Solution by dual-space method (SHELXT) produced a complete phasing model for refinement.

All nonhydrogen atoms were refined anisotropically by full-matrix least-squares (SHELXL-2014). All hydrogen atoms were placed using a riding model. Their positions were constrained relative to their parent atom using the appropriate HFIX command in SHELXL-2014. Crystallographic data are summarized in Table S1.

Figure S29. Fully labeled ellipsoid representation (50%) of **3**.

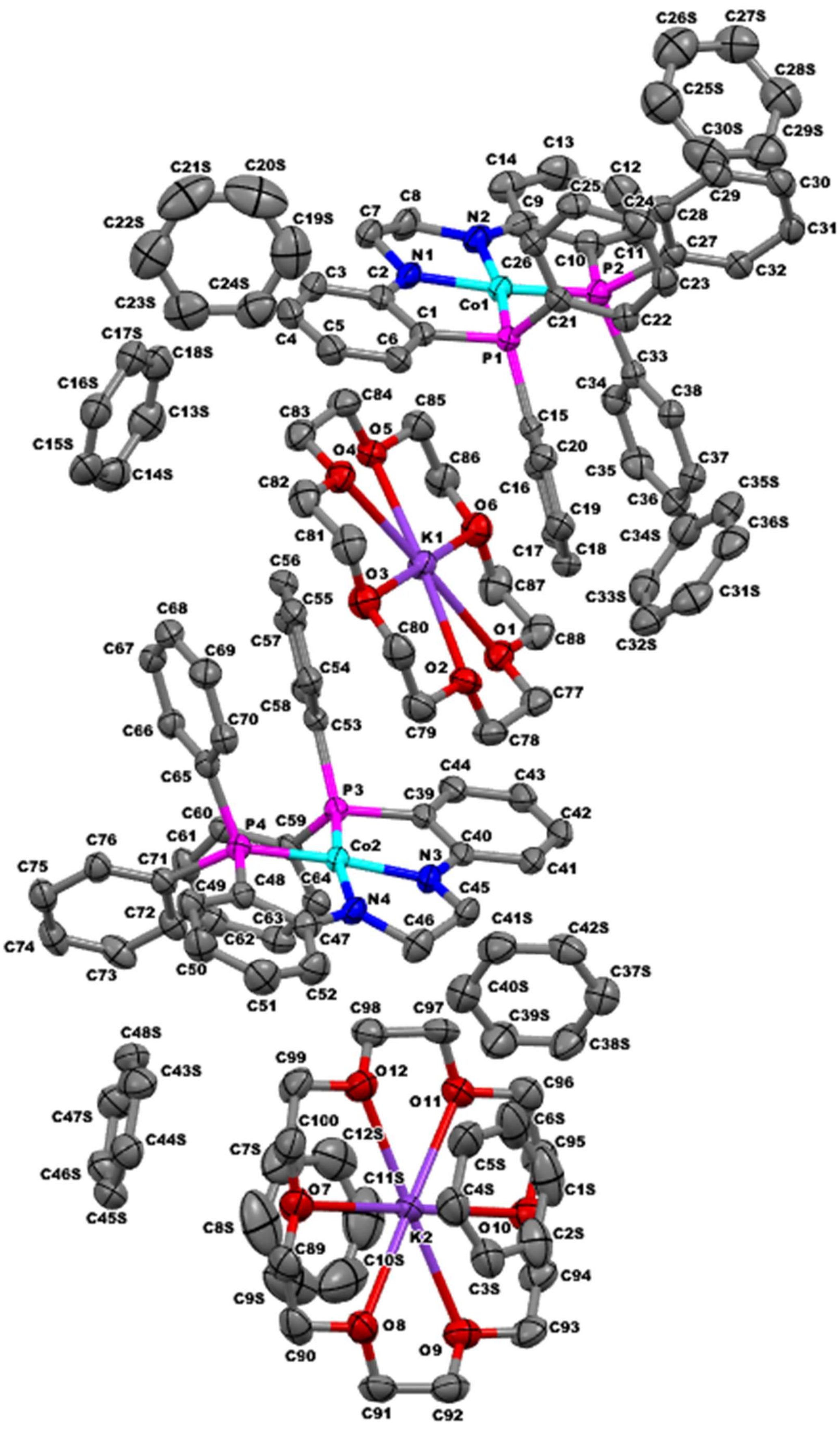

Experimental Summary

The single crystal X-ray diffraction studies were carried out on a Bruker Kappa Photon III CPAD diffractometer equipped with Mo K_α radiation (λ = 0.71073 Å). A 0.184 x 0.172 x 0.124 mm piece of a red block was mounted on a MiTeGen MicroMount with Paratone 24EX oil. Data were collected in a nitrogen gas stream at 100(2) K using ϕ and ϖ scans. Crystal-to-detector distance was 80 mm using variable exposure time (1-20 s) depending on θ with a scan width of 0.75°. Data collection was 99.9% complete to 25.00° in θ (0.83 Å). A total of 292204 reflections were collected covering the indices, $-14 \leq h \leq 10$, $-30 \leq k \leq 30$, $-58 \leq l \leq 58$. 26444 reflections were found to be symmetry independent, with a R_{int} of 0.0533. Indexing and unit cell refinement indicated a primitive, orthorhombic lattice. The space group was found to be $P2_12_12_1$. The data were integrated using the Bruker SAINT software program and scaled using the SADABS software program. Solution by dual-space method (SHELXT) produced a complete phasing model for refinement.

All nonhydrogen atoms were refined anisotropically by full-matrix least-squares (SHELXL-2014). All hydrogen atoms were placed using a riding model. Their positions were constrained relative to their parent atom using the appropriate HFIX command in SHELXL-2014. Crystallographic data are summarized in Table S2.

Figure S30. Fully labeled ellipsoid representation (50%) of **4**.

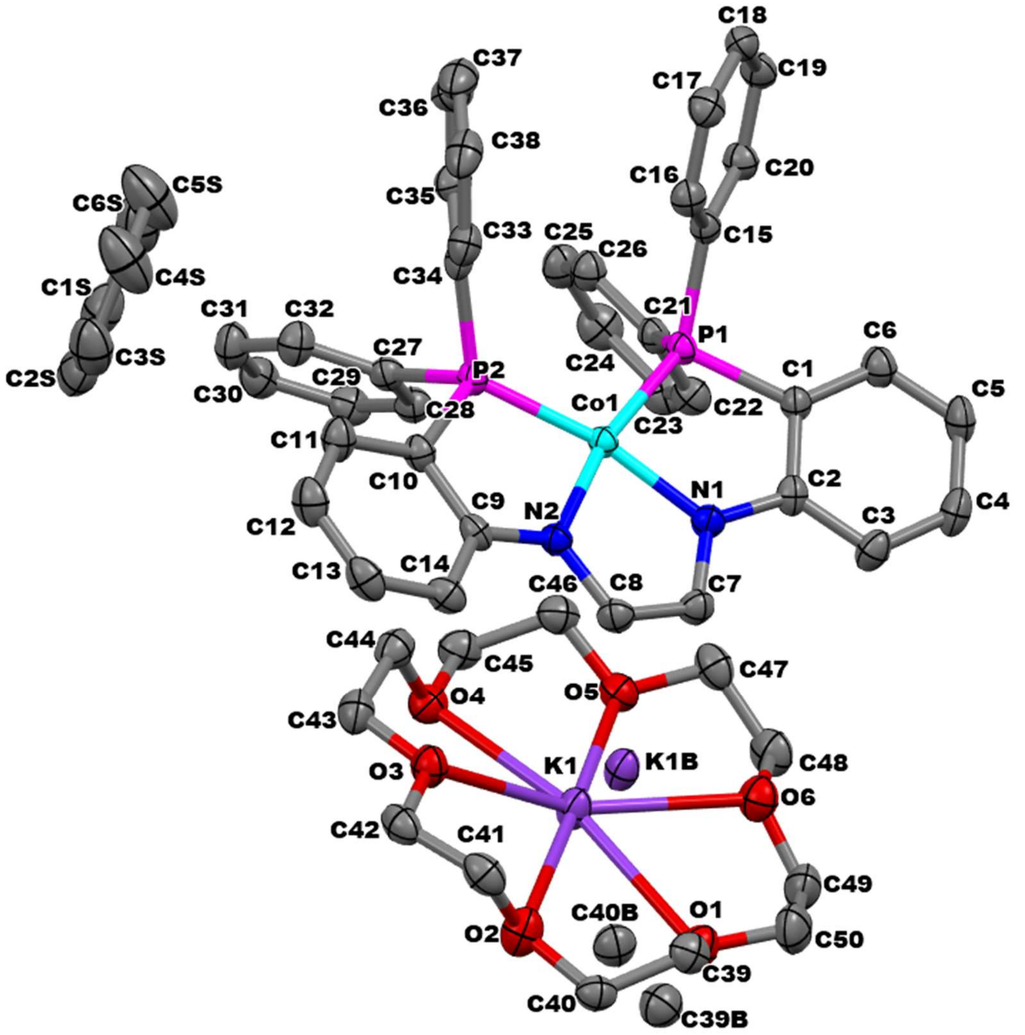

Experimental Summary

The single crystal X-ray diffraction studies were carried out on a Bruker Kappa Photon III CPAD diffractometer equipped with Mo K_α radiation (λ = 0.71073 Å). A 0.132 x 0.086 x 0.054 mm piece of a brown block was mounted on a MiTeGen MicroMount with Paratone 24EX oil. Data were collected in a nitrogen gas stream at 100(2) K using ϕ and ϖ scans. Crystal-to-detector distance was 60 mm using variable exposure time (5-20 s) depending on θ with a scan width of 1.0°. Data collection was 99.9% complete to 25.00° in θ (0.83 Å). A total of 120957 reflections were collected covering the indices, $-13 \leq h \leq 13$, $-17 \leq k \leq 17$, $-22 \leq l \leq 22$. 10186 reflections were found to be symmetry independent, with a R_{int} of 0.0707. Indexing and unit cell refinement indicated a primitive, triclinic lattice. The space group was found to be *P*-1. The data were integrated using the Bruker SAINT software program and scaled using the SADABS software program. Solution by dual-space method (SHELXT) produced a complete phasing model for refinement.

All nonhydrogen atoms were refined anisotropically by full-matrix least-squares (SHELXL-2014). All hydrogen atoms were placed using a riding model. Their positions were constrained relative to their parent atom using the appropriate HFIX command in SHELXL-2014. Crystallographic data are summarized in Table S2.

Figure S31. Fully labeled ellipsoid representation (50%) of **5**.

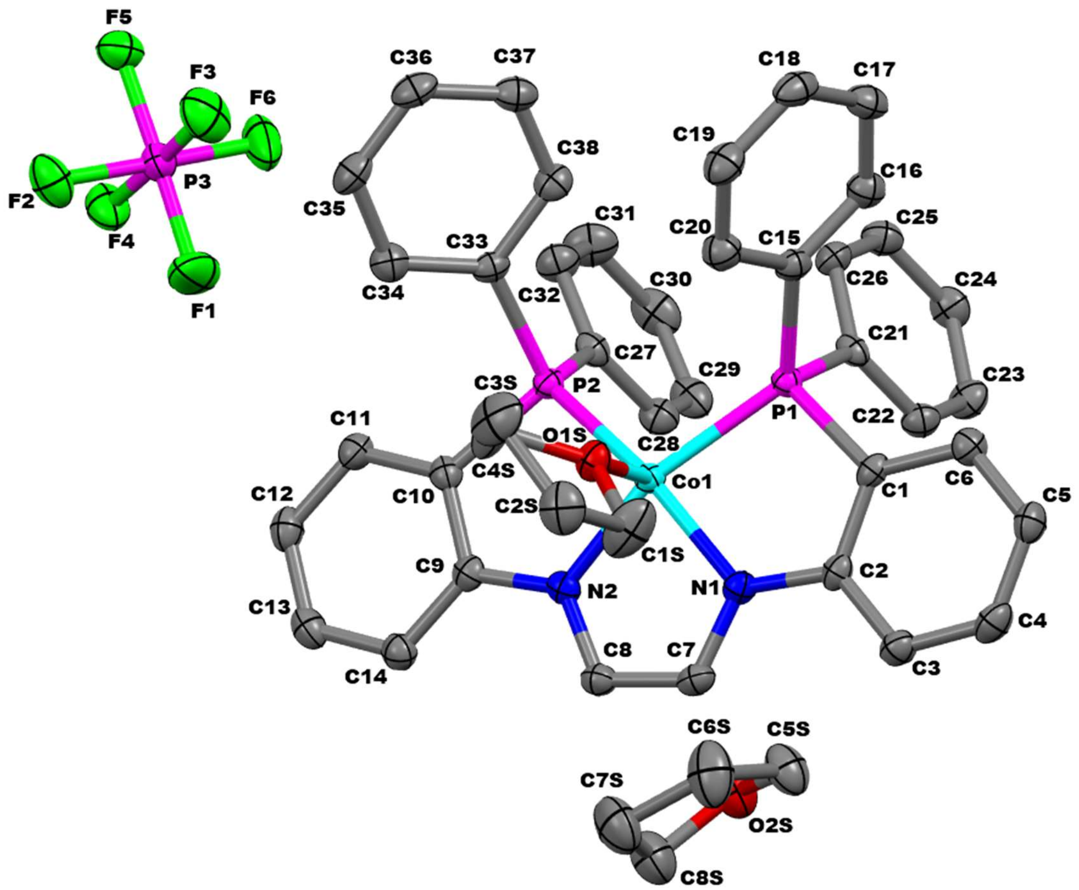

Experimental Summary

The single crystal X-ray diffraction studies were carried out on a Bruker Kappa Photon III CPAD diffractometer equipped with Mo K_α radiation (λ = 0.71073 Å). A 0.110 x 0.098 x 0.040 mm piece of a purple block was mounted on a MiTeGen MicroMount with Paratone 24EX oil. Data were collected in a nitrogen gas stream at 100(2) K using ϕ and ϖ scans. Crystal-to-detector distance was 70 mm using variable exposure time (1-10 s) depending on θ with a scan width of 0.75°. Data collection was 99.9% complete to 25.00° in θ (0.83 Å). A total of 137217 reflections were collected covering the indices, -13 ≤ *h* ≤ 13, -16 ≤ *k* ≤ 16, -17 ≤ *l* ≤ 17. 8507 reflections were found to be symmetry independent, with a R_{int} of 0.0847. Indexing and unit cell refinement indicated a primitive, monoclinic lattice. The space group was found to be $P2_1$. The data were integrated using the Bruker SAINT software program and scaled using the TWINABS software program. Solution by dual-space method (SHELXT) produced a complete phasing model for refinement.

All nonhydrogen atoms were refined anisotropically by full-matrix least-squares (SHELXL-2014). All hydrogen atoms were placed using a riding model. Their positions were constrained relative to their parent atom using the appropriate HFIX command in SHELXL-2014. Crystallographic data are summarized in Table S3.

Figure S32. Fully labeled ellipsoid representation (50%) of **6**.

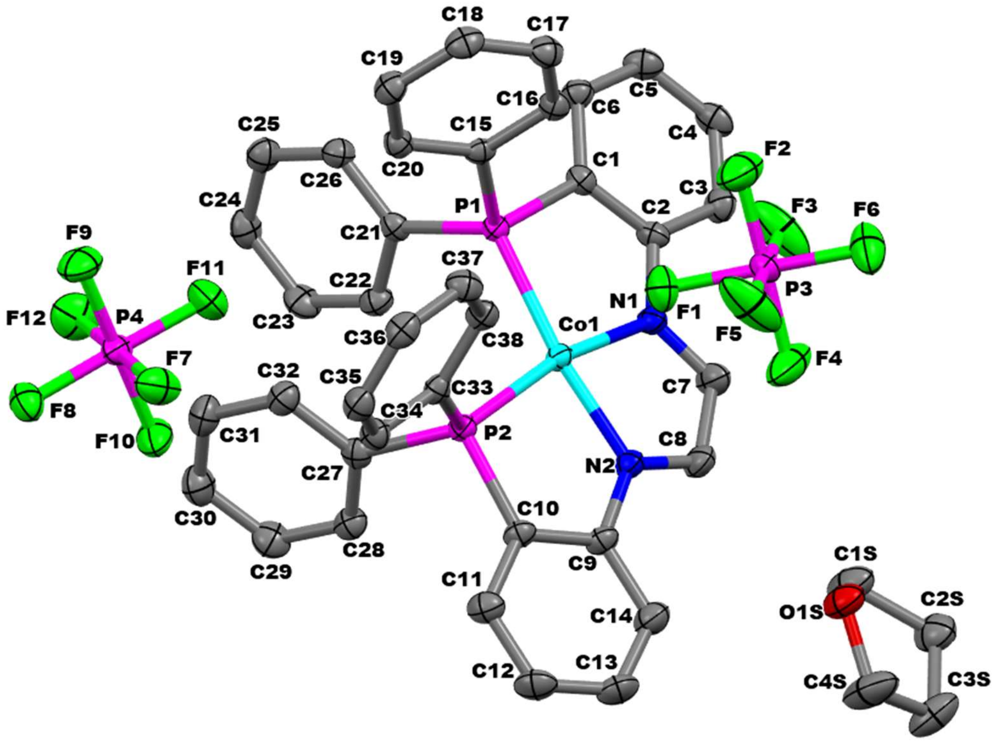

Experimental Summary

The single crystal X-ray diffraction studies were carried out on a Bruker Kappa Photon III CPAD diffractometer equipped with Mo K_α radiation (λ = 0.71073 Å). A 0.146 x 0.137 x 0.057 mm piece of a green block was mounted on a MiTeGen MicroMount with Paratone 24EX oil. Data were collected in a nitrogen gas stream at 100(2) K using ϕ and ϖ scans. Crystal-to-detector distance was 60 mm using variable exposure time (1-5s) depending on θ with a scan width of 1.0°. Data collection was 99.9% complete to 25.00° in θ (0.83 Å). A total of 93637 reflections were collected covering the indices, $-19 \leq h \leq 19$, $-17 \leq k \leq 17$, $-23 \leq l \leq 23$. 8426 reflections were found to be symmetry independent, with a R_{int} of 0.0588. Indexing and unit cell refinement indicated a primitive, monoclinic lattice. The space group was found to be $Pca2_1$. The data were integrated using the Bruker SAINT software program and scaled using the SADABS software program. Solution by dual-space method (SHELXT) produced a complete phasing model for refinement.

All nonhydrogen atoms were refined anisotropically by full-matrix least-squares (SHELXL-2014). All hydrogen atoms were placed using a riding model. Their positions were constrained relative to their parent atom using the appropriate HFIX command in SHELXL-2014. Crystallographic data are summarized in Table S3.

Figure S33. Fully labeled ellipsoid representation (50%) of **7**.

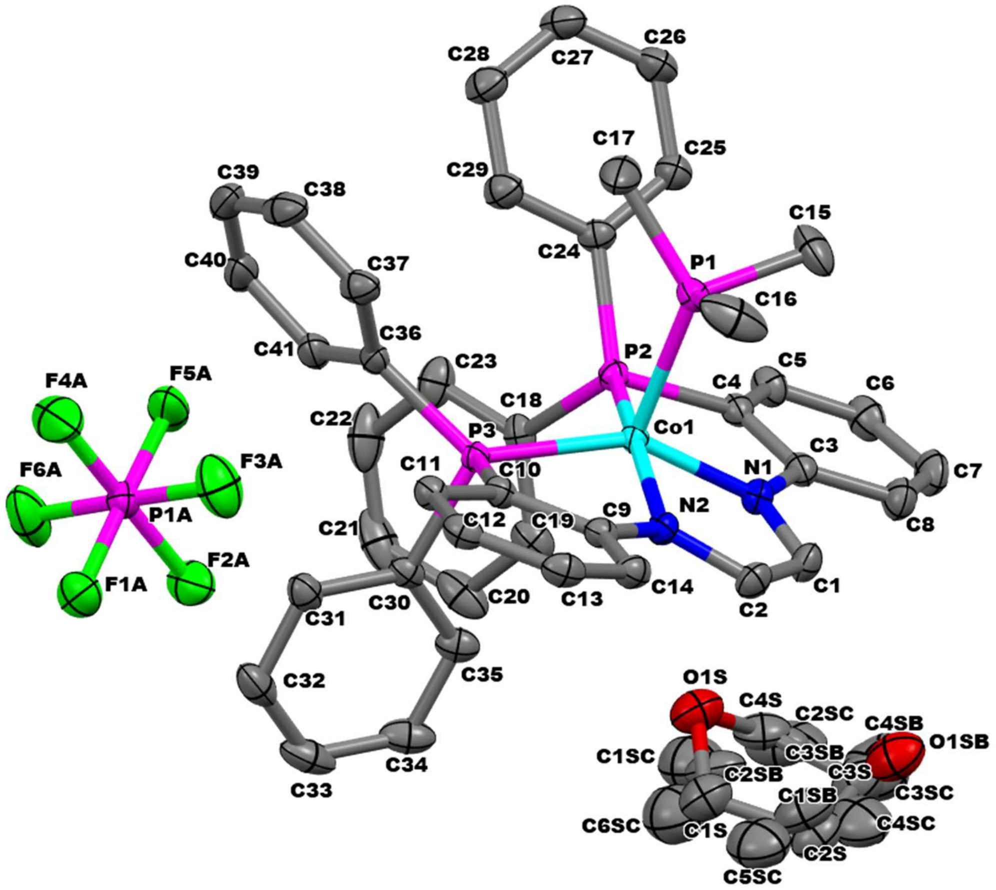

Experimental Summary

The single crystal X-ray diffraction studies were carried out on a Bruker Kappa Photon III CPAD diffractometer equipped with Mo K_α radiation (λ = 0.71073 Å). A 0.132 x 0.086 x 0.084 mm piece of a red block was mounted on a MiTeGen MicroMount with Paratone 24EX oil. Data were collected in a nitrogen gas stream at 100(2) K using ϕ and ϖ scans. Crystal-to-detector distance was 60 mm using variable exposure time (1s-2s) depending on θ with a scan width of 1.0°. Data collection was 99.9% complete to 25.00° in θ (0.83 Å). A total of 96743 reflections were collected covering the indices, $-14 \leq h \leq 14$, $-15 \leq k \leq 15$, $-19 \leq l \leq 19$. 8804 reflections were found to be symmetry independent, with a R_{int} of 0.0404. Indexing and unit cell refinement indicated a primitive, triclinic lattice. The space group was found to be *P*-1. The data were integrated using the Bruker SAINT software program and scaled using the SADABS software program. Solution by dual-space method (SHELXT) produced a complete phasing model for refinement.

All nonhydrogen atoms were refined anisotropically by full-matrix least-squares (SHELXL-2014). All hydrogen atoms were placed using a riding model. Their positions were constrained relative to their parent atom using the appropriate HFIX command in SHELXL-2014. Crystallographic data are summarized in Table S4.

Crystal Structure Refinement Data

Table S1. Crystal data and structure refinement for **1** and **2**.

	**1**●*C_6H_6*	**2**●THF
CCDC number	2354342	2354341
Empirical Formula	$C_{44}H_{38}CoN_2P_2$	$C_{42}H_{38}CoN_2OP_2$
Molecular Formula	$C_{38}H_{32}CoN_2P_2$, C_6H_6	$C_{38}H_{30}CoN_2P_2$, C_4H_8O
Formula Weight (g/mol)	715.63	707.61
Temperature (K)	100.0	100.0
Wavelength (Å)	0.71073	0.71073
Crystal System	Monoclinic	Monoclinic
Space Group	*C*2/c	*C*2
a (Å)<br>b (Å)<br>c (Å)<br>α (°)<br>β (°)<br>γ (°)	37.0504(12)<br>9.6501(4)<br>23.1185(8)<br>90<br>123.2110(10)<br>90	15.4283(9)<br>9.7434(5)<br>12.9567(8)<br>90<br>120.359(2)<br>90
Volume (Å^3)	6915.6(4)	1680.63(17)
Z	8	2
Density (calculated) (g/cm^3)	1.375	1.398
Absorption Coefficient (mm^{-1})	0.625	0.644
F(000)	2984	738
Crystal Size (mm^3)	0.103 x 0.054 x 0.047	0.152 x 0.084 x 0.035
Crystal color, habit	brown block	reddish orange block
Final R indices [I>2σ(I)]	R_1 = 0.0289, wR_2 = 0.0709	R_1 = 0.0273, wR_2 = 0.0622
R indices (all data)	R_1 = 0.0371, wR_2 = 0.0760	R_1 = 0.0294, wR_2 = 0.0632

Table S2. Crystal data and structure refinement for **3** and **4**.

	**3**●*4C$_6$H$_6$*	**4**●C$_6$H$_6$
CCDC Number	2354345	2354344
Empirical Formula	$C_{74}H_{80}CoKN_2O_6P_2$	$C_{56}H_{60}CoKN_2O_6P_2$
Molecular Formula	$C_{38}H_{32}CoN_2P_2$, $C_{12}H_{24}KO_6$, 4(C_6H_6)	$C_{50}H_{54}CoKN_2O_6P_2$, C_6H_6
Formula Weight (g/mol)	1253.37	1017.03
Temperature (K)	100.0	100.0
Wavelength (Å)	0.71073	0.71073
Crystal System	Orthorhombic	Triclinic
Space Group	$P2_12_12_1$	*P*-1
a (Å)	11.2827(5)	11.1913(4)
b (Å)	24.3783(12)	13.9843(6)
c (Å)	46.956(2)	17.7609(8)
α (°)	90	76.6020(10)
β (°)	90	83.3960(10)
γ (°)	90	67.3580(10)
Volume (Å^3)	12915.5(11)	2494.55(18)
Z	8	2
Density (calculated) (g/cm^3)	1.289	1.354
Absorption Coefficient (mm^{-1})	0.435	0.545
F(000)	5296	1068
Crystal Size (mm^3)	0.184 x 0.172 x 0.124	0.132 x 0.086 x 0.054
Crystal color, habit	Red block	Brown block
Final R indices [$I>2\sigma(I)$]	R_1 = 0.0431, wR_2 = 0.1093	R_1 = 0.0393, wR_2 = 0.0897
R indices (all data)	R_1 = 0.0544, wR_2 = 0.1181	R_1 = 0.0626, wR_2 = 0.1011

Table S3. Crystal data and structure refinement for **5** and **6**.

	**5**●THF	**6**●THF
CCDC Number	2354340	2354343
Empirical Formula	$C_{46}H_{46}CoF_6N_2O_2P_3$	$C_{42}H_{38}CoF_{12}N_2OP_4$
Molecular Formula	$C_{42}H_{38}CoN_2OP_2$, F_6P, C_4H_8O	$C_{38}H_{30}CoN_2P_2$, 2(F_6P), C_4H_8O
Formula Weight (g/mol)	924.69	997.55
Temperature (K)	100.0	100.0
Wavelength (Å)	0.71073	0.71073
Crystal System	Monoclinic	Orthorhombic
Space Group	$P2_1$	$Pca2_1$
a (Å)<br>b (Å)<br>c (Å)<br>α (°)<br>β (°)<br>γ (°)	10.8969(4)<br>13.5727(5)<br>14.1536(6)<br>90<br>96.740(2)<br>90	15.4595(4)<br>14.3076(4)<br>18.6069(6)<br>90<br>90<br>90
Volume (Å^3)	2078.86(14)	4115.6
Z	2	4
Density (calculated) (g/cm^3)	1.477	1.610
Absorption Coefficient (mm^{-1})	0.598	0.665
F(000)	956	2028
Crystal Size (mm^3)	0.110 x 0.098 x 0.040	0.146 x 0.137 x 0.057
Crystal color, habit	Purple block	Green block
Final R indices [I>2σ(I)]	R_1 = 0.0360, wR_2 = 0.0843	R_1 = 0.0290, wR_2 = 0.0662
R indices (all data)	R_1 = 0.0417, wR_2 = 0.0879	R_1 = 0.0350, wR_2 = 0.0697

Table S4. Crystal data and structure refinement for **7**.

	**7**●$THF_{0.79}(C_6H_6)_{0.216}$
CCDC Number	2372722
Empirical Formula	$C_{45.44}H_{46.56}CoF_6N_2O_{0.79}P_4$
Molecular Formula	$C_{41}H_{39}CoN_2P_3$, 0.784(C_4H_8O), 0.216(C_6H_6), F_6P
Formula Weight (g/mol)	930.00
Temperature (K)	100.0
Wavelength (Å)	0.71073
Crystal System	Triclinic
Space Group	P-1
a (Å)<br>b (Å)<br>c (Å)<br>α (°)<br>β (°)<br>γ (°)	11.4936(5)<br>12.2636(5)<br>15.7199(7)<br>87.1280(10)<br>75.067(2)<br>85.5970(10)
Volume (Å^3)	2133.56(16)
Z	2
Density (calculated) (g/cm^3)	1.448
Absorption Coefficient (mm^{-1})	0.616
F(000)	961
Crystal Size (mm^3)	0.132 x 0.086 x 0.084
Crystal color, habit	red block
Final R indices [I>2σ(I)]	R_1 = 0.0282, wR_2 = 0.0707
R indices (all data)	R_1 = 0.0313, wR_2 = 0.0734

Magnetic Susceptibility Data

Figure S34. Magnetic susceptibility of **1**, **2**, **5**, and **6** as a function of temperature.

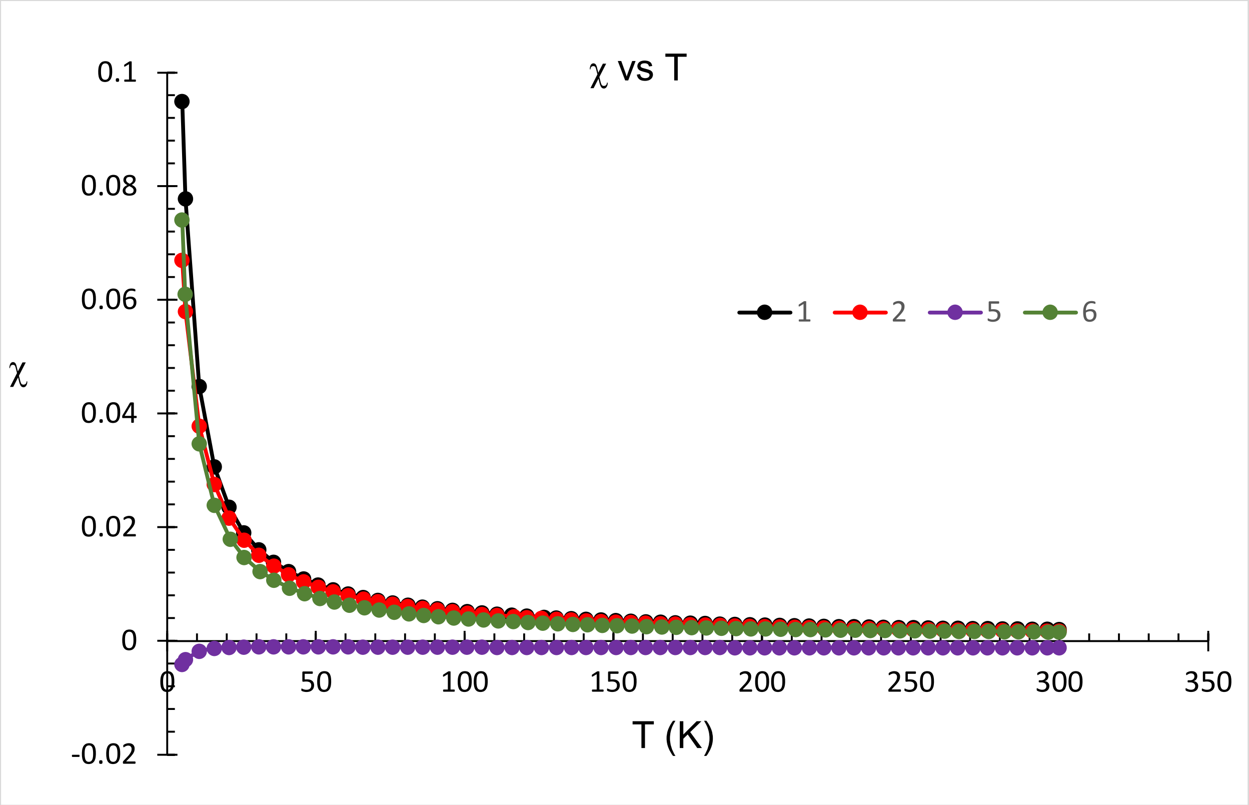

Moment vs Field Strength Graphs. To rule out the presence of other paramagnetic impurities present in the sample, the magnetic moment was measured as a function of field strength.

Figure S35. Moment vs field strength plots for **1**.

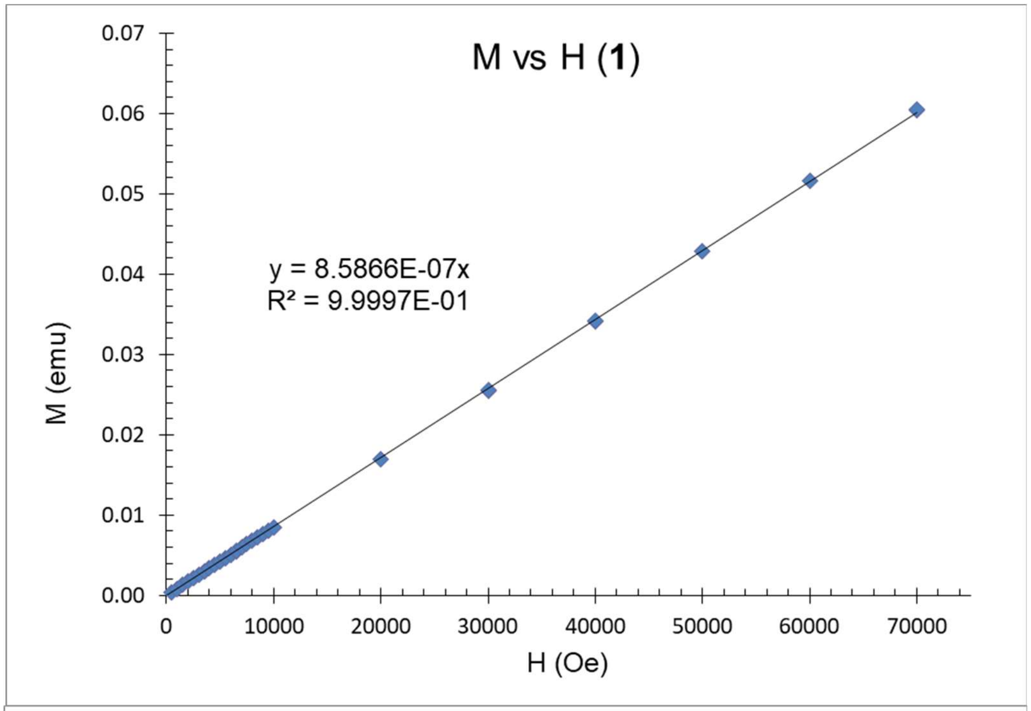

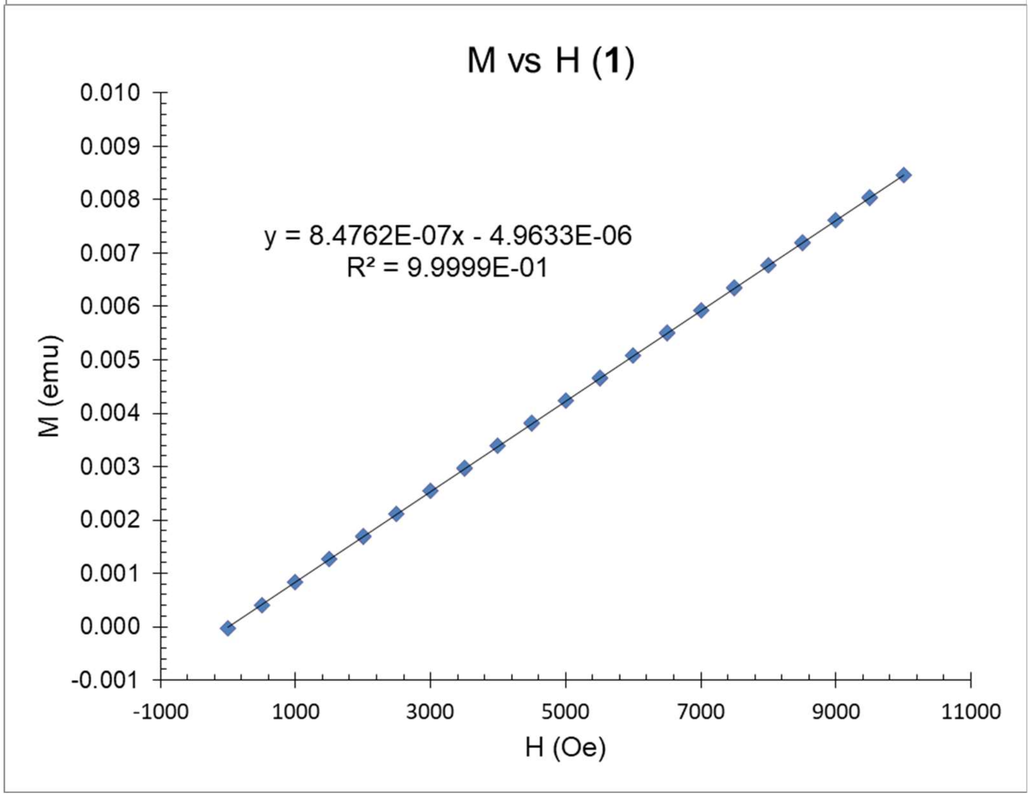

Figure S36. Moment vs field strength plots for **2**.

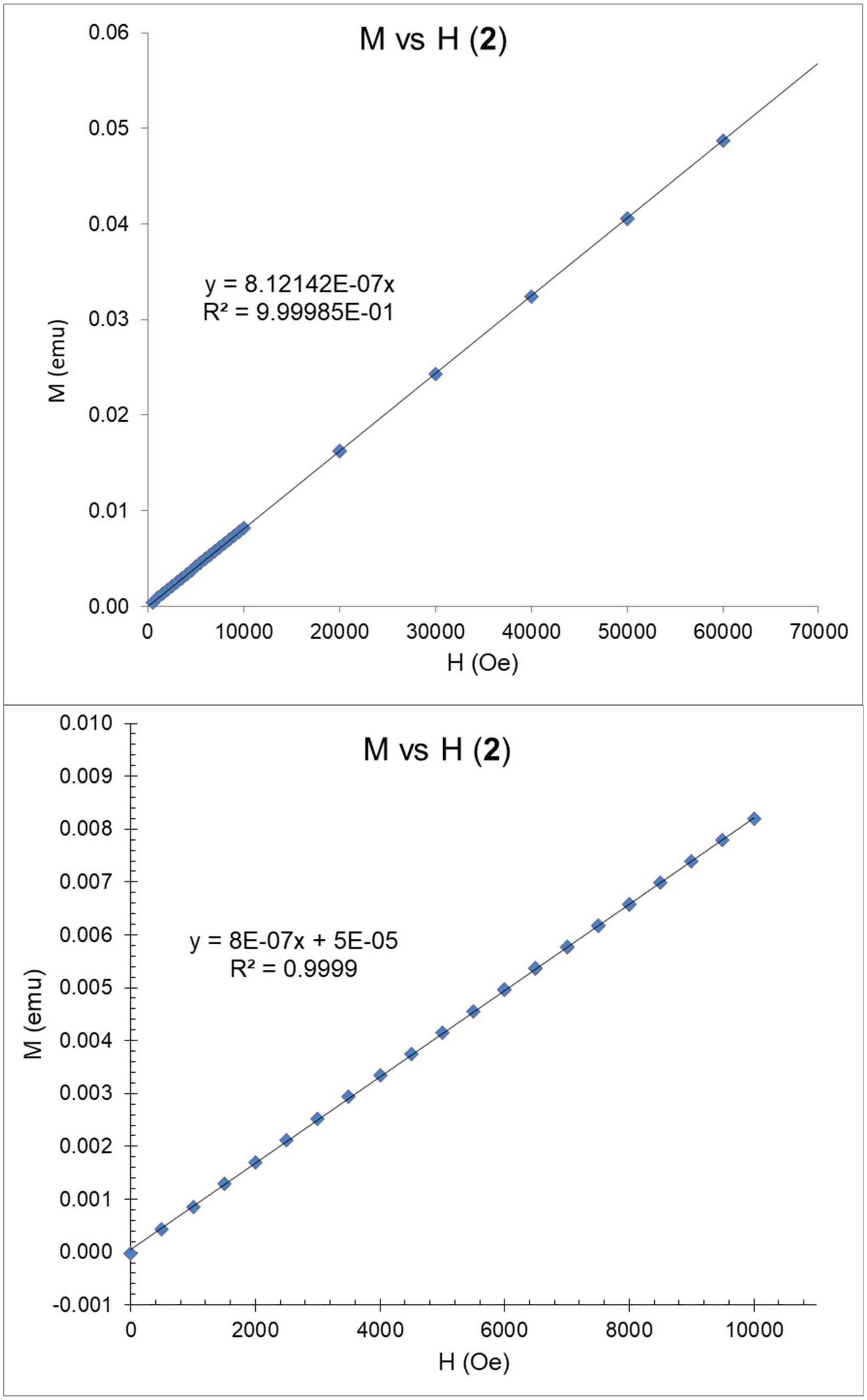

Figure S37. Moment vs field strength plots for **5**.

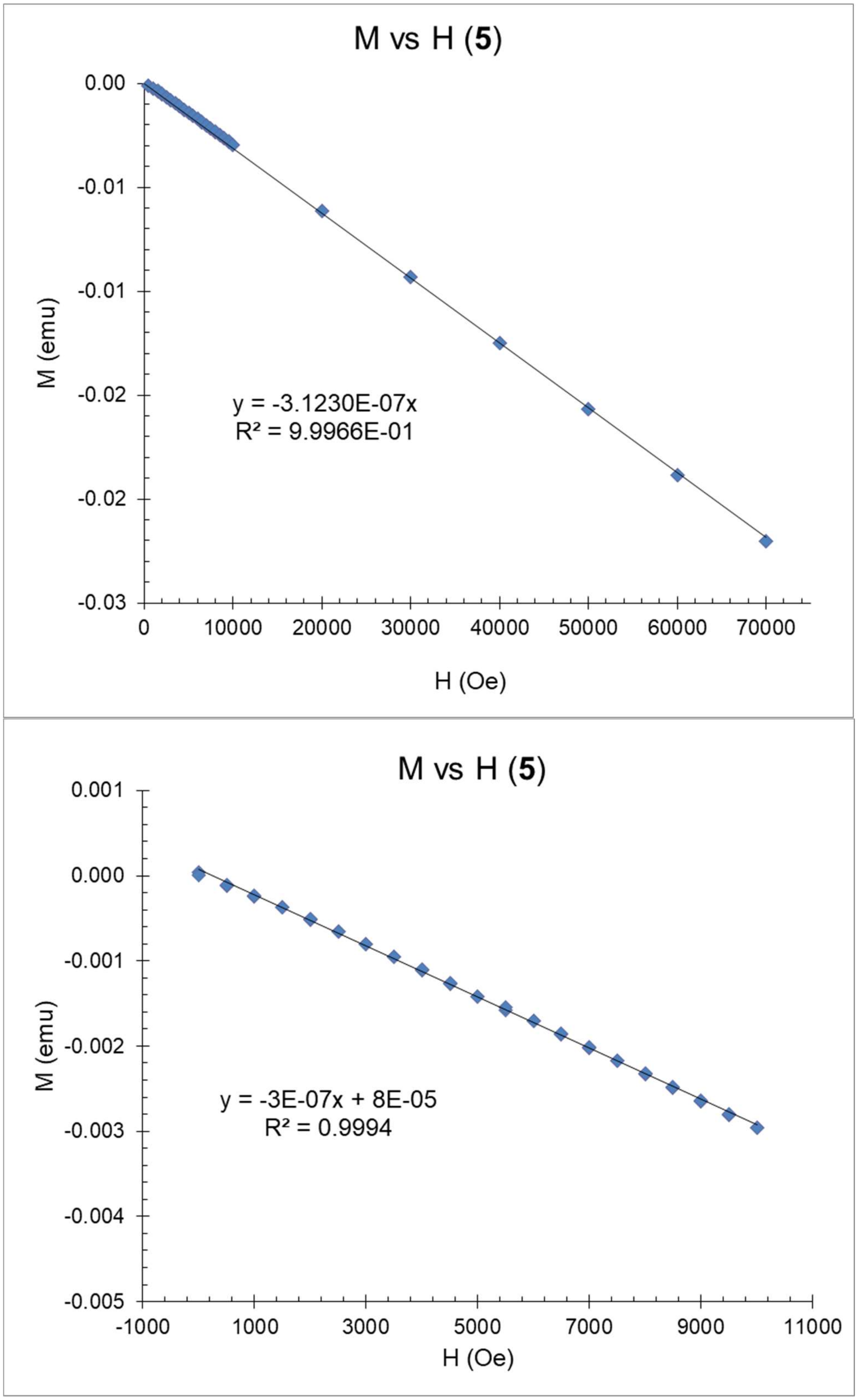

Figure S38. Moment vs field strength plots for **6**.

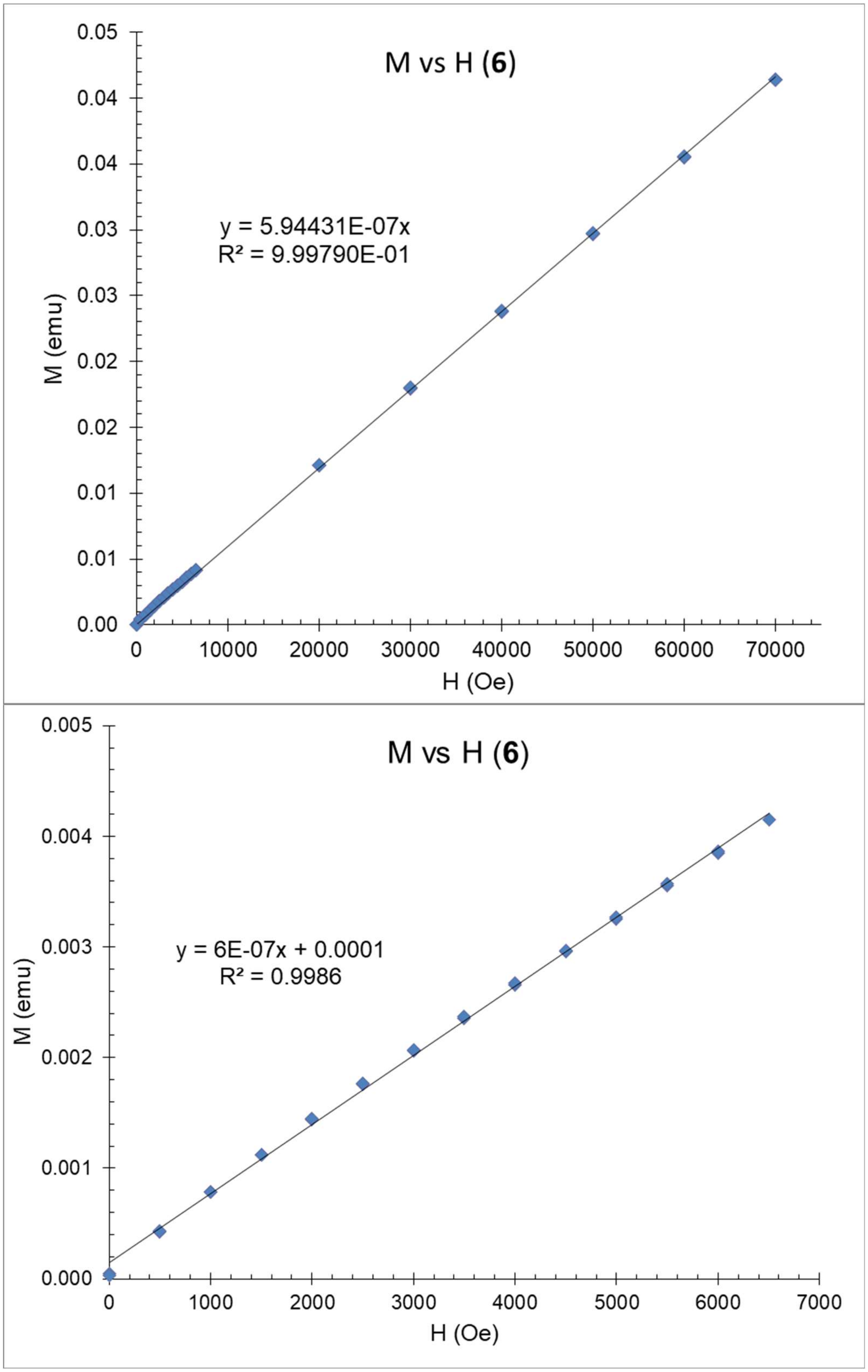

Quantum Chemical Calculations:

Computational Details. The ORCA 5.0.3 software package[5–7] was used for all computations. Geometry optimizations, single-point numerical frequency calculations, and broken-symmetry calculations were performed at the ωB97X-D3[8]/def2-SVP[9,10] level of theory in the gas phase. Crystallographic coordinates were used as the starting geometries for **1** and **2**, following removal of the solvate molecule in the crystal lattice. The starting geometries for $\mathbf{5}^{0}$, $\mathbf{5}^{\mathbf{THF}}$, and $\mathbf{5}^{\mathbf{2THF}}$ in all three spin states were obtained by removing the PF_6 counterion and THF solvate molecule leaving a THF molecule bound to the metal center ($\mathbf{5}^{\mathbf{THF}}$), then removing the remaining THF molecule ($\mathbf{5}^{\mathbf{0}}$) or adding an additional THF molecule to the cobalt center ($\mathbf{5}^{\mathbf{2THF}}$). The starting geometry for **6** was obtained from the crystallographic coordinates following removal of the two PF_6 anions and THF solvate. The starting geometry for **7** was obtained from the crystallographic coordinates following removal of the PF_6 anion, THF solvate, and benzene solvate. Numerical frequency calculations were performed on all optimized geometries to ensure the absence of significant imaginary frequencies (>100 cm^{-1}). When changing the spin-state of a molecule, the same starting geometry was used for each spin-state calculation.

Figure S39. Relative energies of $\mathbf{5}^{\mathbf{THF}}$ and **7** in three different spin states.

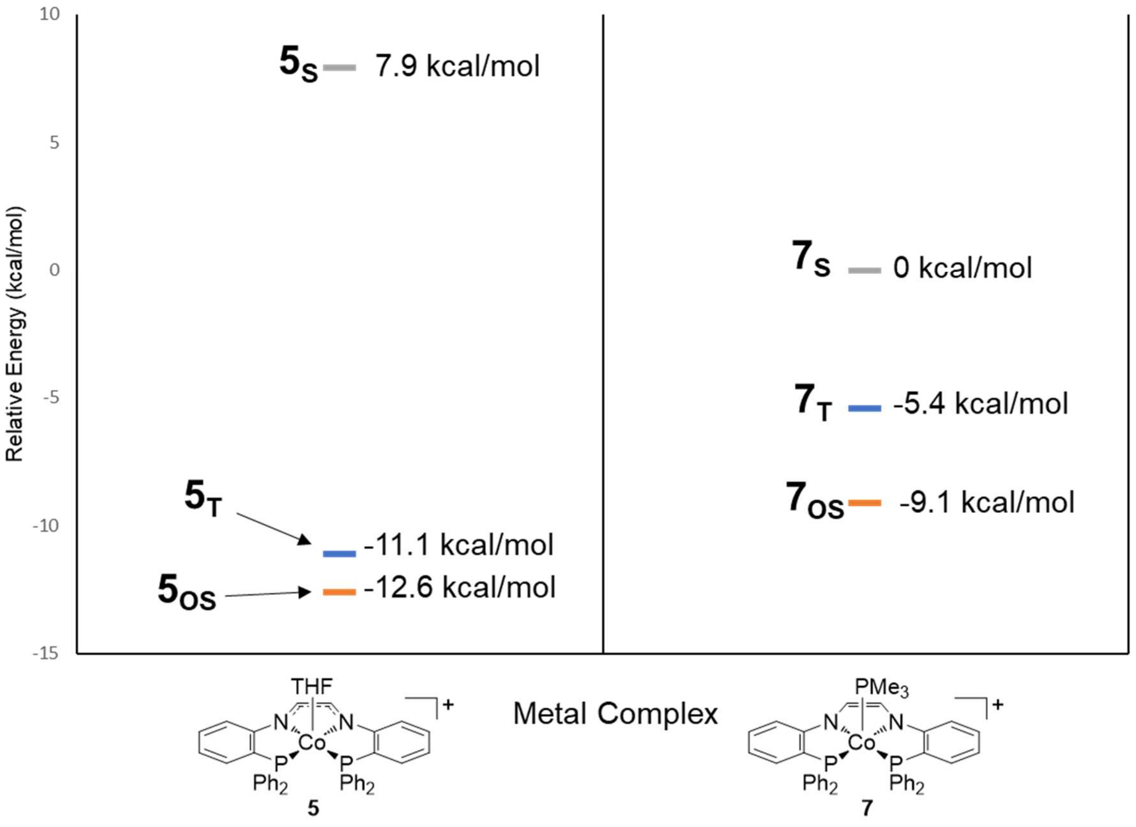

Spin Density Plots

Figure S40. Spin density plot of **1** (isovalue = 0.008).

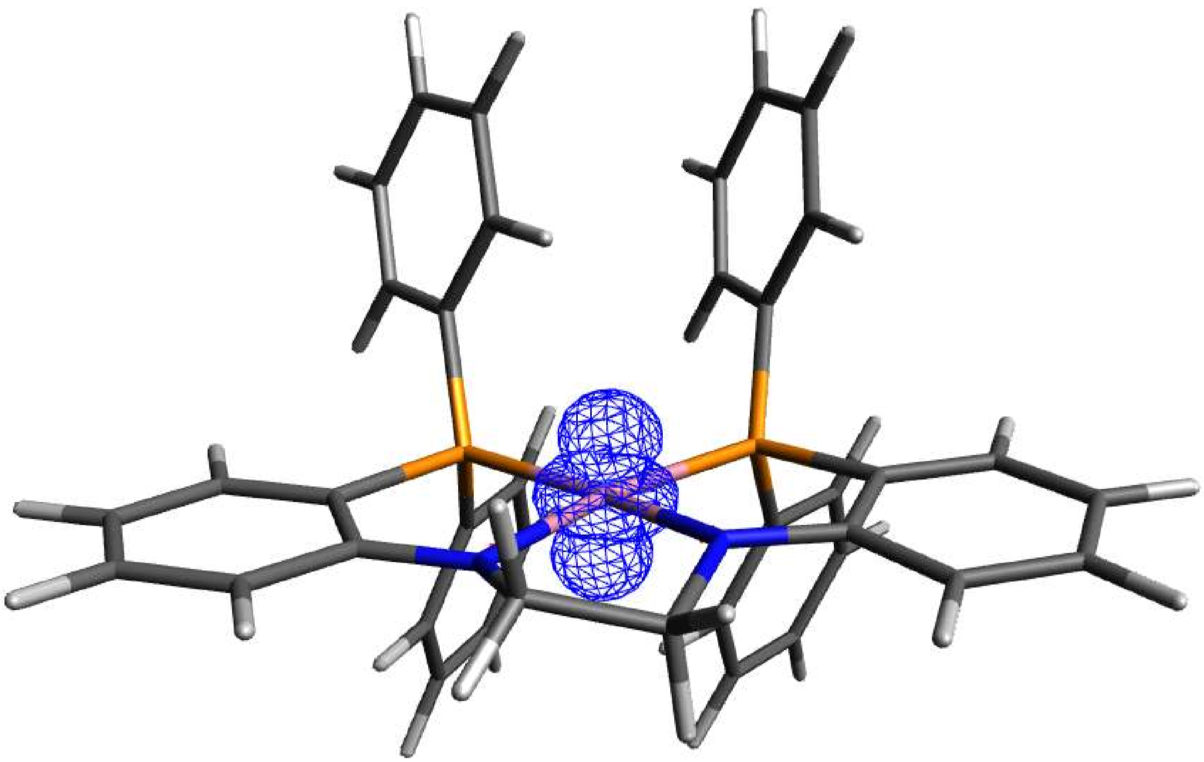

Figure S41. Spin density plot of **2** (isovalue = 0.04).

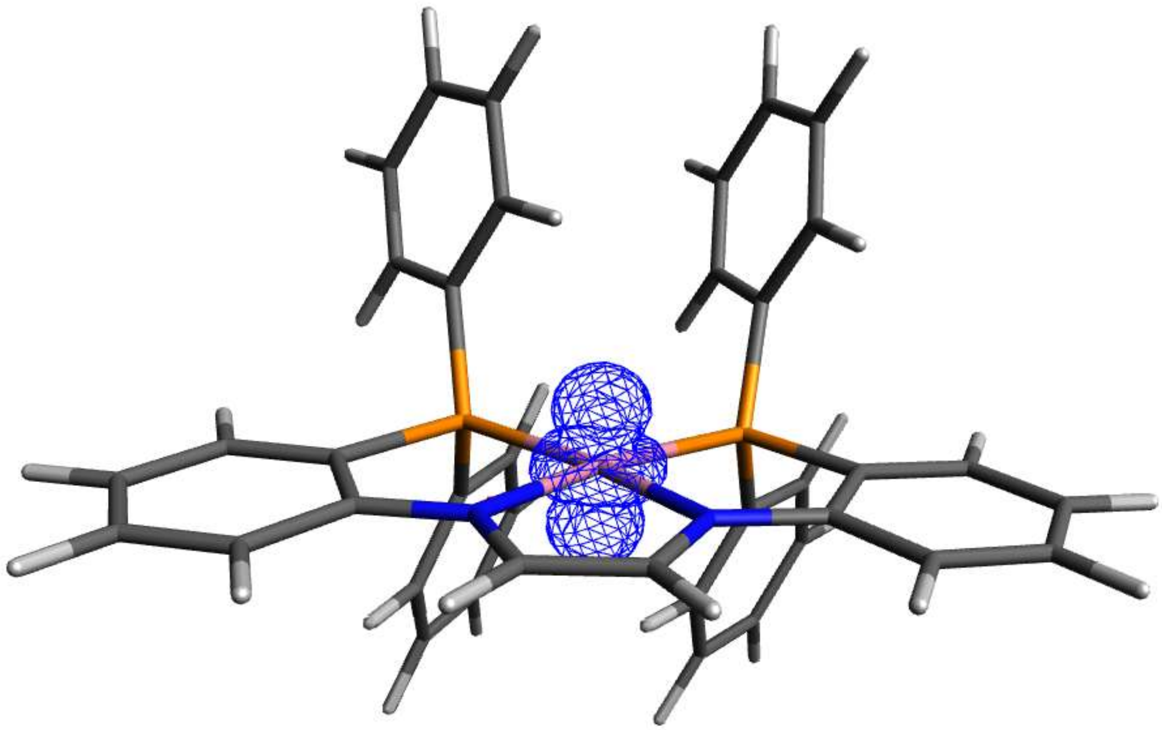

Figure S42. Spin density plot of $\mathbf{5^{0}_{OS}}$ (isovalue = 0.004).

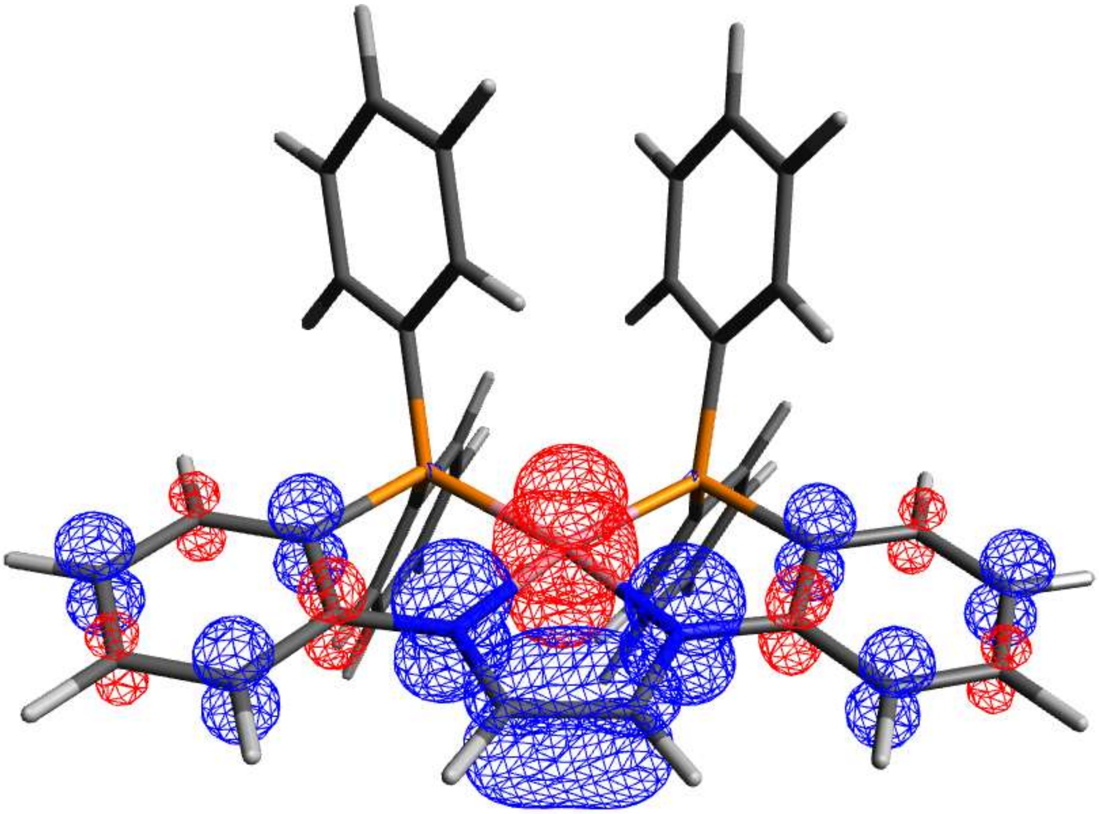

Figure S43. Spin density plot of $\mathbf{5^{THF}_{OS}}$ (isovalue = 0.004).

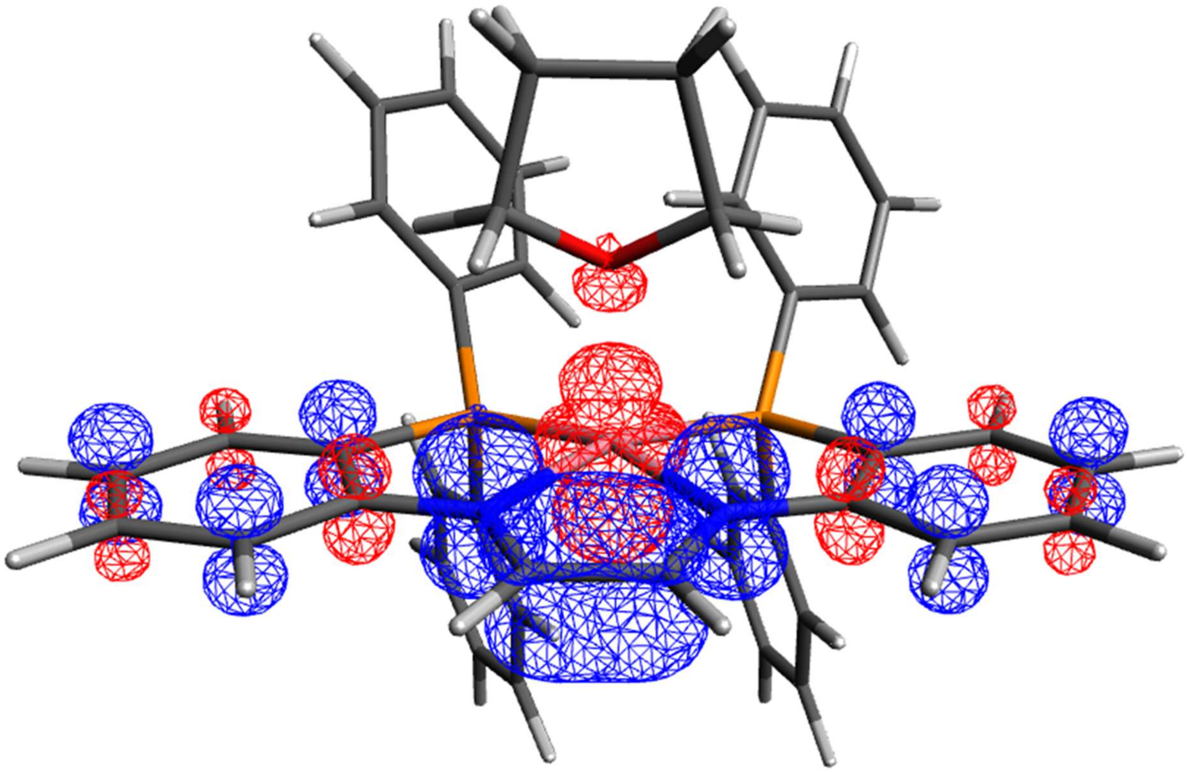

Figure S44. Spin density plot of $\mathbf{5^{2THF}_{OS}}$ (isovalue = 0.004).

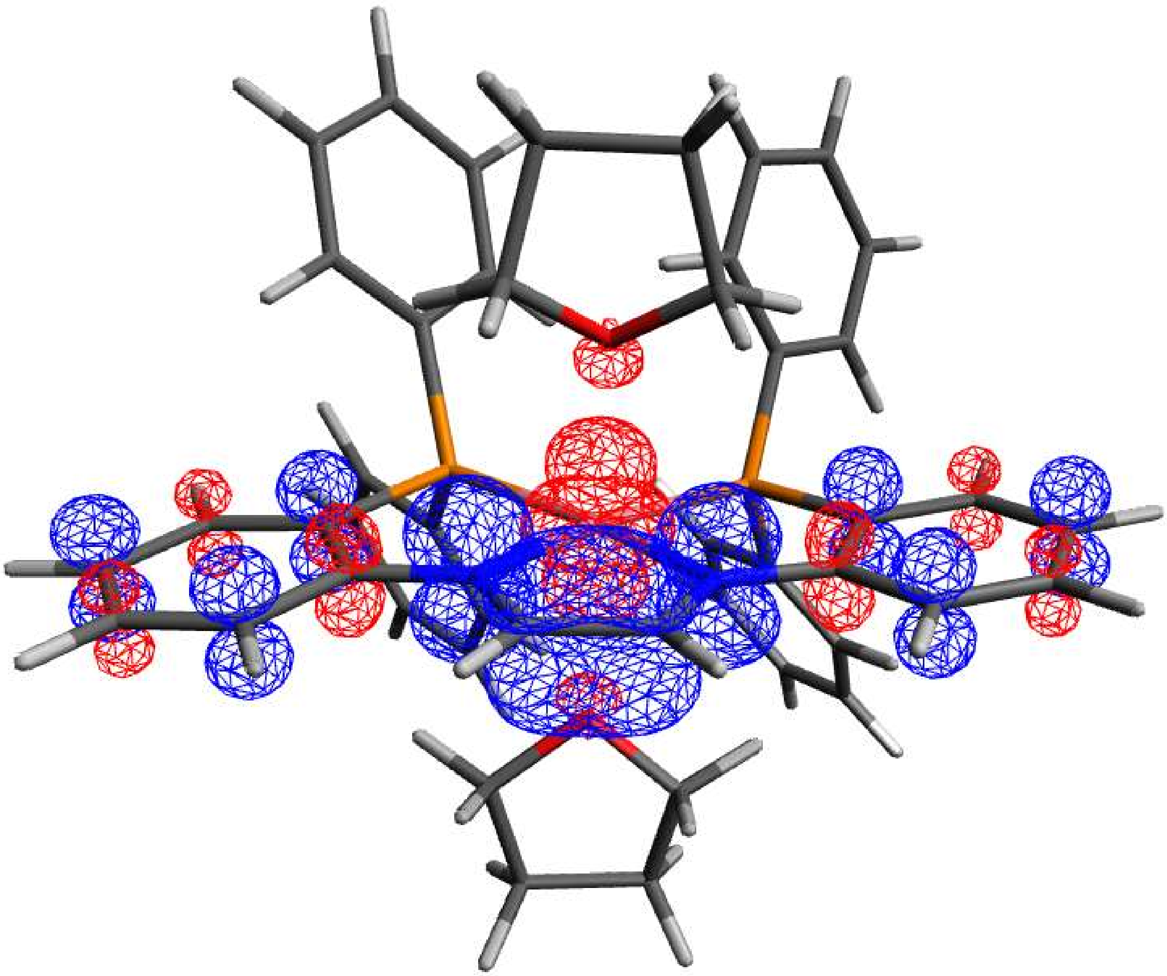

Figure S45. Spin density plot of **6** (isovalue = 0.008).

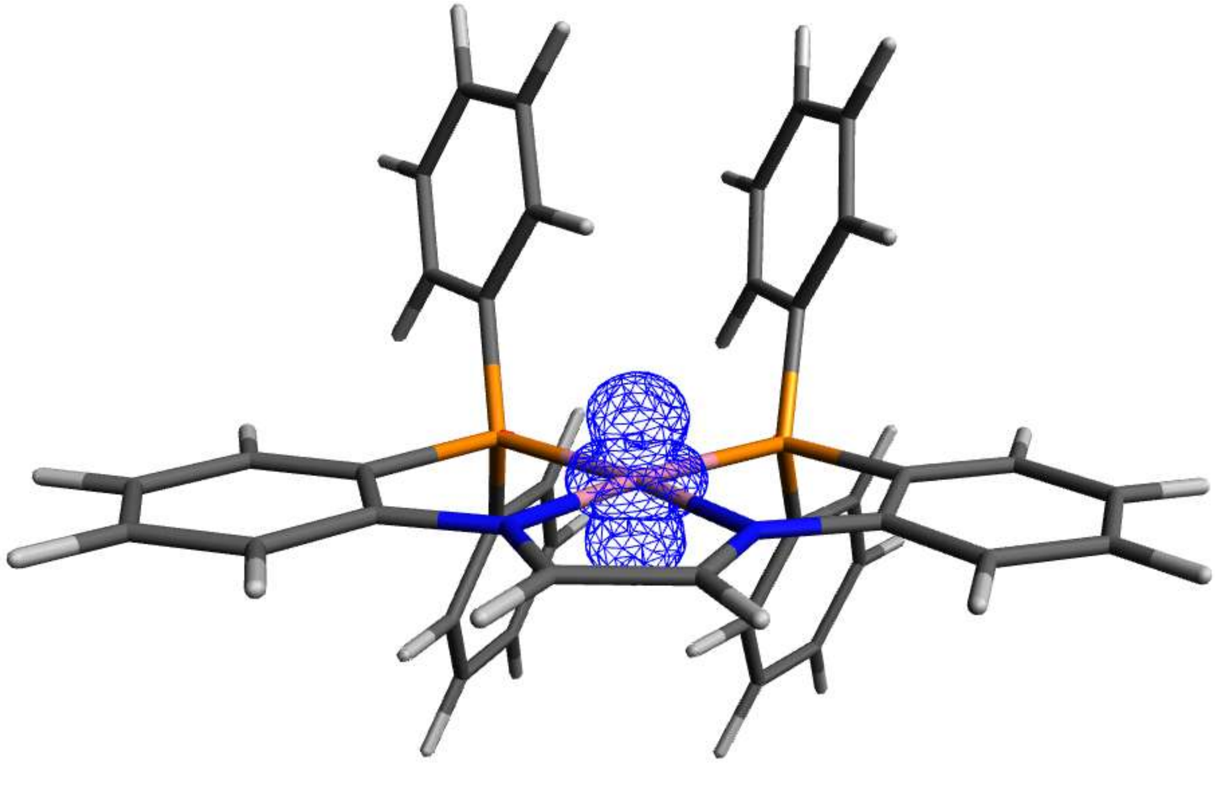

Comparison to X-ray structures. The computed bond distances were compared to the bond distances obtained from the crystal structures for complexes **1**, **2**, **5**, and **6**. The first column describes the atom numbers from the computational data with the corresponding atoms in the crystal structure listed in parentheses. The bonds colored in red represent the internal structure of the molecule (including the backbone), blue represents the heteroatom-carbon connections between the internal structure and the aryl linkers, and green/black represent the C-C bonds in the aryl linkers.

Table S5. Computed bond distances compared to solid-state structure of **1**.

Bond	Computed (Å)	Crystal Structure (Å)	Deviation (Å)
Co1-N4 (Co1-N2)	1.890	1.8754(14)	0.0146
Co1-N5 (Co1-N1)	1.888	1.8669(14)	0.0211
Co1-P3 (Co1-P2)	2.225	2.1932(5)	0.0318
Co1-P2 (Co1-P1)	2.222	2.1930(5)	0.029
N4-C8 (N1-C7)	1.448	1.452(2)	0.004
N5-N26 (N2-C8)	1.448	1.450(2)	0.002
C8-C26 (C7-C8)	1.524	1.522(2)	0.002
N4-C23 (N1-C2)	1.358	1.375(2)	0.017
N5-C22 (N2-C9)	1.357	1.373(2)	0.016
P3-C24 (P1-C1)	1.803	1.8146(16)	0.0116
P2-C33 (P2-C10)	1.803	1.8140(16)	0.011
C24-C54 (C1-C6)	1.396	1.390(2)	0.006
C54-C60 (C6-C5)	1.390	1.386(2)	0.004
C60-C44 (C5-C4)	1.402	1.394(3)	0.008
C44-C48 (C4-C3)	1.387	1.384(2)	0.003
C48-C23 (C3-C2)	1.422	1.414(2)	0.008
C23-C24 (C2-C1)	1.427	1.417(2)	0.01
C33-C52 (C10-C11)	1.396	1.389(2)	0.007
C52-C70 (C11-C12)	1.390	1.393(2)	0.003
C70-C66 (C12-C13)	1.402	1.390(3)	0.012
C66-C16 (C13-C14)	1.387	1.391(2)	0.004
C16-C22 (C14-C9)	1.422	1.413(2)	0.009
C22-C33 (C9-C10)	1.427	1.419(2)	0.008
		**Average Deviation:**	**0.011 Å**

Table S6. Computed bond distances compared to solid-state structure of **2**.

Bond	Computed (Å)	Crystal Structure (Å)	Deviation (Å)
Co-N3 (Co1-N1)	1.892	1.878(2)	0.014
Co-N39 (Co1-N1)	1.893	1.878(2)	0.015
Co-P2 (Co1-P1)	2.218	2.1866(7)	0.0314
Co-P38 (Co1-P1)	2.220	2.1866(7)	0.0334
N3-C14 (N1-C7)	1.395	1.403(3)	0.008
N39-C58 (N1-C7)	1.395	1.403(3)	0.008
C14-C58 (C7-C7)	1.349	1.396(5)	0.047
N3-C23 (N1-C2)	1.364	1.380(3)	0.016
N39-C49 (N1-C2)	1.363	1.380(3)	0.017
P2-C13 (P1-C1)	1.806	1.810(3)	0.004
P38-C49 (P1-C1)	1.807	1.810(3)	0.003
C13-C4 (C1-C6)	1.396	1.389(3)	0.007
C4-C18 (C6-C5)	1.390	1.391(4)	0.001
C18-C9 (C5-C4)	1.402	1.383(4)	0.019
C9-C32 (C4-C3)	1.386	1.388(4)	0.002
C32-C23 (C3-C2)	1.419	1.410(3)	0.009
C23-C13 (C2-C1)	1.425	1.417(4)	0.008
C49-C40 (C1-C6)	1.396	1.389(3)	0.007
C40-C54 (C6-C5)	1.390	1.391(4)	0.001
C54-C45 (C5-C4)	1.402	1.383(4)	0.019
C45-C68 (C4-C3)	1.386	1.388(4)	0.002
C68-C59 (C3-C2)	1.419	1.410(3)	0.009
C59-C49 (C2-C1)	1.425	1.417(4)	0.008
		**Average Deviation:**	**0.013 Å**

Table S7. Computed bond distances of 5^{THF}_{S}, 5^{THF}_{OS} and 5^{THF}_{T} compared to solid-state structure of **5**.

Bond Calculated (Crystal)	Crystal Structure (Å)	5^{THF}_{S}		5^{THF}_{OS}		5^{THF}_{T}	
		Computed (Å)	Deviation (Å)	Computed (Å)	Deviation (Å)	Computed (Å)	Deviation (Å)
Co-N5 (Co1-N2)	1.906(3)	1.878	0.028	1.932	0.026	1.934	0.028
Co-N48 (Co1-N1)	1.910(3)	1.877	0.033	1.933	0.023	1.935	0.025
Co-P3 (Co1-P2)	2.1835(11)	2.224	0.0405	2.228	0.0445	2.232	0.0485
Co-P2 (Co1-P1)	2.1997(10)	2.223	0.0233	2.231	0.0313	2.234	0.0343
N5-C44 (N2-C8)	1.337(5)	1.342	0.005	1.337	0	1.337	0
N48-C77 (N1-C7)	1.336(5)	1.342	0.006	1.336	0	1.337	0.001
C44-C77 (C8-C7)	1.391(6)	1.386	0.005	1.399	0.008	1.399	0.008
N5-C12 (N2-C9)	1.408(5)	1.411	0.003	1.398	0.01	1.394	0.014
N48-C16 (N1-C2)	1.404(5)	1.410	0.006	1.398	0.006	1.394	0.01
P3-C13 (P2-C10)	1.822(4)	1.820	0.002	1.823	0.001	1.821	0.001
P2-C82 (P1-C1)	1.819(4)	1.825	0.006	1.828	0.009	1.827	0.008
C13-C71 (C10-C11)	1.401(6)	1.395	0.006	1.396	0.005	1.396	0.005
C71-C38 (C11-C12)	1.388(6)	1.391	0.003	1.391	0.003	1.391	0.003
C38-C46 (C12-C13)	1.384(6)	1.396	0.012	1.397	0.013	1.397	0.013
C46-C51 (C13-C14)	1.389(6)	1.391	0.002	1.390	0.001	1.389	0
C51-C12 (C14-C9)	1.405(6)	1.401	0.004	1.404	0.001	1.405	0
C12-C13 (C9-C10)	1.396(5)	1.407	0.011	1.412	0.016	1.412	0.016
C82-C10 (C1-C6)	1.396(6)	1.395	0.001	1.395	0.001	1.395	0.001
C10-C6 (C6-C5)	1.390(6)	1.391	0.001	1.392	0.002	1.391	0.001
C6-C42 (C5-C4)	1.392(6)	1.396	0.004	1.397	0.005	1.397	0.005
C42-C20 (C4-C3)	1.379(6)	1.391	0.012	1.390	0.011	1.389	0.01
C20-C16 (C3-C2)	1.405(5)	1.401	0.004	1.404	0.001	1.405	0
C16-C82 (C2-C1)	1.403(6)	1.407	0.004	1.412	0.009	1.413	0.01
**Average Deviation:**			**0.0096 Å**		**0.0099 Å**		**0.011 Å**

Table S8 Computed bond distances of **6** (S = 1/2) and **6** (S = 3/2) compared to solid-state structure of **6**.

Bond Calculation (Crystal)	Crystal Structure (Å)	**6** (S =1/2)		**6** (S = 3/2)	
		Computed (Å)	Deviation (Å)	Computed (Å)	Deviation (Å)
Co-N3 (Co1-N1)	1.935(3)	1.953	0.018	2.100	0.165
Co-N31 (Co1-N2)	1.924(3)	1.953	0.029	2.119	0.195
Co-P2 (Co1-P1)	2.2053(10)	2.221	0.0157	2.343	0.1377
Co-P30 (Co1-P2)	2.2101(10)	2.218	0.0079	2.334	0.1239
N3-C10 (N1-C7)	1.289(5)	1.289	0	1.282	0.007
N31-C42 (N2-C8)	1.286(5)	1.289	0.003	1.281	0.005
C10-C42 (C7-C8)	1.450(5)	1.468	0.018	1.479	0.029
N3-C17 (N1-C2)	1.420(5)	1.417	0.003	1.418	0.002
N31-C51 (N2-C9)	1.426(5)	1.417	0.009	1.418	0.008
P2-C9 (P1-C1)	1.824(4)	1.831	0.007	1.832	0.008
P30-C41 (P2-C10)	1.820(4)	1.830	0.01	1.826	0.006
C9-C67 (C1-C6)	1.398(5)	1.396	0.002	1.396	0.002
C67-C68 (C6-C5)	1.381(5)	1.393	0.012	1.393	0.012
C67-C69 (C5-C4)	1.390(6)	1.398	0.008	1.395	0.005
C69-C66 (C4-C3)	1.385(5)	1.389	0.004	1.390	0.005
C66-C17 (C3-C2)	1.394(5)	1.400	0.006	1.400	0.006
C17-C9 (C2-C1)	1.395(5)	1.409	0.014	1.414	0.019
C41-C32 (C10-C11)	1.400(5)	1.396	0.004	1.395	0.005
C32-C46 (C11-C12)	1.382(5)	1.393	0.011	1.394	0.012
C46-C37 (C12-C13)	1.376(6)	1.398	0.022	1.395	0.019
C37-C60 (C13-C14)	1.381(6)	1.389	0.008	1.391	0.01
C60-C51 (C14-C9)	1.393(5)	1.400	0.007	1.399	0.006
C51-C41 (C9-C10)	1.393(5)	1.409	0.016	1.413	0.02
**Average Deviation:**			**0.010 Å**		**0.035 Å**

Table S9. Computed bond distances of 7_S, 7_T, and 7_{OS} compared to solid state structure.

Bond Calculated (Crystal)	Crystal Structure (Å)	7_S Computed (Å)	7_S Deviation (Å)	7_T Computed (Å)	7_T Deviation (Å)	7_{OS} Computed (Å)	7_{OS} Deviation (Å)
Co-N5 (Co1-N1)	1.8825(13)	1.862	0.0205	1.950	0.0675	1.928	0.0455
Co-N6 (Co1-N2)	1.8903(13)	1.879	0.0113	1.944	0.0537	1.924	0.0337
Co-P3 (Co1-P2)	2.1938(4)	2.232	0.0382	2.239	0.0452	2.227	0.0332
Co-P2 (Co1-P3)	2.1856(4)	2.209	0.0234	2.243	0.0574	2.228	0.0424
Co-P4 (Co1-P1)	2.2344(4)	2.231	0.0034	2.447	0.2126	2.350	0.1156
N5-C29 (N1-C1)	1.352(2)	1.355	0.003	1.339	0.013	1.341	0.011
N6-C23 (N2-C2)	1.348(2)	1.349	0.001	1.336	0.012	1.338	0.01
C23-C29 (C1-C2)	1.378(2)	1.378	0	1.398	0.02	1.394	0.016
N5-C13 (N1-C3)	1.414(2)	1.414	0	1.392	0.022	1.400	0.014
N6-C7 (N2-C9)	1.413(2)	1.409	0.004	1.394	0.019	1.401	0.012
P3-C14 (P2-C4)	1.8156(16)	1.818	0.0024	1.820	0.0044	1.821	0.0054
P2-C17 (P3-C10)	1.8161(15)	1.820	0.0039	2.243	0.4269	1.825	0.0089
C14-C27 (C4-C5)	1.398(2)	1.396	0.002	1.399	0.001	1.397	0.001
C27-C15 (C5-C6)	1.387(2)	1.391	0.004	1.390	0.003	1.391	0.004
C15-C38 (C6-C7)	1.390(3)	1.396	0.006	1.399	0.009	1.397	0.007
C38-C31 (C7-C8)	1.388(3)	1.392	0.004	1.388	0	1.390	0.002
C31-C13 (C8-C3)	1.399(2)	1.402	0.003	1.407	0.008	1.404	0.005
C13-C14 (C3-C4)	1.400(2)	1.406	0.006	1.412	0.012	1.410	0.01
C17-C11 (C10-C11)	1.391(2)	1.394	0.003	1.395	0.004	1.394	0.003
C11-C8 (C11-C12)	1.391(2)	1.393	0.002	1.392	0.001	1.393	0.002
C8-C45 (C12-C13)	1.388(2)	1.396	0.008	1.397	0.009	1.396	0.008
C45-C25 (C13-C14)	1.388(2)	1.392	0.004	1.390	0.002	1.392	0.004
C25-C7 (C14-C9)	1.398(2)	1.400	0.002	1.404	0.006	1.402	0.004
C7-C17 (C9-C10)	1.404(2)	1.408	0.004	1.412	0.008	1.410	0.006
**Average Deviation:**			**0.0066 Å**		**0.042 Å**		**0.017 Å**

XYZ coordinates of optimized structures:

Table S10. XYZ coordinates of DFT optimized geometry of **1**.

Symbol	X	Y	Z
Co	7.880339795	6.177502106	11.17171233
P	6.308577218	7.729089813	10.9305637
P	6.726404922	4.309802119	11.53255894
N	9.404023935	5.059718859	11.2179859
N	9.101997205	7.603720553	10.97400097
C	5.699562192	6.748660863	8.418782415
H	6.61741567	6.16875958	8.552221488
C	10.64969348	5.789069271	11.32952124
H	11.48565711	5.276372009	10.81862243
H	10.94716457	5.890736276	12.39472952
C	6.217381144	3.051719981	9.092627425
H	7.269219781	2.754997613	9.138393474
C	5.17115964	8.027739466	12.32976413
C	4.300376002	4.119757036	10.09794071
H	3.841858891	4.663634898	10.92767156
C	9.610246147	9.952604179	10.41374438
H	10.68359456	9.769833054	10.3477612
C	3.941031644	7.365275734	12.42454809
H	3.591714885	6.743322686	11.59636818

C	5.603977982	8.816075698	13.40283518
H	6.563237358	9.337542399	13.33703082
C	8.724252134	8.880494189	10.71069577
C	9.352223012	3.745279432	11.55429832
C	8.07477315	3.123603288	11.68993365
C	5.647335318	3.742563428	10.16839791
C	10.45849798	7.170534453	10.71395235
H	11.2100891	7.863193413	11.1352237
H	10.65100721	7.124365304	9.621278263
C	5.709921912	4.195081903	13.04706509
C	5.25653319	7.593940002	9.442127835
C	4.976672844	6.630568792	7.234677048
H	5.331762913	5.959803799	6.449033072
C	7.332853852	9.19868335	10.72929599
C	3.593839829	8.276707719	14.63250684
H	2.980182105	8.370643628	15.53185696
C	3.534275828	3.803760248	8.979204479
H	2.485860241	4.108332443	8.937334991
C	4.074712894	8.322711041	9.262472238
H	3.715697377	8.989179071	10.05166377
C	3.346859724	8.197645403	8.081000818
H	2.423383926	8.767085623	7.95060533
C	3.155777953	7.491399786	13.5672301
H	2.200806544	6.964286996	13.62872976
C	10.32787464	1.570727703	12.08403355
H	11.22267768	0.9644666	12.25438945
C	3.79649639	7.351967118	7.066697754
H	3.222998726	7.256912042	6.141212583
C	10.4838413	2.916685154	11.78940559
H	11.48786527	3.339427746	11.73480749
C	4.817806036	8.938955808	14.54737775
H	5.16489447	9.559910333	15.37699016
C	6.868748848	10.4983138	10.52148524
H	5.791924805	10.69561555	10.54552637
C	7.94131732	1.766863594	11.98865188
H	6.94088568	1.332343809	12.08531163
C	4.715425443	3.226208917	13.22995924
H	4.495463341	2.507857173	12.43531528
C	5.45030055	2.740479313	7.970802681
H	5.906708557	2.199934684	7.137950975
C	9.064675627	0.970829488	12.17803286
H	8.964755027	-0.090447265	12.41319414

C	5.974052222	5.104755564	14.07751959
H	6.741367754	5.872271105	13.94106832
C	4.10884001	3.114831341	7.911917611
H	3.509074564	2.869341718	7.031914637
C	9.127145985	11.23688989	10.21442505
H	9.841585377	12.03686046	9.997776237
C	3.991541854	3.179257214	14.41963863
H	3.214546552	2.422280543	14.55237355
C	7.759172016	11.53632609	10.27540164
H	7.400666314	12.55454118	10.1136138
C	5.255422045	5.052392621	15.26912301
H	5.465765102	5.776491105	16.0594072
C	4.259094781	4.092713755	15.43910172
H	3.68751254	4.056195341	16.36991003

Table S11. XYZ coordinates of DFT optimized geometry of **2**.

Symbol	X	Y	Z
Co	1.200165467	4.016440883	11.23061012
P	0.570172064	5.291700098	9.529386753
N	0.653608406	2.573625339	10.13532559
C	-0.48136194	4.276962845	7.074590121
H	-0.560596908	5.307207774	6.712767994
C	1.869155021	6.295481838	8.727306321
C	-3.0931374	7.978268213	10.37946088
H	-3.966097728	8.597923062	10.59946413
C	-0.780048901	1.918231334	6.769090122
H	-1.100154876	1.075801758	6.148752489
C	-1.153633156	7.506815625	9.016164437
H	-0.514878461	7.758826589	8.165130596
C	0.032785483	4.035153719	8.349219018
C	0.810854808	1.350555768	10.78727132
H	0.554344518	0.409233457	10.29779162
C	2.140499432	7.602120579	9.152993848
H	1.488626491	8.083697475	9.886359658
C	-0.889534889	3.222321639	6.266699916
H	-1.291358269	3.40470583	5.268334263
C	-0.845838537	6.406258847	9.823998363
C	-2.800247252	6.875314685	11.1797904
H	-3.436460009	6.62647472	12.03209234
C	0.148155829	2.716607321	8.876987343
C	2.72179266	5.693333963	7.793807345
H	2.518470608	4.673104117	7.456010794

C	-1.678942121	6.097963879	10.90621902
H	-1.441944046	5.244585794	11.54831243
C	-2.270590449	8.291785554	9.297891889
H	-2.501243564	9.152238389	8.664905713
C	3.237998038	8.296421665	8.651369115
H	3.439427605	9.31322933	8.996409222
C	-0.278883315	1.657264626	8.034789036
H	-0.217904806	0.62539943	8.382664744
C	4.084254418	7.689498394	7.724720572
H	4.949466042	8.231412976	7.335099197
C	3.823765085	6.388051893	7.297730195
H	4.482258977	5.907327418	6.570129979
P	1.911344895	5.448026801	12.77043507
N	1.572508644	2.68958581	12.52889006
C	2.779692654	4.679065005	15.38073673
H	2.959987188	5.735702721	15.60297793
C	0.683423262	6.648005241	13.40202839
C	5.790561202	7.799867002	11.87317433
H	6.714910941	8.340432189	11.6544455
C	2.790228395	2.370594551	16.02665326
H	2.983295518	1.597097751	16.77600131
C	3.78522463	7.540329843	13.19432188
H	3.141725352	7.882821136	14.00942349
C	2.291445952	4.314717	14.12538581
C	1.29589969	1.411443544	12.04454546
H	1.47218562	0.522731969	12.65339474
C	0.550829552	7.9179575	12.82603855
H	1.261518995	8.24779709	12.06390472
C	3.03307604	3.713284906	16.34753701
H	3.412568321	3.994416027	17.33163786
C	3.417112429	6.42538699	12.43061267
C	5.434959729	6.687035689	11.11338708
H	6.073314135	6.353320078	10.29223258
C	2.045591705	2.954722159	13.77993578
C	-0.237855086	6.242826448	14.37476947
H	-0.143022099	5.25346616	14.83127106
C	4.251807669	6.005231607	11.38871805
H	3.969306558	5.142967043	10.77755596
C	4.964708415	8.225644378	12.91357719
H	5.24165641	9.095728877	13.51393197
C	-0.475728367	8.771271714	13.22054939
H	-0.56740282	9.756947701	12.75817931

C	2.31099311	1.986922698	14.78378868
H	2.136256374	0.92955848	14.58105485
C	-1.388250252	8.361562534	14.19170582
H	-2.196248451	9.029729433	14.49965275
C	-1.266978429	7.097613613	14.76696843
H	-1.977791026	6.772148017	15.53046989

Table S12. XYZ coordinates of DFT optimized geometry of $\mathbf{5^0}_\mathbf{S}$.

Symbol	X	Y	Z
Co	1.498713729	4.140233212	10.25176723
P	-0.065328918	5.512525505	9.558651025
N	0.55106849	2.76161218	9.37569697
C	0.434207179	6.897959934	8.484415574
C	-3.077793676	7.072570567	12.68337785
H	-3.792094641	7.423432166	13.43199708
C	-2.124736164	7.236143896	10.4699475
H	-2.092169365	7.723281502	9.491342916
C	-1.018891476	4.357246701	8.514961014
C	1.053316354	1.563355367	9.552433614
H	0.625439322	0.656281481	9.117367155
C	0.681945502	8.162881022	9.033406308
H	0.447764386	8.361057057	10.08236129
C	-1.25001877	6.183132896	10.76764987
C	-2.215312253	6.019446821	12.98237297
H	-2.245636196	5.542988489	13.96465307
C	-0.585396796	3.020441318	8.569830317
C	0.744810113	6.663510721	7.139299775
H	0.567025941	5.679062905	6.696924603
C	-1.302065432	5.577427642	12.02865137
H	-0.614376067	4.761451892	12.26761639
C	-3.032784609	7.679526052	11.42782767
H	-3.710713257	8.503034124	11.19232889
C	1.221011513	9.178665357	8.248839749
H	1.408834352	10.16052698	8.688975134
C	1.521048914	8.940618622	6.908310463
H	1.941956824	9.738441135	6.292097117
C	1.281307407	7.683531528	6.355201111
H	1.5117432	7.493195475	5.304415203
P	2.774087545	5.45130054	11.45769704
N	2.591651288	2.709055097	10.81586935
C	5.072611862	4.500390697	12.88097492
H	5.261707217	5.528219475	13.20128764

C	2.019521217	6.206923075	12.93537973
C	5.223649728	8.663555598	9.233971309
H	5.799923768	9.414175386	8.687844364
C	5.685310596	2.168737656	12.85184603
H	6.360653003	1.365331075	13.15420511
C	4.371695537	7.798179857	11.32284819
H	4.277736527	7.88093514	12.40927439
C	3.97002203	4.216879685	12.07108778
C	2.212397756	1.532814594	10.37620825
H	2.728249969	0.600118525	10.61900619
C	1.480717774	7.49809965	12.87151373
H	1.625699211	8.108925513	11.97687429
C	5.929341492	3.479201068	13.27414886
H	6.791935613	3.69972248	13.90624466
C	3.740303797	6.754243162	10.63408297
C	4.602960489	7.62372589	8.544004133
H	4.686947321	7.555395904	7.457256197
C	3.725641724	2.899429978	11.64495246
C	1.825329917	5.439940719	14.09027295
H	2.237720182	4.428976732	14.15443199
C	3.862065021	6.673572966	9.241647884
H	3.360304698	5.867467236	8.698445843
C	5.108756518	8.749497494	10.6220005
H	5.596522855	9.564138115	11.16223752
C	0.76420357	8.015884795	13.94663758
H	0.347565395	9.023594965	13.88463524
C	4.595073277	1.873904928	12.04371898
H	4.436354219	0.843032749	11.72396404
C	0.575111075	7.247770236	15.09450848
H	0.012968205	7.654504597	15.93838203
C	1.10918644	5.961912897	15.16588203
H	0.970422006	5.359077239	16.06632012
C	-1.273501015	2.041378999	7.838775066
C	-2.128767047	4.703472185	7.739426596
C	-2.804969516	3.728270051	7.016219633
C	-2.373020368	2.399524768	7.070174588
H	-2.464425136	5.743262667	7.70401114
H	-3.671976858	3.99765538	6.409544563
H	-2.903176358	1.631309173	6.502930666
H	-0.957713169	0.9973913	7.858600742

Table S13. XYZ coordinates of DFT optimized geometry of $\mathbf{5^0_{OS}}$.

Symbol	X	Y	Z
Co	1.186317429	4.033085946	11.23286637
P	0.574669948	5.322526731	9.533327333
P	0.626404738	2.559499446	10.14046648
O	-0.469351356	4.300937879	7.081352593
N	-0.534404541	5.330772202	6.71933772
C	1.890749291	6.290813942	8.734514822
H	-3.106913914	7.964921897	10.36768097
C	-3.987988617	8.575238892	10.57945493
H	-0.806502589	1.940221641	6.751918796
C	-1.137248705	1.110864059	6.122308076
H	-1.148732959	7.519562159	9.022625355
C	-0.503236963	7.783094262	8.180354376
C	0.034953426	4.04337577	8.357497451
C	0.783644576	1.387800675	10.76541031
H	0.523365121	0.434312367	10.29845972
C	2.158640692	7.603305526	9.145368291
C	1.498693345	8.102161821	9.859578587
C	-0.888681449	3.250509232	6.271992156
H	-1.282828999	3.447632682	5.273360274
C	-0.845901868	6.4161833	9.829287155
H	-2.816531022	6.859867571	11.1664871
C	-3.463583652	6.601111403	12.00724849
H	0.117844674	2.721511706	8.846976285
C	2.751010041	5.666309556	7.822477886
H	2.553242312	4.642057414	7.493823658
C	-1.687281003	6.092057501	10.90082247
H	-1.457721672	5.236357921	11.54311924
C	-2.274908201	8.292908562	9.297499249
H	-2.506071927	9.155143704	8.667953944
C	3.264216487	8.283915009	8.643612367
C	3.463622463	9.30736565	8.968489751
H	-0.311668387	1.669398676	8.021923796
H	-0.265639305	0.635427743	8.36678846
C	4.117233355	7.65778997	7.735726877
H	4.985558902	8.191813508	7.34310733
C	3.859367263	6.350219683	7.326376697
H	4.522721807	5.858090949	6.611534123
C	1.897518949	5.478001302	12.76492439
H	1.571855869	2.673278752	12.53097331
C	2.787290191	4.696632198	15.36137235
H	2.957019472	5.753878546	15.58294919

C	0.657947803	6.647101563	13.4072278
H	5.79354047	7.774779093	11.87348393
C	6.726449082	8.302519966	11.66178484
H	2.848132036	2.383327313	16.02480276
C	3.0658526	1.620308587	16.77589025
H	3.766712875	7.557522675	13.16881099
N	3.114471947	7.921211629	13.96747951
C	2.288876594	4.318888308	14.11313872
H	1.298846957	1.449699977	12.06625695
C	1.46250471	0.546536754	12.65987986
H	0.518498858	7.917588189	12.83298986
C	1.225465751	8.256943608	12.0717486
H	3.066976589	3.731387578	16.32271791
H	3.454847017	4.025098895	17.29985852
C	3.405643309	6.431611957	12.41727239
H	5.442088373	6.651946089	11.1258587
C	6.093470969	6.298630718	10.32376546
H	2.07078291	2.958342405	13.80664639
C	-0.255628275	6.228779285	14.38199554
H	-0.157594739	5.240543629	14.84013351
C	4.250444912	5.98372351	11.39475581
H	3.975342056	5.112577832	10.79234575
C	4.956008945	8.227058653	12.89319541
H	5.231968502	9.104999271	13.48160342
C	-0.511872499	8.761645589	13.2356942
H	-0.609672457	9.750419372	12.78210404
C	2.357273874	1.992026105	14.78502805
H	2.198025637	0.93148927	14.58467749
C	-1.416889302	8.341431949	14.20988463
C	-2.224902922	9.004948967	14.52654526
H	-1.287345097	7.07677032	14.78169485
C	-1.991410705	6.747383196	15.5491799
H	1.186317429	4.033085946	11.23286637
C	0.574669948	5.322526731	9.533327333
H	0.626404738	2.559499446	10.14046648
C	-0.469351356	4.300937879	7.081352593
H	-0.534404541	5.330772202	6.71933772
C	1.890749291	6.290813942	8.734514822
C	-3.106913914	7.964921897	10.36768097
H	-3.987988617	8.575238892	10.57945493
C	-0.806502589	1.940221641	6.751918796
C	-1.137248705	1.110864059	6.122308076

H	-1.148732959	7.519562159	9.022625355
H	-0.503236963	7.783094262	8.180354376
H	0.034953426	4.04337577	8.357497451
O	0.783644576	1.387800675	10.76541031
C	0.523365121	0.434312367	10.29845972
C	2.158640692	7.603305526	9.145368291
C	1.498693345	8.102161821	9.859578587
H	-0.888681449	3.250509232	6.271992156
H	-1.282828999	3.447632682	5.273360274
C	-0.845901868	6.4161833	9.829287155
H	-2.816531022	6.859867571	11.1664871
H	-3.463583652	6.601111403	12.00724849
H	0.117844674	2.721511706	8.846976285
H	2.751010041	5.666309556	7.822477886
H	2.553242312	4.642057414	7.493823658
H	-1.687281003	6.092057501	10.90082247

Table S14. XYZ coordinates of DFT optimized geometry of **$5^0{}_T$**.

Symbol	X	Y	Z
Co	1.181052428	4.032870175	11.23460589
P	0.573107818	5.3231221	9.533326041
N	0.628675635	2.56034236	10.14117712
C	-0.469204683	4.300412123	7.081537386
H	-0.535460752	5.330174365	6.719530663
C	1.890188997	6.291135441	8.736075728
C	-3.107871598	7.965680583	10.36911813
H	-3.988739818	8.576115575	10.58140506
C	-0.803106225	1.939345017	6.752176533
H	-1.132676109	1.109489716	6.122600084
C	-1.150352219	7.52034148	9.023049489
H	-0.505163627	7.783983697	8.180576782
C	0.035020887	4.043437963	8.357875029
C	0.787530085	1.387221382	10.76416029
H	0.529688947	0.434110011	10.29531176
C	2.157654826	7.603798886	9.146668409
H	1.497078067	8.102852724	9.860132302
C	-0.886953881	3.249484773	6.272076518
H	-1.281189585	3.446034098	5.273370129
C	-0.84735313	6.416733038	9.829323953
C	-2.817384317	6.860363928	11.16753137
H	-3.46410841	6.601446345	12.0084911
C	0.119712999	2.721818105	8.847603686

C	2.751148716	5.66624667	7.824982723
H	2.553623513	4.641842203	7.496667878
C	-1.688402453	6.092438126	10.90106296
H	-1.458961058	5.236347245	11.54298522
C	-2.276290638	8.293777381	9.298629715
H	-2.507644789	9.156192852	8.669403833
C	3.263601676	8.284230411	8.645527418
H	3.462737397	9.307822379	8.970117218
C	-0.308224407	1.669239246	8.022285444
H	-0.261072786	0.63531162	8.367066497
C	4.117352431	7.657742305	7.738570228
H	4.98600176	8.19163045	7.34648253
C	3.859874535	6.350014979	7.329506729
H	4.523827505	5.857649854	6.61538473
P	1.895449327	5.478821569	12.76507187
N	1.571589317	2.674287154	12.52879657
C	2.784837004	4.697039142	15.36108752
H	2.95350608	5.754358797	15.5831823
C	0.656912175	6.648791752	13.4076346
C	5.793577926	7.770510546	11.87080621
H	6.727167153	8.296909205	11.65876201
C	2.848073456	2.383436115	16.02282301
H	3.066477383	1.620107082	16.77339877
C	3.766028824	7.557316345	13.16564293
H	3.11402428	7.922683379	13.96373339
C	2.286866597	4.319436957	14.11259149
C	1.302393275	1.449242175	12.06425228
H	1.470218406	0.546615315	12.65735803
C	0.517853343	7.919280155	12.83327748
H	1.224726477	8.258241817	12.07178558
C	3.065566824	3.731514246	16.32178011
H	3.45318371	4.024910122	17.299114
C	3.404085198	6.430752705	12.4155298
C	5.441302546	6.646854426	11.12476671
H	6.092776249	6.291540424	10.3236319
C	2.070178587	2.959008196	13.80488276
C	-0.256494911	6.231007874	14.38280029
H	-0.158752126	5.242766049	14.84098444
C	4.248849277	5.980274942	11.39411936
H	3.973070563	5.1083199	10.79320619
C	4.956132979	8.225202451	12.88949499
H	5.232844977	9.10365357	13.47678644

C	-0.511920924	8.763885924	13.23631578
H	-0.609389761	9.752695495	12.78273545
C	2.357658183	1.992464348	14.7827979
H	2.199314409	0.931892688	14.58200869
C	-1.416763765	8.344214382	14.21092493
H	-2.224282137	9.00819896	14.52786731
C	-1.287629274	7.079563482	14.78282675
H	-1.991549364	6.750602704	15.55062556

Table S15. XYZ coordinates of DFT optimized geometry of **5**^{THF}$_{S}$.

Symbol	X	Y	Z
Co	7.225257694	8.38458121	4.509706963
P	6.776086431	9.601699017	2.704327157
P	8.904265086	7.143189175	3.742203899
O	5.702272767	7.008133851	4.346289757
N	7.942275231	7.980229453	6.197802641
C	3.768470451	12.3742105	3.406199678
H	3.085768376	13.02446532	2.85576792
C	8.419369201	11.8608281	2.894839661
H	7.786837502	12.1494148	3.738405139
C	4.650885077	11.54685112	2.717940179
H	4.65610865	11.54511287	1.625533539
C	8.808129301	6.889276017	6.427520747
C	9.367456553	6.291169467	5.283323785
C	7.851659014	3.675253474	1.792813558
H	7.549204791	2.661164963	2.064284658
C	5.506343352	10.69417503	4.830596386
C	10.42756156	8.037329629	3.279423285
C	8.952226915	10.34931209	1.087020169
H	8.734693397	9.45429016	0.498317556
C	4.618959983	11.53439084	5.516632258
H	4.577664371	11.53584303	6.606609917
C	5.061756219	7.036303096	0.019468375
H	4.748038924	5.989773044	0.020339595
C	8.284313334	5.353769811	0.115804606
H	8.308170686	5.666326355	-0.930473624
C	4.891774898	7.819805264	-1.119234816
H	4.431748921	7.393288498	-2.013898802
C	9.510879002	12.65403262	2.549215364
H	9.724305381	13.55869115	3.123252384
C	8.129497126	10.70364749	2.16263314
C	3.523375615	6.156665055	4.237261591

H	2.596745645	6.227099808	4.822392662
H	3.238741678	5.957021499	3.194921784
C	5.908689265	9.695328043	0.014055607
H	6.261287436	10.73046018	-0.006610661
C	10.66078444	9.268761516	3.905423969
H	9.911720617	9.682608738	4.586162482
C	10.57215585	4.714377006	6.655515608
H	11.26309396	3.874634778	6.752304433
C	5.651235377	7.579924353	1.159689966
H	5.806801271	6.956546942	2.043176827
C	3.762785061	12.36626382	4.802457428
H	3.071716558	13.0133979	5.347674733
C	7.414011821	8.70887841	7.193404168
H	7.668025635	8.54695855	8.242921947
C	10.00642188	5.295434748	7.792744171
H	10.25166387	4.901852062	8.781936347
N	6.393251246	9.784971641	5.443545356
C	10.04294213	11.14441983	0.743712676
H	10.67726653	10.86074614	-0.098894003
C	9.134654401	6.374452566	7.689675931
H	8.711388942	6.804158574	8.598398099
C	4.483218094	5.066362735	4.766502813
H	4.735508382	4.348847333	3.972933433
H	4.063699878	4.498445024	5.607540356
C	12.78722047	9.447355753	2.779606607
H	13.70864937	10.00001036	2.581401354
C	8.218841213	4.575864048	2.791594619
H	8.204207347	4.251100217	3.834921542
C	8.643749154	6.256218352	1.112548705
H	8.944087837	7.269451863	0.834053699
C	5.321032608	9.148271654	-1.12272696
H	5.200822262	9.761008753	-2.01902071
C	11.83933827	9.965861292	3.661232162
H	12.01154508	10.92516547	4.154034364
C	7.881280154	4.062861653	0.454312065
H	7.595773798	3.35492083	-0.327226126
C	4.338343519	7.447244341	4.336899685
H	4.138914534	7.996819143	5.273774374
C	6.067904441	8.916549562	1.170770571
C	10.255021	5.219900522	5.398687347
H	10.70248459	4.78262051	4.501595035
C	12.56040905	8.222778574	2.154149803

H	13.30382719	7.811983848	1.467120396
C	11.38830083	7.512014707	2.408551711
H	11.22962592	6.545466481	1.923720762
C	6.551313869	9.710187341	6.774721056
H	6.066465448	10.40080181	7.467769643
C	8.616459439	5.877773538	2.461833357
C	10.32390872	12.29742023	1.474288535
H	11.17901963	12.92141402	1.204181778
C	5.522496192	10.71648641	3.42326226
C	5.724315827	5.848888768	5.183555519
H	5.682517273	6.170836155	6.239617345
H	6.673772093	5.327148424	5.01809404
H	4.204862051	8.136156197	3.492787677

Table S16. XYZ coordinates of DFT optimized geometry of $\mathbf{5^{THF}}_{\mathbf{OS}}$.

Symbol	X	Y	Z
Co	7.324461914	8.437664358	4.483647098
P	6.807849377	9.66180656	2.691732861
P	8.974797958	7.129308813	3.756978738
O	5.674222919	7.007744424	4.376541341
N	7.890913906	7.8802117	6.244175359
C	3.838709915	12.46686565	3.405869944
H	3.200177156	13.15587664	2.849812265
C	8.42633762	11.93881117	2.8436373
H	7.785797406	12.23850277	3.677590103
C	4.741910062	11.65116366	2.730360722
H	4.809400342	11.70225597	1.641032542
C	8.785472689	6.827162541	6.458762762
C	9.419771715	6.294932894	5.315429318
C	7.907702512	3.628511833	1.878944842
H	7.633479376	2.611280814	2.167853678
C	5.466815904	10.68232793	4.845221041
C	10.48865857	8.014540594	3.257035569
C	8.974483238	10.40160645	1.060723301
H	8.765375839	9.494293645	0.487621746
C	4.555509822	11.51263382	5.517570466
H	4.457782733	11.46705673	6.603353798
C	5.050147305	7.056302083	0.080384527
H	4.73034544	6.011884097	0.106225502
C	8.246418257	5.302549202	0.174818815
H	8.226513078	5.606883969	-0.873982756
C	4.867575477	7.818803104	-1.070641305

H	4.392141667	7.377739457	-1.950067703
C	9.514988688	12.73318434	2.489234168
H	9.720853873	13.64848398	3.048997399
C	8.146001777	10.76879278	2.128284715
C	3.488876588	6.180470623	4.205030614
H	2.552965557	6.237091836	4.777098153
H	3.219262888	6.02620124	3.150866944
C	5.909156128	9.711190307	0.011319551
H	6.26524849	10.74435078	-0.034036448
C	10.72124134	9.254728237	3.866647648
H	9.992531888	9.665289509	4.572714915
C	10.65620349	4.73722056	6.68733637
H	11.38779113	3.932932188	6.784775791
C	5.658414156	7.618461945	1.20150142
H	5.820319244	7.014415969	2.097532396
C	3.75519504	12.39291106	4.79815308
H	3.047812857	13.02952395	5.334952186
C	7.276570419	8.568096979	7.211543053
H	7.455415421	8.375232522	8.272929088
C	10.01240654	5.245405936	7.818729954
H	10.23772129	4.829577534	8.803652813
N	6.300571137	9.748814909	5.467885403
C	10.05970171	11.19905219	0.707349766
H	10.69753668	10.90771132	-0.129976331
C	9.089202216	6.279312081	7.715712258
H	8.608261507	6.65657992	8.619421799
C	4.429351077	5.060588679	4.705345343
H	4.677170189	4.364097755	3.891814142
H	3.994890454	4.471832043	5.524416134
C	12.8045332	9.463441832	2.671389925
H	13.70851282	10.03118886	2.438589781
C	8.294803619	4.542839004	2.857615667
H	8.32575219	4.228297119	3.903759815
C	8.623832013	6.219525267	1.151113037
H	8.892370406	7.237315962	0.856134795
C	5.303138054	9.14479249	-1.106192573
H	5.172476782	9.740594432	-2.012369883
C	11.87736307	9.972382228	3.580627414
H	12.047716	10.93832549	4.060817514
C	7.880821889	4.006909092	0.537815086
H	7.579883137	3.287853152	-0.227612528
C	4.318630024	7.456026165	4.368973261

H	4.106298433	7.967867314	5.32467603
C	6.080450834	8.953097081	1.179634585
C	10.3605172	5.270370253	5.4365056
H	10.86369983	4.885995441	4.544521725
C	12.57848511	8.231423444	2.060537687
H	13.30621322	7.830336415	1.351378283
C	11.42699916	7.502086032	2.355677707
H	11.26445975	6.532521448	1.877825775
C	6.40978268	9.582462218	6.789403442
H	5.875438534	10.21193923	7.506468492
C	8.65260903	5.849184737	2.502473442
C	10.3318841	12.36507113	1.421610704
H	11.18298396	12.99085687	1.143037413
C	5.555562734	10.76554351	3.438018371
C	5.678997457	5.81465177	5.156529524
H	5.636823245	6.082950266	6.227862956
H	6.622074278	5.287157319	4.970740827
H	4.198128193	8.182477911	3.554196936

Table S17. XYZ coordinates of DFT optimized geometry of $\mathbf{5}^{\mathrm{THF}}{}_{\mathrm{T}}$.

Symbol	X	Y	Z
Co	7.355949991	8.463422798	4.482427839
P	6.814258673	9.679426501	2.687317756
P	8.995584612	7.132914757	3.759216886
O	5.663584132	7.007118521	4.394279572
N	7.860542262	7.847290262	6.245421626
C	3.860119684	12.49499391	3.409256146
H	3.237380322	13.2001406	2.855607477
C	8.431222921	11.95645992	2.8295132
H	7.790087615	12.2589105	3.662149187
C	4.769545289	11.68584531	2.734880145
H	4.858871872	11.75975279	1.648369761
C	8.764583445	6.806605708	6.458446828
C	9.428803839	6.299099089	5.319492183
C	7.91691005	3.631705728	1.891847243
H	7.645769558	2.614477602	2.183570345
C	5.450361536	10.67009087	4.843932524
C	10.50850696	8.014087811	3.251714114
C	8.978525966	10.41448666	1.049970596
H	8.768946409	9.505886846	0.479025574
C	4.529471142	11.492791	5.514719183
H	4.409319256	11.423743	6.597057482

C	5.047521048	7.048638033	0.109603884
H	4.725167164	6.005379178	0.147802836
C	8.243863	5.303958403	0.183264241
H	8.217845147	5.606615174	-0.865872477
C	4.863554566	7.798992856	-1.049180319
H	4.3845411	7.3495148	-1.922368328
C	9.520897978	12.74901941	2.473648548
H	9.727425051	13.6655001	3.031219439
C	8.149888081	10.78506583	2.116490243
C	3.479285018	6.179588835	4.203774406
H	2.541083505	6.229724479	4.772765111
H	3.213463203	6.035918861	3.147102504
C	5.911308692	9.701238745	0.009263549
H	6.267790597	10.73372353	-0.047678238
C	10.74245199	9.255000227	3.859499596
H	10.01962553	9.664022303	4.573210148
C	10.66466121	4.74084052	6.69217258
H	11.40746409	3.947272147	6.792242755
C	5.660015972	7.621504867	1.222951689
H	5.821618083	7.028078546	2.126215733
C	3.748931785	12.39220625	4.798247676
H	3.034886678	13.02205004	5.334243323
C	7.258225084	8.551602483	7.209720295
H	7.449019774	8.372932478	8.271677376
C	9.987781335	5.21959424	7.817735135
H	10.19766956	4.789780141	8.800098029
N	6.265281142	9.724233418	5.465529787
C	10.06442347	11.21026239	0.695181604
H	10.70204157	10.91684732	-0.14158778
C	9.05042234	6.23983776	7.712497303
H	8.540707672	6.590655909	8.611100431
C	4.418739373	5.055339184	4.695904511
H	4.671642534	4.368782933	3.875408147
H	3.981559457	4.456230089	5.505985711
C	12.81466667	9.470918555	2.647548939
H	13.71479536	10.04167394	2.407317614
C	8.30764381	4.547957563	2.867248144
H	8.344474916	4.236019409	3.914018795
C	8.624621037	6.223101972	1.156138814
H	8.889695302	7.241063526	0.858548461
C	5.301452258	9.123716547	-1.100400109
H	5.168940859	9.709972124	-2.012502905

C	11.89324339	9.97727828	3.564164387
H	12.0642047	10.94388156	4.04274154
C	7.882630979	4.00817863	0.550339393
H	7.579142565	3.287388242	-0.212433708
C	4.308885992	7.453790398	4.383706295
H	4.089522958	7.957267832	5.342257801
C	6.08446127	8.954984947	1.18479048
C	10.38428361	5.28818638	5.444375025
H	10.91069479	4.92568576	4.556582398
C	12.58774134	8.238102002	2.038654919
H	13.3112483	7.839439354	1.323834542
C	11.44073151	7.505030105	2.342347141
H	11.276445	6.535663357	1.864653147
C	6.392794579	9.566932584	6.787167391
H	5.875585277	10.20918104	7.505772031
C	8.661202055	5.85404007	2.507631646
C	10.3375355	12.37771093	1.407043772
H	11.18917311	13.00213935	1.127077406
C	5.562986556	10.77882693	3.439213089
C	5.665089118	5.80709847	5.16131271
H	5.615877319	6.062926559	6.235467884
H	6.609498585	5.281258014	4.976393838
H	4.19125786	8.187139427	3.574529015

Table S18. XYZ coordinates of DFT optimized geometry of $\mathbf{5}^{2THF}{}_{S}$.

Symbol	X	Y	Z
Co	7.065585848	8.963086798	4.151398303
P	7.225839888	8.9367303	1.817341522
P	8.738019939	7.489326148	4.855137533
O	5.674049838	7.495881118	4.158228069
N	6.725800397	9.185128054	6.006022535
C	4.24674579	11.14115233	0.007475906
H	3.878874117	11.36525369	-0.994851097
C	8.64529744	10.99133288	0.465562812
H	7.722509988	11.57589667	0.500459033
C	5.332470226	10.29960872	0.189775018
H	5.82295309	9.855386861	-0.680562928
C	7.466632674	8.612777068	6.994095911
C	8.531484188	7.739737175	6.635702742
C	7.97184518	3.441409331	5.201970386
H	7.612274741	2.75022811	5.967615522
C	5.174580085	10.54323345	2.631877033
C	10.51370702	7.73427547	4.463572637

C	9.863091056	8.955304542	0.907847365
H	9.907402788	7.938602064	1.307336413
C	4.07264419	11.41125992	2.420678951
H	3.557395968	11.85679883	3.272053637
C	6.365951052	5.079610188	0.643865066
H	6.034460635	4.156119932	1.125157243
C	8.815578563	3.851295597	2.978916641
H	9.112147135	3.486712836	1.992926087
C	6.581806547	5.118928675	-0.730722577
H	6.406201853	4.229372137	-1.340143726
C	9.776649261	11.53064753	-0.146205468
H	9.732708322	12.53615456	-0.570782105
C	8.682626017	9.702797861	1.010767182
C	3.785217267	6.202764345	3.606774181
H	2.701689612	6.30651085	3.752761884
H	3.936630638	5.569916182	2.721667529
C	7.261330575	7.430888719	-0.554954954
H	7.641794839	8.336826296	-1.033871245
C	10.97053004	8.90724905	3.855345103
H	10.2546998	9.664079777	3.536879673
C	9.140146968	7.412548438	8.959974717
H	9.785456841	6.966326067	9.718059101
C	6.586097816	6.216791923	1.419664078
H	6.439500742	6.171155625	2.498214286
C	3.631185548	11.69462577	1.139292794
H	2.775635648	12.36355694	1.01400683
C	5.678357738	10.08359555	6.192057613
H	5.338397853	10.34537155	7.195023106
C	8.079642805	8.254282249	9.324583675
H	7.895146932	8.46150511	10.38203668
N	5.641758212	10.18730836	3.859951503
C	10.98989686	9.492173554	0.293249
H	11.90343929	8.897053984	0.226513764
C	7.258214216	8.842884458	8.378186668
H	6.452875468	9.500304075	8.706821389
C	4.494566829	5.618557364	4.845773913
H	5.158193522	4.785789209	4.567991084
H	3.796477826	5.249946774	5.609028928
C	13.25156724	8.128344802	4.004210941
H	14.31891954	8.282709845	3.829400978
C	8.083911779	4.800059171	5.497742988
H	7.826831755	5.155682053	6.49878135

C	8.919907429	5.207419814	3.270365104
H	9.304005644	5.890019801	2.507604879
C	7.036276105	6.29586899	-1.327511431
H	7.221024505	6.329231286	-2.403534603
C	12.33220957	9.103331809	3.627769457
H	12.67025449	10.02282863	3.143267338
C	8.334574259	2.96563273	3.943398162
H	8.254068359	1.89981911	3.717437279
C	4.430402347	7.577115103	3.419695737
H	3.826574199	8.384104071	3.859159745
C	7.021876883	7.406705632	0.828704242
C	9.355965727	7.165596701	7.613692511
H	10.1826037	6.51731596	7.311056558
C	12.80436939	6.945079482	4.594018642
H	13.51878594	6.169157386	4.877926771
C	11.44600402	6.74532574	4.817648306
H	11.1104877	5.802152086	5.256409912
C	5.115040791	10.60006833	5.080907469
H	4.290296874	11.3128806	5.121523031
C	8.546152427	5.697392265	4.528540428
C	10.95085038	10.78487285	-0.229467158
H	11.83451568	11.20779882	-0.713118566
C	5.80781044	10.00789745	1.475933242
C	5.319380118	6.783776477	5.364083543
H	4.737476825	7.46080824	6.009019276
H	6.233749868	6.496555302	5.887299807
H	4.656966799	7.830765682	2.378809977
O	8.307264506	10.55437842	4.28127576
C	9.024186169	10.90580242	5.490418977
C	7.985632364	11.82944866	3.684321565
C	9.93039377	12.06609561	5.074108756
H	8.285701145	11.20489629	6.248073512
H	9.561216525	10.02246928	5.851085961
C	9.300735279	12.58421249	3.764813182
H	7.61867347	11.66210806	2.669838157
H	7.186354921	12.30095165	4.277902438
H	10.96347542	11.72689955	4.917131492
H	9.954966252	12.83267468	5.860063321
H	9.928007174	12.33198603	2.896274164
H	9.143904578	13.67110095	3.759817973

Table S19. XYZ coordinates of DFT optimized geometry of $\mathbf{5}^{2THF}{}_{OS}$.

Symbol	X	Y	Z
Co	7.168819874	8.870326395	4.050621551
P	7.297206816	8.889596484	1.789904767
P	8.770665826	7.444985212	4.776689013
O	5.36711895	7.284725625	4.14783058
N	6.794353809	9.141297836	5.943036531
C	4.209981931	10.95572346	-0.040381452
H	3.820431373	11.13440767	-1.044325808
C	8.604694625	11.04008477	0.466914358
H	7.647481328	11.56710407	0.479780533
C	5.328938434	10.15017139	0.144544606
H	5.81810135	9.693689604	-0.720079446
C	7.557869114	8.584100935	6.962820517
C	8.613658791	7.728373757	6.574742677
C	7.866528376	3.442614383	5.291776014
H	7.504121117	2.790862414	6.090044752
C	5.185697487	10.46993758	2.557688927
C	10.54862818	7.673186221	4.41133249
C	9.93709915	9.083815359	0.938510586
H	10.03454045	8.070167736	1.337405035
C	4.061395792	11.29233575	2.35702959
H	3.543916708	11.74098102	3.206234266
C	6.293711006	5.104092128	0.594881434
H	5.855897505	4.22005824	1.065146917
C	8.625520661	3.753446959	3.023046435
H	8.85282181	3.350753415	2.033403505
C	6.627042869	5.08831952	-0.757475044
H	6.439111286	4.195184963	-1.358181584
C	9.721168693	11.66127795	-0.092518657
H	9.628722201	12.66887101	-0.504358464
C	8.705128929	9.748355418	0.995774462
C	3.453979531	6.027057149	3.62089093
H	2.370512197	6.146999894	3.758303275
H	3.601757191	5.383453635	2.742331603
C	7.449879908	7.353545219	-0.582924099
H	7.919969419	8.223192909	-1.050214559
C	10.99248051	8.915183946	3.944393045
H	10.26368836	9.698622989	3.72584646
C	9.281734158	7.454805467	8.885184867
H	9.955858693	7.030427524	9.631540671
C	6.533150587	6.243754994	1.360654856

H	6.295105376	6.248798423	2.426519928
C	3.588254443	11.52885846	1.072733996
H	2.710247284	12.16481961	0.936555243
C	5.79478761	10.00726369	6.124859792
H	5.452795249	10.30818188	7.119442161
C	8.223116727	8.280505954	9.27459678
H	8.065410157	8.498061525	10.33373471
N	5.699809142	10.12591199	3.803022116
C	11.04873251	9.701867236	0.374238564
H	12.00235899	9.170095046	0.340190664
C	7.369867154	8.841906172	8.333465589
H	6.563299965	9.495466527	8.668738174
C	4.150233239	5.439929737	4.868833869
H	4.774317514	4.574096004	4.600918302
H	3.443788529	5.115114885	5.644749232
C	13.2771015	8.133029472	3.988387742
H	14.34285594	8.312328299	3.827671018
C	8.062038993	4.800367054	5.545352355
H	7.859917169	5.195474444	6.544000719
C	8.812178017	5.109453476	3.272416291
H	9.18656593	5.753332606	2.471534967
C	7.211757394	6.211739859	-1.343380115
H	7.485921855	6.198416789	-2.400731682
C	12.35199303	9.144178997	3.738607586
H	12.68594742	10.11672987	3.368415671
C	8.146070726	2.917524057	4.031666739
H	7.998640921	1.852552917	3.837628587
C	4.143194004	7.385345352	3.426493277
H	3.542843534	8.211652243	3.848795027
C	7.099859475	7.38142037	0.775081349
C	9.472059646	7.185249447	7.533928157
H	10.3020973	6.54674602	7.21987715
C	12.83985555	6.885042221	4.434022431
H	13.56088565	6.085501247	4.619058313
C	11.48312547	6.652939061	4.642828617
H	11.15169022	5.666808009	4.979040496
C	5.197303491	10.5275993	4.972810168
H	4.358805534	11.22675632	5.041881892
C	8.52843306	5.647720917	4.534511753
C	10.94426897	10.99525016	-0.137669795
H	11.81639173	11.48031422	-0.582349801
C	5.828816816	9.915687091	1.427733491

C	5.037895637	6.588234604	5.341290458
H	4.497648174	7.268844783	6.026144323
H	5.967111599	6.273250663	5.828071576
H	4.371948554	7.628963302	2.380481917
O	8.492876432	10.86380135	4.405541791
C	9.200094775	11.20251281	5.59343234
C	8.199460632	12.1038538	3.779672585
C	10.12710066	12.36042979	5.191158789
H	8.475652809	11.51219829	6.36861555
H	9.72535214	10.30871606	5.955550766
C	9.507838423	12.88319533	3.87565787
H	7.863535785	11.90524809	2.756320932
H	7.378888623	12.60750254	4.325310258
H	11.15572185	12.00542724	5.036054873
H	10.16459052	13.13012658	5.974228577
H	10.1467637	12.63699294	3.013803419
H	9.348981541	13.97017524	3.874054438

Table S20. XYZ coordinates of DFT optimized geometry of **$5^{2THF}{}_{T}$**.

Symbol	X	Y	Z
Co	7.168329528	8.87231709	4.052146239
P	7.298799118	8.889591894	1.790661057
P	8.77215575	7.446867282	4.775753503
O	5.368697481	7.281046305	4.148455673
N	6.793328144	9.140617624	5.941065533
C	4.212582213	10.95675828	-0.039482654
H	3.823929539	11.13677315	-1.043533147
C	8.607605037	11.03790662	0.465396861
H	7.650705281	11.56552941	0.477510583
C	5.331783827	10.15170042	0.145411337
H	5.822089265	9.696573105	-0.719285214
C	7.556882327	8.583588623	6.961146254
C	8.614065873	7.729477218	6.573521186
C	7.869540587	3.443981478	5.288008093
H	7.507840657	2.791497458	6.08600093
C	5.18636008	10.46792006	2.55898354
C	10.55018111	7.675290701	4.410865453
C	9.938761115	9.081360759	0.93905647
H	10.03563692	8.068049038	1.338924673
C	4.061514085	11.28962996	2.358342382
H	3.542657189	11.7365063	3.207608794
C	6.290768343	5.105027885	0.595389964

H	5.851147013	4.221631279	1.065158768
C	8.62751378	3.756651589	3.019184558
H	8.854605471	3.354725645	2.02918704
C	6.625250055	5.08876773	-0.756666238
H	6.436444317	4.195958565	-1.357582771
C	9.724426988	11.65768481	-0.094902215
H	9.632571737	12.6648285	-0.507971621
C	8.70724375	9.746798661	0.995845839
C	3.449536689	6.031191568	3.62624805
H	2.366453031	6.1547722	3.763445019
H	3.59533807	5.386205804	2.748363025
C	7.451353981	7.352696384	-0.581239298
H	7.923115002	8.221673009	-1.048076989
C	10.99434253	8.917179496	3.943979078
H	10.26562387	9.700488064	3.724818793
C	9.281452086	7.455954727	8.884143218
H	9.95576697	7.03229073	9.630729326
C	6.531221488	6.244228811	1.361536762
H	6.292070881	6.249458074	2.42716791
C	3.589424521	11.52773895	1.073998638
H	2.711025668	12.16320886	0.938039065
C	5.792570635	10.005916	6.125083043
H	5.451337108	10.30514512	7.120296983
C	8.221325539	8.279943702	9.273198082
H	8.062661591	8.496914272	10.33231596
N	5.699437311	10.12242005	3.80452267
C	11.05076698	9.697951831	0.373936262
H	12.00400138	9.165457269	0.340372615
C	7.36778057	8.840457695	8.331857019
H	6.560124401	9.49264109	8.667102617
C	4.144070628	5.443070147	4.87467809
H	4.762319674	4.572685104	4.60812812
H	3.436749719	5.124624142	5.652441801
C	13.27892535	8.135079454	3.989184293
H	14.34475484	8.314359753	3.828961709
C	8.064564192	4.801639024	5.542437159
H	7.862861044	5.195955431	6.541490253
C	8.813688451	5.112567057	3.269406899
H	9.187491749	5.757095671	2.468794607
C	7.212171382	6.21132189	-1.342020449
H	7.487200573	6.197749657	-2.399142831
C	12.3539432	9.146155273	3.73874983

H	12.68812341	10.11860232	3.368483272
C	8.148784742	2.919866302	4.027417009
H	8.001691982	1.854976796	3.832696992
C	4.14323957	7.386811607	3.430358017
H	3.547321635	8.215182998	3.854771174
C	7.100192968	7.381012769	0.776485628
C	9.472851078	7.187188889	7.532963202
H	10.30398229	6.550073951	7.218997423
C	12.84141518	6.887131054	4.43481876
H	13.56231783	6.087597353	4.620364553
C	11.4846075	6.655069211	4.642919991
H	11.15307356	5.668915078	4.978993912
C	5.195622247	10.52604434	4.973929168
H	4.357845769	11.22602011	5.04139403
C	8.530106981	5.64983239	4.531922873
C	10.94711137	10.99083889	-0.139406244
H	11.81952396	11.4748335	-0.584679122
C	5.830732482	9.915801629	1.428804286
C	5.039221614	6.587177254	5.343458904
H	4.505068941	7.271237183	6.029554554
H	5.968297697	6.267358518	5.827300104
H	4.370207587	7.629755981	2.38379747
O	8.490782954	10.86152895	4.401850936
C	9.195987247	11.19988892	5.591287889
C	8.197616662	12.10210402	3.776654278
C	10.1228964	12.35859565	5.19132646
H	8.470081841	11.5082557	6.365544286
H	9.721286479	10.30623276	5.953647705
C	9.505793287	12.88135647	3.874830322
H	7.86340755	11.90434595	2.752576011
H	7.376130641	12.60482878	4.321655143
H	11.15215706	12.00465874	5.038086972
H	10.15808414	13.12784313	5.974941883
H	10.14613832	12.63479612	3.014123579
H	9.346999414	13.96834433	3.872428496

Table S21. XYZ coordinates of DFT optimized geometry of **6** (S = 1/2).

Symbol	X	Y	Z
Co	1.469854158	4.151100896	10.29921245
P	-0.090940853	5.549091698	9.561525429
N	0.531346471	2.739833083	9.329736483

C	0.47510609	6.882534913	8.475463614
C	-3.068817014	7.097718278	12.69968773
H	-3.787090869	7.444001712	13.44633486
C	-2.065330186	7.314045554	10.51285323
H	-1.99950411	7.834559677	9.553467422
C	-1.041202983	4.366506467	8.535771889
C	1.089622741	1.587437758	9.474218859
H	0.71290186	0.667488071	9.012984871
C	0.750690641	8.14920816	9.009333893
H	0.523078863	8.370749813	10.05485326
C	-1.238819085	6.216415665	10.78953114
C	-2.253036023	6.000567264	12.97587017
H	-2.326138726	5.48750416	13.9369585
C	-0.621644631	3.021817251	8.555555385
C	0.76968438	6.615457767	7.131664267
H	0.558480916	5.633219875	6.699086041
C	-1.337212843	5.562406235	12.02457451
H	-0.691961051	4.709237742	12.25811673
C	-2.974713445	7.752978852	11.4715624
H	-3.617297313	8.609227603	11.25589391
C	1.300549826	9.141127191	8.203084994
H	1.503630499	10.12888137	8.622148098
C	1.58574867	8.87394794	6.864192287
H	2.01077324	9.655724609	6.230575508
C	1.32107742	7.613148034	6.330038106
H	1.536409626	7.406187066	5.279486173
P	2.757717711	5.48172322	11.5200786
N	2.671087526	2.692965227	10.79582724
C	5.052342549	4.538487774	12.91875394
H	5.196021728	5.559512635	13.28143477
C	1.97332504	6.19945821	12.98468168
C	5.19043292	8.606345098	9.193276524
H	5.779923941	9.332777297	8.629224388
C	5.782958013	2.235096354	12.80696167
H	6.498320304	1.457747062	13.08315493
C	4.279944953	7.840496884	11.29662343
H	4.157329537	7.973010599	12.3750717
C	3.967949398	4.234262449	12.09483865
C	2.295872315	1.559743616	10.31035911
H	2.823383066	0.616304108	10.4910966
C	1.412718133	7.482332522	12.91827612
H	1.547524267	8.099339162	12.02656123

C	5.955521295	3.540716711	13.27628579
H	6.804380643	3.780635956	13.92004684
C	3.685299305	6.756352477	10.63699509
C	4.605603469	7.524642312	8.534859552
H	4.73219842	7.401348376	7.457364982
C	3.796467768	2.916437236	11.62788869
C	1.797882013	5.421595009	14.13692176
H	2.236235756	4.421737082	14.20616829
C	3.852441873	6.602901239	9.254494275
H	3.385522597	5.765269162	8.726302377
C	5.027884258	8.763691921	10.57005341
H	5.490597651	9.609676332	11.08284858
C	0.692986753	7.984323875	13.99805792
H	0.264492464	8.987248723	13.94396976
C	4.709366878	1.916258808	11.98511631
H	4.602168855	0.891135594	11.62564954
C	0.521971695	7.208649716	15.14450525
H	-0.039041492	7.606454499	15.99325573
C	1.075409211	5.930358751	15.21409361
H	0.953069483	5.327702648	16.11669254
C	-1.326131185	2.05102083	7.83276212
C	-2.165163509	4.726385756	7.790812873
C	-2.860757029	3.759215176	7.069828224
C	-2.440616473	2.426105832	7.093521819
H	-2.501417196	5.766285165	7.773083496
H	-3.738828695	4.042607819	6.485632502
H	-2.990344395	1.670551703	6.528478533
H	-1.0196231	1.003447154	7.836372329

Table S22. XYZ coordinates of DFT optimized geometry of **6** (S = 3/2).

Symbol	X	Y	Z
Co	1.395115313	4.316062351	10.48698065
P	-0.471859875	5.535522993	9.766476288
N	0.592035109	2.789263299	9.289760689
C	0.200119885	6.810331627	8.656614879
C	-3.64098816	7.315412384	12.56490164
H	-4.397723756	7.723645521	13.23878909
C	-2.609836852	7.250577906	10.38132638
H	-2.547380107	7.624293533	9.35516757
C	-1.228009137	4.267353969	8.68133863
C	1.139710469	1.648604921	9.494938938
H	0.756701445	0.707949306	9.078569479
C	0.465822668	8.088695018	9.169893087

H	0.157498537	8.349976841	10.18603772
C	-1.716540842	6.271653756	10.84211222
C	-2.747189954	6.351142952	13.02828029
H	-2.798465272	6.004067179	14.06243445
C	-0.584730637	3.016888566	8.531447376
C	0.587033152	6.491481212	7.348379242
H	0.376035028	5.50364512	6.929169981
C	-1.781560653	5.831754058	12.17030086
H	-1.068255933	5.091050301	12.54505706
C	-3.569542764	7.768098304	11.24602797
H	-4.266447826	8.529772017	10.89003668
C	1.102133531	9.038347625	8.376104389
H	1.295653238	10.03638017	8.775286081
C	1.482011897	8.719459294	7.071722893
H	1.9728205	9.470273456	6.448284806
C	1.224261488	7.448902693	6.559529334
H	1.507203805	7.202590725	5.533698437
P	3.187396026	5.535386802	11.35117736
N	2.650574249	2.684093044	10.98670007
C	5.418096248	4.418728792	12.71195521
H	5.769601722	5.439790632	12.87655909
C	2.278361924	6.196378345	12.7832704
C	5.595888054	8.875792239	9.337924883
H	6.166186414	9.668792177	8.848615406
C	5.707609844	2.039345968	13.02367333
H	6.269447555	1.201956002	13.44264667
C	4.875727956	7.770399499	11.36038859
H	4.864200752	7.714041722	12.45280075
C	4.244138908	4.191240534	11.99337839
C	2.355748128	1.620760689	10.33573535
H	2.974801265	0.713657553	10.34798901
C	1.723115657	7.482847313	12.70181474
H	1.962546721	8.130909817	11.8540921
C	6.149629385	3.347792642	13.22274943
H	7.064510828	3.535408281	13.78848321
C	4.143038493	6.855214221	10.58820478
C	4.859953883	7.975165121	8.56979799
H	4.849795978	8.060654959	7.481198488
C	3.821915013	2.862519842	11.76514964
C	1.978793578	5.376929314	13.88144383
H	2.420338296	4.379826574	13.96982929
C	4.130846095	6.964660927	9.191860815

H	3.53867667	6.272250908	8.586176713
C	5.600748391	8.776912574	10.73099114
H	6.171984894	9.489677861	11.32959608
C	0.88519728	7.943679881	13.71236633
H	0.463188215	8.949206556	13.651202
C	4.54859475	1.791635401	12.29563401
H	4.205977367	0.762667324	12.16633406
C	0.589571507	7.126265975	14.8044035
H	-0.064732567	7.492953693	15.59848214
C	1.137728617	5.847353678	14.88944831
H	0.921014236	5.213592771	15.75223458
C	-1.108023177	2.061484051	7.651824815
C	-2.406295485	4.514008641	7.974311748
C	-2.930259331	3.552860548	7.112955117
C	-2.273694451	2.333041537	6.945426803
H	-2.923582231	5.469007368	8.093126857
H	-3.849635756	3.759644589	6.561346366
H	-2.671477671	1.586538722	6.254819527
H	-0.603193547	1.106210592	7.495278983

Table S23. XYZ coordinates of DFT optimized geometry of $\mathbf{7_s}$

Symbol	X	Y	Z
Co	5.82118213	4.155845685	12.11250847
P	4.802117738	2.278849699	11.54922486
P	6.814476686	4.72584443	10.19721419
P	4.19330696	5.681415256	12.14923331
N	7.284718796	4.955808053	12.94112493
N	5.462378205	3.528459095	13.84710933
C	4.36714172	2.693711946	14.14358625
C	2.133997195	1.027762722	14.47701699
H	1.264653784	0.381974427	14.61494276
C	3.763661965	2.098539069	10.06695851
C	2.694855055	1.186401615	13.21151331
H	2.275833208	0.65173959	12.3546297
C	8.26203662	5.717399562	12.26047507
C	8.188717604	5.717344682	10.85656844
C	10.17815028	7.07568363	10.71227333
H	10.9330834	7.593668729	10.11762896
C	3.798088985	2.021768209	13.04499172
C	2.420963013	2.496128032	10.07671806
H	1.950763126	2.820363746	11.00936846
C	4.347922155	1.692351125	8.857840305
H	5.399690551	1.393282083	8.825202994

C	5.934928462	5.766066384	8.979474994
C	6.266688921	4.058820287	14.79152299
H	6.139597822	3.843921556	15.85442075
C	3.811633069	2.511453927	15.41547708
H	4.225759889	3.022422797	16.28615006
C	9.149394538	6.377012267	10.08818754
H	9.095158758	6.335119521	8.996525209
C	7.276125704	4.853258262	14.29184015
H	8.015653551	5.343538171	14.92632109
C	9.300120621	6.426370519	12.88046795
H	9.39101912	6.45440535	13.96665498
C	7.635332761	3.417248857	9.212853485
C	7.574024296	3.32186847	7.818208319
H	7.038858609	4.070166399	7.230810481
C	4.94798986	5.168242615	8.181157592
H	4.758575769	4.095195484	8.252151095
C	10.24058602	7.099675829	12.10689263
H	11.04628802	7.64448911	12.60466862
C	1.673653586	2.482324805	8.899821635
H	0.625474647	2.789350914	8.919896796
C	8.367037211	2.46374879	9.9317093
H	8.446335874	2.549622165	11.01746867
C	5.877823542	0.791044668	11.60223038
C	2.700175134	1.6872562	15.56970963
H	2.26447453	1.561237351	16.56366669
C	7.022940698	0.832344902	12.40835869
H	7.288805288	1.758909966	12.92467333
C	5.353329478	7.916654661	8.022497479
H	5.51402485	8.995624988	7.965849351
C	4.382183091	7.31185974	7.226987714
H	3.77674803	7.916518764	6.547847652
C	8.993289521	1.408993915	9.276462192
H	9.547230268	0.665623574	9.854245242
C	4.184678213	5.932865837	7.305127232
H	3.421615812	5.44707599	6.692588506
C	6.130502424	7.149336458	8.891576296
H	6.888894488	7.641768099	9.504490884
C	5.532096599	-0.407438828	10.96787575
H	4.625945052	-0.473407528	10.36108031
C	7.82091285	-0.298486081	12.55632073
H	8.710910113	-0.254210435	13.18844424
C	4.952899364	7.296519488	12.54655176
H	5.503171421	7.211264174	13.49483669
H	4.192602878	8.086060153	12.63909426

H	5.668128664	7.574618895	11.75974141
C	2.259101843	2.07083265	7.70316343
H	1.670043767	2.055011676	6.783203772
C	3.088371843	6.078218845	10.7464453
H	3.62659741	6.648560608	9.980421543
H	2.244857192	6.685675309	11.10567402
H	2.701201306	5.161009376	10.283091
C	8.203638483	2.263582861	7.162687873
H	8.148687914	2.195819335	6.073705335
C	3.596143571	1.673912702	7.685145952
H	4.062102521	1.349083952	6.751928275
C	8.902464601	1.301224615	7.888692535
H	9.387880035	0.470300368	7.371538218
C	7.487049414	-1.48122846	11.89759874
H	8.116968605	-2.367060058	12.007582
C	6.339966768	-1.534234812	11.10805977
H	6.063692343	-2.462125791	10.60218613
C	2.987339611	5.477483834	13.50922956
H	2.3883375	4.567956272	13.35994467
H	2.316336369	6.347888175	13.54829367
H	3.512460313	5.387089724	14.46944044

Table S24. XYZ coordinates of DFT optimized geometry of $\mathbf{7_T}$

Symbol	X	Y	Z
Co	5.877646	4.015058	12.0918
P	4.716535	2.196254	11.479574
P	6.985353	4.660007	10.25588
P	4.219739	5.815421	12.109692
N	7.215928	5.05888	13.050957
N	5.380309	3.447854	13.883569
C	4.305268	2.596793	14.131569
C	2.083595	0.897953	14.408159
H	1.21639	0.244531	14.520497
C	3.52864	2.129622	10.100987
C	2.66728	1.078538	13.156874
H	2.255263	0.564797	12.283766
C	8.163524	5.853599	12.41317
C	8.208879	5.781176	11.003608
C	10.092781	7.299836	10.957988
H	10.852428	7.850917	10.400474
C	3.768034	1.923779	13.013128
C	2.285762	2.764407	10.222512
H	1.984474	3.197778	11.180419
C	3.889212	1.5604	8.872478

H	4.853992	1.057651	8.76341
C	6.064014	5.591013	8.979681
C	6.181156	3.962587	14.820894
H	6.088532	3.694156	15.87736
C	3.713975	2.403075	15.390319
H	4.093555	2.928888	16.268246
C	9.188178	6.481525	10.290731
H	9.247906	6.375492	9.203727
C	7.178151	4.832541	14.369896
H	7.906659	5.268563	15.059317
C	9.074775	6.695879	13.076414
H	9.044887	6.798531	14.162312
C	7.999381	3.40113	9.391579
C	7.510716	2.673452	8.300664
H	6.54928	2.926395	7.850174
C	5.013445	4.945149	8.311767
H	4.759104	3.910918	8.549141
C	10.019482	7.408869	12.350612
H	10.719077	8.058613	12.881979
C	1.422005	2.83615	9.13198
H	0.452794	3.328755	9.238755
C	9.248125	3.061522	9.929967
H	9.651833	3.618717	10.779424
C	5.753309	0.704392	11.30997
C	2.615679	1.560409	15.51748
H	2.15748	1.426782	16.500304
C	7.127619	0.821122	11.534683
H	7.563858	1.797073	11.763125
C	5.52728	7.624395	7.772511
H	5.725772	8.68013	7.574294
C	4.505655	6.96455	7.092016
H	3.901207	7.501454	6.357349
C	9.988354	2.012931	9.389351
H	10.963474	1.765278	9.815479
C	4.250531	5.621217	7.364467
H	3.438667	5.096894	6.855108
C	6.305402	6.943517	8.708944
H	7.094854	7.483137	9.234992
C	5.207327	-0.555392	11.027629
H	4.132855	-0.667068	10.857613
C	7.953575	-0.299263	11.461777
H	9.027341	-0.192533	11.630344
C	5.030138	7.401025	12.548366
H	5.582364	7.284702	13.49145

H	4.296246	8.213171	12.658203
H	5.752294	7.674794	11.765108
C	1.791507	2.276069	7.908998
H	1.112943	2.331104	7.054537
C	3.132195	6.341743	10.728804
H	3.734699	6.814758	9.941566
H	2.382523	7.066708	11.079666
H	2.619956	5.477463	10.284983
C	8.251685	1.621896	7.765426
H	7.85925	1.066024	6.910876
C	3.023165	1.634962	7.782637
H	3.313872	1.187211	6.82956
C	9.489656	1.286218	8.30991
H	10.069071	0.462336	7.887495
C	7.406068	-1.545553	11.170355
H	8.050003	-2.426278	11.11327
C	6.031875	-1.673171	10.956305
H	5.600445	-2.652005	10.735172
C	3.01541	5.614095	13.478612
H	2.394753	4.721995	13.30907
H	2.361987	6.49445	13.568323
H	3.554192	5.469787	14.425524

Table S25. XYZ coordinates of DFT optimized geometry of $\mathbf{7_{os}}$

Symbol	X	Y	Z
Co	5.8370196	4.089778852	12.09482802
P	4.757387918	2.236569706	11.48963863
P	6.905184016	4.686836944	10.23439568
P	4.195357366	5.771242353	12.13089056
N	7.265993397	5.0016057	13.01323347
N	5.420530913	3.464216071	13.8656945
C	4.325658466	2.628443074	14.12396236
C	2.091452359	0.951527125	14.40237666
H	1.220091443	0.303771443	14.51627467
C	3.63256342	2.108387169	10.06517961
C	2.67720601	1.123345082	13.14969649
H	2.26830813	0.603309149	12.27889728
C	8.228268479	5.777437723	12.35506646
C	8.21181057	5.737362037	10.94566636
C	10.16116714	7.163866268	10.85176557
H	10.92267619	7.69217233	10.27497432
C	3.781071059	1.962420945	13.0067955
C	2.338041972	2.638543607	10.13785994
H	1.956756628	3.025903836	11.08705732

C	4.100004866	1.609143597	8.841327589
H	5.110878196	1.198912259	8.764876181
C	5.996698519	5.672278295	8.990973739
C	6.201802171	4.002250523	14.80882169
H	6.076215311	3.774170352	15.87063308
C	3.738225125	2.440226335	15.38247646
H	4.126953443	2.955682297	16.26240611
C	9.188952776	6.407526042	10.20499411
H	9.192921872	6.327839455	9.114150285
C	7.212399293	4.842884429	14.34371737
H	7.926427987	5.315435952	15.0228908
C	9.209718984	6.549066897	12.9979147
H	9.243092403	6.61547642	14.08629621
C	7.831597945	3.402578633	9.311141577
C	7.459328818	2.945266195	8.043075557
H	6.63645211	3.414941772	7.501795285
C	4.947230679	5.053125616	8.295248644
H	4.700122155	4.007021571	8.48498493
C	10.15678455	7.235930016	12.24726647
H	10.91453709	7.831045431	12.76252401
C	1.52526141	2.669137336	9.006181141
H	0.515242348	3.079605886	9.075252684
C	8.923549063	2.806180798	9.957628568
H	9.245312535	3.168049372	10.93894525
C	5.824880499	0.753814505	11.42871564
C	2.6291117	1.609418991	15.51034609
H	2.170923483	1.479536941	16.49362134
C	7.113260155	0.851240441	11.96351451
H	7.472832426	1.812469458	12.34125378
C	5.455977031	7.754517208	7.872002959
H	5.652759003	8.817873961	7.717802841
C	4.433786994	7.123147064	7.165958159
H	3.826631185	7.690662425	6.456973901
C	9.614572772	1.759772964	9.355347088
H	10.46454148	1.30516776	9.869247444
C	4.182522173	5.768069967	7.378799434
H	3.372245877	5.264264056	6.846695517
C	6.237953291	7.034566673	8.776214462
H	7.0322923	7.550467567	9.319267541
C	5.362584482	-0.487099972	10.97032203
H	4.351880057	-0.586011869	10.56508632
C	7.936234222	-0.271376378	12.02707218
H	8.940737635	-0.184925662	12.44766822
C	4.98959696	7.37267898	12.5334982

H	5.559320665	7.275250073	13.46864425
H	4.246391832	8.175857233	12.64632429
H	5.693499407	7.645895988	11.73379752
C	1.997254255	2.171928015	7.791504946
H	1.357921755	2.192586138	6.905807417
C	3.07426495	6.246741795	10.76104859
H	3.639913071	6.753780408	9.968638548
H	2.296070831	6.932393927	11.12788816
H	2.597535131	5.360056338	10.3217429
C	8.150462858	1.891859945	7.445048414
H	7.850527139	1.546190428	6.453009562
C	3.283837679	1.639798937	7.712021049
H	3.658337766	1.245310757	6.764583489
C	9.223021258	1.293015936	8.10090776
H	9.76250813	0.468710442	7.629154672
C	7.477786748	-1.497761719	11.55274506
H	8.123338536	-2.378068623	11.59687857
C	6.189710009	-1.604833343	11.02581314
H	5.823749594	-2.567432689	10.66148863
C	3.013202272	5.569532745	13.51673744
H	2.401271815	4.668402559	13.36496262
H	2.351043764	6.444020676	13.59831655
H	3.562052103	5.44769406	14.46085295

References

1 G. S. Day, B. Pan, D. L. Kellenberger, B. M. Foxman and C. M. Thomas, *Chem. Commun.*, 2011, **47**, 3634–3636.
2 R. A. Andersen, A. M. Bryan, M. Faust, P. P. Power, A. M. Bryan, P. P. Power, R. A. Andersen, M. Faust, P. P. Power and R. A. Andersen, in *Inorganic Syntheses*, John Wiley & Sons, Ltd, 2018, pp. 1–14.
3 V. W. Manner, T. F. Markle, J. H. Freudenthal, J. P. Roth and J. M. Mayer, *Chem. Commun.*, 2007, 256–258.
4 S. Stoll and A. Schweiger, *Journal of Magnetic Resonance*, 2006, **178**, 42–55.
5 F. Neese, *WIREs Computational Molecular Science*, 2022, **12**, e1606.
6 F. Neese, *WIREs Computational Molecular Science*, 2018, **8**, e1327.
7 F. Neese, *WIREs Computational Molecular Science*, 2012, **2**, 73–78.
8 J.-D. Chai and M. Head-Gordon, *Phys. Chem. Chem. Phys.*, 2008, **10**, 6615–6620.
9 F. Weigend, *Phys. Chem. Chem. Phys.*, 2006, **8**, 1057–1065.
10 F. Weigend and R. Ahlrichs, *Phys. Chem. Chem. Phys.*, 2005, **7**, 3297–3305.
